# Alveolar type II epithelial cell FASN maintains lipid homeostasis in experimental COPD

**DOI:** 10.1172/jci.insight.163403

**Published:** 2023-08-22

**Authors:** Li-Chao Fan, Keith McConn, Maria Plataki, Sarah Kenny, Niamh C. Williams, Kihwan Kim, Jennifer A. Quirke, Yan Chen, Maor Sauler, Matthias E. Möbius, Kuei-Pin Chung, Estela Area Gomez, Augustine M.K. Choi, Jin-Fu Xu, Suzanne M. Cloonan

**Affiliations:** 1 Division of Pulmonary and Critical Care Medicine, Joan and Sanford I. Weill Department of Medicine, Weill Cornell Medicine, New York, New York, USA.; 2 Department of Respiratory and Critical Care Medicine, Shanghai Pulmonary Hospital, School of Medicine, Tongji University, Shanghai, China.; 3 NewYork-Presbyterian Hospital, Weill Cornell Medicine, New York, New York, USA.; 4 School of Medicine, Trinity Biomedical Sciences Institute, and; 5 School of Physics, Trinity College Dublin, Dublin, Ireland.; 6 Pulmonary, Critical Care and Sleep Medicine, Yale School of Medicine, New Haven, Connecticut, USA.; 7 Department of Laboratory Medicine, National Taiwan University College of Medicine, Taipei, Taiwan.; 8 Department of Laboratory Medicine, National Taiwan University Hospital, Taipei, Taiwan.; 9 Division of Neuromuscular Medicine, Department of Neurology, Columbia University Irving Medical Center, Neurological Institute, New York, New York, USA.; 10 Center for Biological Research “Margarita Salas”, Consejo Superior de Investigaciones Científicas (CSIC), Madrid, Spain.

**Keywords:** Metabolism, Pulmonology, COPD, Intermediary metabolism, Pulmonary surfactants

## Abstract

Alveolar epithelial type II (AEC2) cells strictly regulate lipid metabolism to maintain surfactant synthesis. Loss of AEC2 cell function and surfactant production are implicated in the pathogenesis of the smoking-related lung disease chronic obstructive pulmonary disease (COPD). Whether smoking alters lipid synthesis in AEC2 cells and whether altering lipid metabolism in AEC2 cells contributes to COPD development are unclear. In this study, high-throughput lipidomic analysis revealed increased lipid biosynthesis in AEC2 cells isolated from mice chronically exposed to cigarette smoke (CS). Mice with a targeted deletion of the de novo lipogenesis enzyme, fatty acid synthase (FASN), in AEC2 cells (*Fasn*^iΔAEC2^) exposed to CS exhibited higher bronchoalveolar lavage fluid (BALF) neutrophils, higher BALF protein, and more severe airspace enlargement. *Fasn*^iΔAEC2^ mice exposed to CS had lower levels of key surfactant phospholipids but higher levels of BALF ether phospholipids, sphingomyelins, and polyunsaturated fatty acid–containing phospholipids, as well as increased BALF surface tension. *Fasn*^iΔAEC2^ mice exposed to CS also had higher levels of protective ferroptosis markers in the lung. These data suggest that AEC2 cell FASN modulates the response of the lung to smoke by regulating the composition of the surfactant phospholipidome.

## Graphical abstract

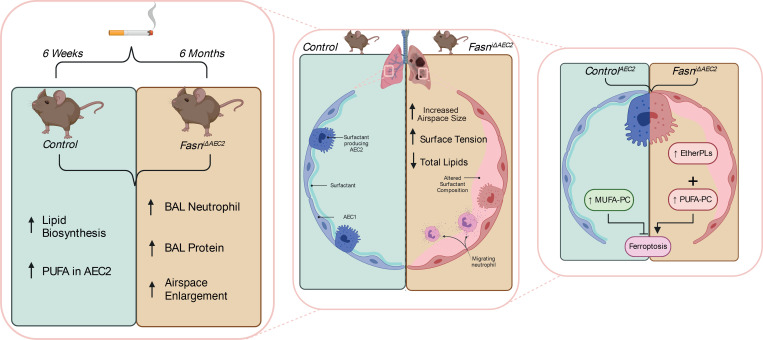

## Introduction

Chronic obstructive pulmonary disease (COPD) is characterized by inflammation of the airways, irreversible airflow obstruction, and destruction of the lung parenchyma or emphysema (1). As a main contributor to the global burden of disease, COPD represents the third leading cause of morbidity and mortality worldwide (2). The mechanisms underlying COPD pathogenesis remain incompletely understood but include oxidative stress, protease/antiprotease imbalance, and aberrant inflammatory responses to noxious gases such as cigarette smoke (CS), a primary risk factor for this disease (3).

Injury to or loss of alveolar epithelial type II (AEC2) cells represents a primary component of CS-induced emphysema resulting in alveolar airspace enlargement (4). AEC2 cells act as progenitors of AEC1 cells and are critical for surfactant production (5). Surfactant is not only synthesized but also stored, secreted, and recycled by AEC2 cells (6–8). Consisting of approximately 10% protein and 90% lipids (mainly saturated phospholipids), pulmonary surfactant serves to maintain alveolar surface tension and prevent alveolar collapse (9). AEC2 cells therefore use a complex set of lipid metabolic pathways to effectively adjust surfactant synthesis, secretion, and recycling in different physiologic situations (10).

A number of studies have implicated a disturbance in lipid homeostasis by smoke and in COPD, including increases in fatty acid (FA) oxidation, increases in sphingolipid production (11–17), and a loss in the availability of the key surfactant phospholipids phosphatidylcholine (PC) and phosphatidylglycerol (PG) (11). However, whether CS exposure leads to the dysregulation of lipid metabolism specifically in AEC2 cells and how this contributes to COPD pathogenesis remain unclear.

In mammals, the enzyme fatty acid synthase (FASN) provides a key step in the synthesis of FAs by condensing acetyl-CoA and malonyl-CoA to generate palmitate (18). FASN functions as a central regulator of lipid metabolism and is capable of rewiring cells for greater energy flexibility to adapt to new high-energy requirements (19). FA synthesis and FASN are essential to the correct development of the fetal lung and the normal functionality of the adult lung. FASN is also required for surfactant production of alveolar epithelial cells (20–23). Variations in FA synthesis, intake, elongation, and desaturation also affect PC composition (24). We have previously observed increased *FASN* gene expression in COPD lungs, using serial analysis of gene expression (25). However, the role of FASN in AEC2 cell function in COPD remains to be defined.

In the current study, we show that lipid composition and regulation by FASN in AEC2 cells are critical in the response of the AEC2 cell to smoke. Mice with targeted conditional deletion of FASN in AEC2 cells (*Fasn*^iΔAEC2^) were more susceptible to smoke- and age-induced airspace enlargement. *Fasn*^iΔAEC2^ mice exposed to smoke had lower levels of key surfactant bronchoalveolar lavage fluid (BALF) phospholipids but higher levels of BALF ether phospholipids and sphingomyelin species and higher surface tension when compared with controls exposed to room air. *Fasn*^iΔAEC2^ mice exposed to smoke also had higher levels of polyunsaturated fatty acid–containing (PUFA-containing) phospholipids and higher expression of protective markers of ferroptosis, a form of iron-dependent cell death via lipid peroxidation, in the lung. These data suggest that AEC2 cell FASN contributes to the regulation of the lung’s response to smoke by regulating surfactant-associated phospholipid metabolism. We hypothesize that this may represent an essential mechanism engaged by the AEC2 cell to maintain correct surfactant composition and membrane integrity in response to smoke.

## Results

### In vivo CS exposure increases lipid biogenesis and alters the composition of the surfactant phospholipidome in AEC2 cells.

Of the lipids found in surfactant, 90% are phospholipids. Surfactant lipids are characterized by high levels of saturated FA chains, such as dipalmitoyl phosphatidylcholine (DPPC, PC 32:0), which comprises the main lipid species responsible for the surface tension reduction properties of surfactant (26). PG also represents a major component of surfactant, with other phospholipids present in lower abundance. Little is currently known about the role of lipid metabolism in AEC2 cells and COPD pathogenesis.

**Figure 1:**
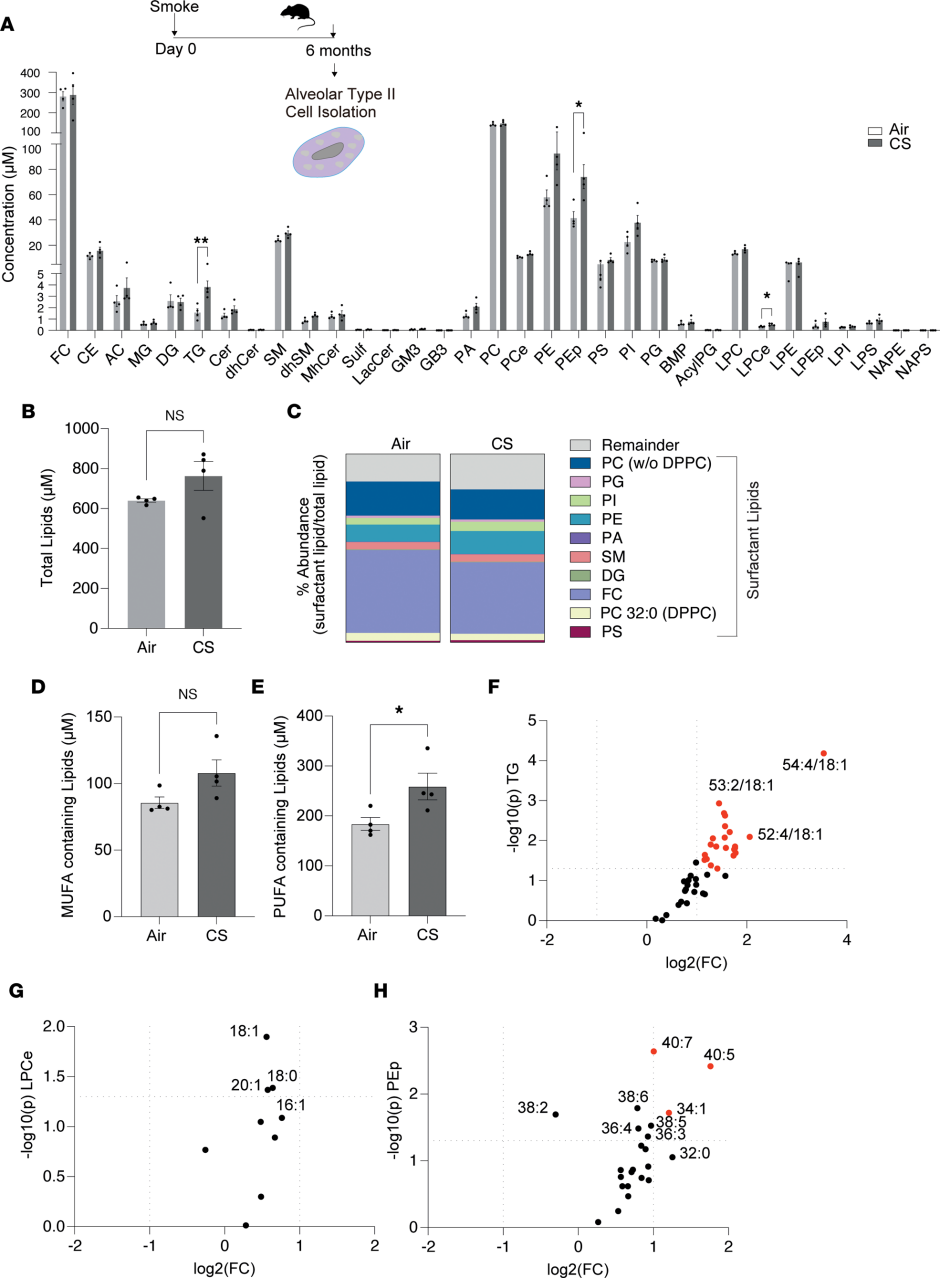
Comprehensive lipidomic analysis of primary AEC2 cells from mice exposed to CS for 6 months. (**A**–**C**) Schematic for the timeline of smoke exposure for the isolation of AEC2 cells for lipidomic analysis with individual lipid compositions (**A**), total lipid levels (**B**), and percentage abundance of surfactant-related lipids (calculated by expressing the concentration of each lipid family as a percentage of total lipids) (**C**) in isolated AEC2 cells from mice exposed to CS or air for 6 months (*n* = 4 mice per group). FC, free cholesterol; CE, cholesterol ester; AC, acyl carnitine; MG, monoacylglycerol; DG, diacylglycerol; TG, triacylglycerol; Cer, ceramide; dhCer, dihydroceramide; MhCer, monohexosylceramide; Sulf, sulfatide; LacCer, lactosylceramide; GM3, monosialodihexosylganglioside; GB3, globotriaosylceramide; BMP, bis(monoacylglycero)phosphate; AcylPG, acyl phosphatidylglycerol; SM, sphingomyelin; PA, phosphatidic acid; PC, phosphatidylcholine; PCe, phosphatidylcholine ether; PE, phosphatidylethanolamine; PEp, plasmalogen phosphatidylethanolamine; PS, phosphatidylserine; PI, phosphatidylinositol; PG, phosphatidylglycerol; LPC, lysophosphatidylcholine; LPE, lysophosphatidylethanolamine; LPEp, plasmogen lysophosphatidylethanolamine; LPI, lysophosphatidylinositol; LPS, lysophosphatidylserine; NAPE, N-acyl phosphatidylethanolamine; NAPS, N-acyl phosphatidylserine. (**D** and **E**) Concentration of MUFA- (**D**) and PUFA- (**E**) containing lipids in isolated AEC2 cells from mice exposed to CS or air for 6 months. (**F**–**H**) Volcano plots of altered TG species (**F**), ether lysophosphatidylcholines (LPCes) (**G**), and plasmalogen phosphatidylethanolamines (PEps) (**H**) in isolated AEC2 cells from mice exposed to CS or air for 6 months (*n* = 4 mice per group). Red dots denote *P* < 0.05 fold-change of +2. Data representative of 1 independent experiment with *n* = 4 mice per group and presented as mean ± SEM (*P < 0.05, **P < 0.01, by Student’s unpaired *t* test).

Using a well-established experimental model of CS-induced injury (27–29), we investigated changes in lipid composition that occur specifically in AEC2 cells in response to chronic CS exposure (6 months). We isolated AEC2 cells (90% purity) (Supplemental Figure 1A; supplemental material available online with this article; https://doi.org/10.1172/jci.insight.163403DS1) from the lungs of mice that were exposed to CS or room air for 6 months and performed comprehensive (572 individual lipid species) lipidomic analysis using liquid chromatography/mass spectrometry (LC/MS). As expected, the most abundant classes of lipids detected in AEC2 cells were phospholipids and free cholesterol (FC), followed by sphingolipids and glycerolipids (Figure 1A). While AEC2 cells from CS-exposed mice displayed a global trend for higher total lipid levels (Figure 1B), the abundance (or ratio of each lipid as a percentage of total lipids) of the major classes of lipids important for surfactant composition were significantly lower (*P* = 0.027 by Student’s unpaired *t* test) in the smoke-exposed cells when compared with air controls (Figure 1C). This was attributed to the trend of lipid composition of smoke-exposed cells having a lower abundance of FC and PC. AEC2 cells isolated from smoke-exposed mice also had higher levels of lipids containing monounsaturated fatty acids (MUFAs) and PUFA, when compared with air-exposed controls (Figure 1, D and E).

Out of the 33 classes of lipids analyzed, 3 were significantly increased by smoke when compared with air-exposed controls. Specifically, triacylglycerol (TG), ether lysophosphatidylcholine (LPCe), and plasmalogen phosphatidylethanolamine (PEp) classes were all significantly higher (*P* < 0.05) relative to AEC2 cells from air-exposed mice (Figure 1A). TG molecules are a major form of storage and transport of FAs and act as a pool for structural and bioactive FAs. Here, TGs showed a marked 2.4-fold increase in AEC2 cells in response to CS. Further analysis revealed that out of the 41 TG species analyzed, 23 species were significantly increased by CS in AEC2 cells when compared with air controls (Figure 1F).

LPCe and PEp classes, known as ether phospholipids (etherPLs), are composed of a glycerol backbone with an alkyl chain (ether bond) in the sn-1 position, a FA in the sn-2 position, and a polar headgroup in the sn-3 position of the glycerol backbone, which generally consists of ethanolamine or choline (30). In this study, CS exposure resulted in significant increases in several etherPLs, including LPCe (LPCe 18:0, LPCe 18:1, and LPCe 20:1) and PEp species (PEps 34:1, 36:4, 38:5, 38:6, 40:5, and 40:7) (Figure 1, G and H).

Despite the overall ratio of the abundance of surfactant-related lipids to total lipids declining, individual species of phosphatidic acid (PA 30:0, 32:0, 32:1, 34:1, 36:0, and 34:2), PG (36:2, 38:1), phosphatidylinositol (PI 34:1, 34:2, 36:0, 36:4, 38:4), phosphatidylethanolamine (PE 38:2, 36:0, 36:4), phosphatidylserine (PS 38:6), and cholesterol ester (CE 20:1, 20:2, 22:3, 22:4, 24:5, 22:5) species increased in AEC2 cells upon exposure to smoke, when compared with air (Supplemental Figure 1B). Finally, we found increases in several ceramide (Cer) and sphingomyelin (SM) species in AEC2 cells exposed to smoke, which have been previously reported as elevated in COPD patient lung tissue and cells (25, 31). Specifically, the levels of species of SM d18:1/16:1, d18:1/20:0, and d18:1/20:1; dihydrosphingomyelin (dhSM) d18:1/16:0, d18:1/18:1, and d21:1/24:1; dihydroceramide (dhCer) 18:0/22:1; and Cer d18:1/22:1 were all significantly increased by CS (Supplemental Figure 1B). Taken together, the above data demonstrate that in response to smoke, the lipidome of AEC2 cells changes, with an overall decline in abundance of surfactant-related lipids and a concomitant increase in TGs, etherPLs, and SM species as well as lipid species bound to PUFAs. These changes may have important ramifications for surfactant composition and production by the AEC2 cell.

### FASN is regulated by CS in AEC2 cells.

FAs for etherPL or triglyceride synthesis are derived from FASN-mediated de novo lipogenesis (30, 32). In the lung, FASN is predominantly expressed in AEC2 cells (20, 33). Moreover, de novo FA synthesis via FASN is essential for surfactant secretion (21–23). However, little is known regarding the role of FASN in the lipogenic response of AEC2 cells to smoke or the role of AEC2 cell FASN in COPD pathogenesis.

**Figure 2:**
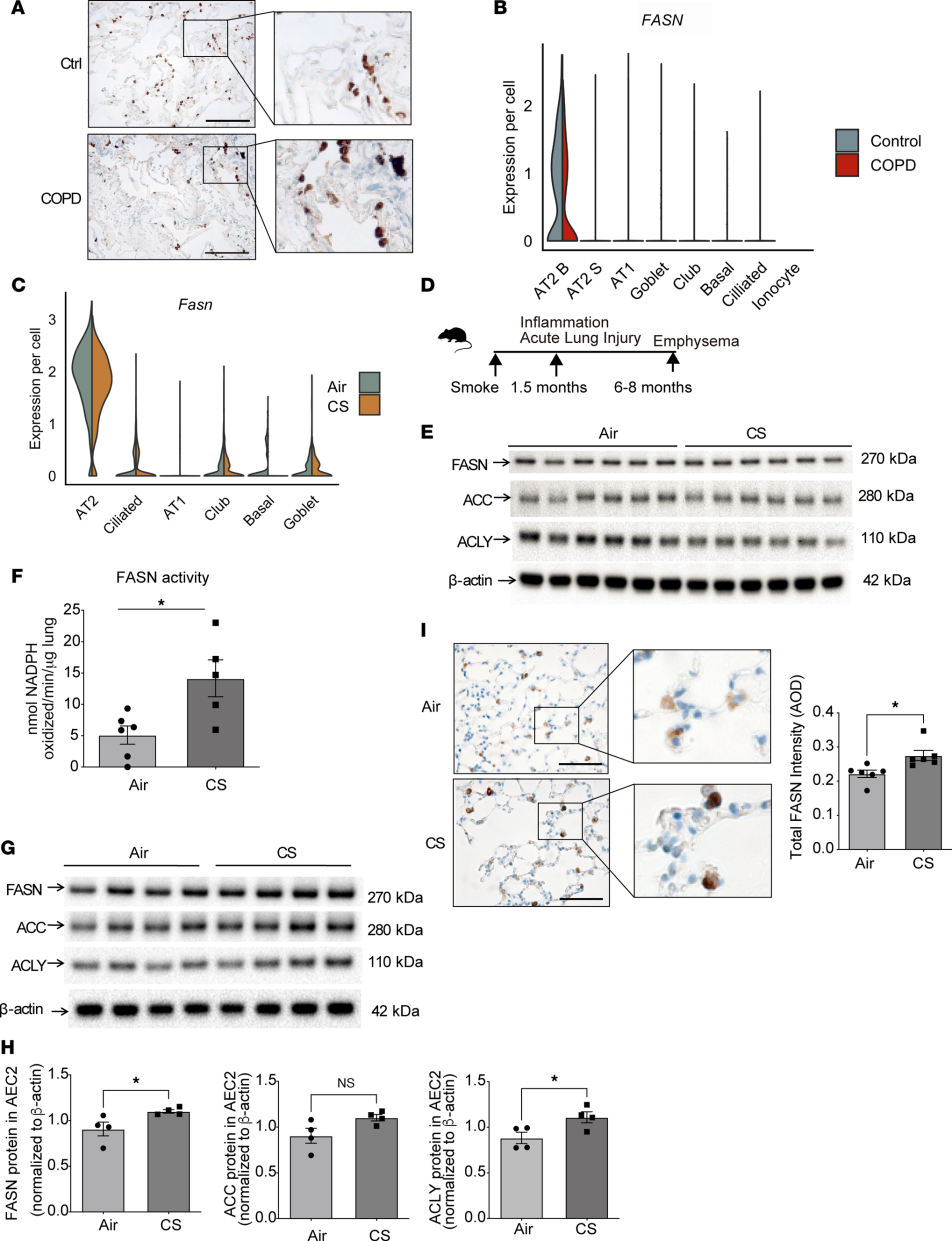
FASN is localized to AEC2 cells and is regulated by CS. (**A**) Representative immunohistochemical (IHC) stain of FASN in lung sections from healthy control (Ctrl) and chronic obstructive pulmonary disease (COPD) patients (*n* = 3 per group). Scale bars, 200 μm. Insets, original magnification, ×8. (**B** and **C**) Violin plots of normalized expression values for FASN in human (**B**) and murine (**C**) lung cells. “AT1” refers to alveolar epithelial type I cells; “AT2B” refers to alveolar epithelial type II cells associated with canonical “bulk” AT2 markers such as *SFTPA1*, *SFTPA2*, and *ETV5*; and “AT2S” refers to AEC2 cells that are more stem-like with increased expression of *ERBB4*, *TNIK*, *TCF12*, *FOXP1*, *STAT3*, *YAP*, and *TEAD1*. (**D**) Schematic for the timeline of CS exposure. (**E**) Representative immunoblot of FASN, acetyl-CoA carboxylase (ACC), ATP citrate lyase (ACLY), and β-actin (*n* = 6 per group) expression in murine lung tissues from C57BL/6 mice exposed to CS or air for 6 weeks. Immunoblotting for FASN and β-actin was carried out on the same gel/membrane; immunoblotting for ACC and ACLY was carried out on the same gel/membrane. The same samples were used to load both gels. See complete unedited blots in the supplemental material. (**F**) FASN enzymatic activity (*n* = 6 in air group, *n* = 5 in CS group) in murine lung tissues from C57BL/6 mice exposed to CS or air for 6 weeks. (**G**) Immunoblotting of FASN, ACC, and ACLY expression in primary AEC2 cells isolated from C57BL/6 mice exposed to CS or air for 6 months (*n* = 4 per group) with quantification (**H**). Immunoblotting for ACC and ACLY were carried out on the same gel/membrane. FASN and β-actin were run on separate gels. The same samples were used to load all gels. (**I**) Representative IHC staining of FASN in lung sections from C57BL/6 mice exposed to CS or air for 6 months (*n* = 3 per group) with corresponding quantification. Scale bars, 200 μm. Insets, original magnification, ×8. AOD, average optical density. Data are presented as mean ± SEM; *P < 0.05 by Student’s unpaired *t* test.

First, we verified using immunohistochemical (IHC) staining of FASN in the lungs of healthy controls and individuals with COPD that FASN is enriched in AEC2 cells in the human lung (Figure 2A). These data were supported by analyzing available single-cell RNA-sequencing (scRNASeq) profiles of explanted lung tissue from patients with advanced COPD or control lungs, which demonstrated that FASN was predominantly enriched in a subpopulation of surfactant-producing AEC2 cells (associated with canonical “bulk” AEC2 markers such as *SFTPA1*, *SFTPA2*, and *ETV5* and termed AT2B cells) (34) (Figure 2B and Supplemental Figure 2A). Similarly, IHC and immunofluorescence staining of pro-surfactant protein C (pro-SPC; a marker of AEC2 cells) and FASN in the lung sections from C57BL/6 mice revealed that FASN colocalized with pro-SPC, verifying that FASN was also enriched in murine AEC2 cells (Supplemental Figure 2, C and D). These data were supported by analyzing available scRNA-Seq data from murine lung tissue, which showed that FASN is predominantly localized to AEC2 (or AT2) cells (Figure 2C and Supplemental Figure 2B) (34). FASN expression in lung tissue was relatively higher compared with that observed in kidney, heart, muscle, and spleen and second to liver as assessed by immunoblotting (Supplemental Figure 2E).

Notably, reanalysis of scRNA-Seq data of human lung tissue found *FASN* to be decreased specifically in AT2B cells from individuals with COPD (–0.34 log_2_ fold-change [log2FC], *P* = 2.29 × 10^–5^) (Figure 2B). Therefore, we next assessed the expression and regulation of FASN in AEC2 cells of the lung in experimental COPD models. Using a well-established experimental acute model of CS-induced lung injury (27–29), we examined FASN protein levels by immunoblotting. While there was no significant difference in the protein levels of FASN in whole lung homogenates from mice exposed to CS for 6 weeks when compared to air controls, FASN enzymatic activity was significantly higher in whole lung homogenates after 6 weeks of CS exposure (Figure 2, D–F). In a chronic (6–10 months) CS-induced injury model (29, 35), scRNA-Seq analysis showed *Fasn* to be significantly (log2FC –0.24, *P* = 6.39 × 10^–9^, FDR < 0.0005) decreased in AEC2 cells exposed to 10 months of smoke (Figure 2, C and D). This is consistent with FASN protein and gene expression being lower in whole lung homogenates isolated from mice exposed to 6 months of smoke when compared with air controls. Protein levels of the enzyme acetyl-CoA carboxylase (ACC), which catalyzes the carboxylation of acetyl-CoA to malonyl-CoA, the rate-limiting step in FA synthesis, and levels of the enzyme ATP citrate lyase (ACLY), which synthesizes cytosolic acetyl-CoA, were also lower in whole lung homogenates isolated from mice exposed to 6 months of smoke when compared with air controls (Supplemental Figure 2, F and G).

Conversely, in isolated AEC2 cells from mice exposed to CS for 6 months, FASN protein expression was increased when compared with air controls by IHC staining and immunoblotting. Similarly, the expression level of ACLY was also higher in AEC2 cells in response to smoke (Figure 2, G–I). Taken together, these results show that FASN transcript is lower in surfactant-producing AEC2 type B cells in COPD and in response to chronic smoke exposure in mice, but FASN protein and activity as well as other enzymes essential for FA synthesis are upregulated in primary isolated AEC2 cells in response to smoke.

### Targeted deletion of FASN in AEC2 cells alters the lipid composition and transcriptomic profile of the lung.

**Figure 3:**
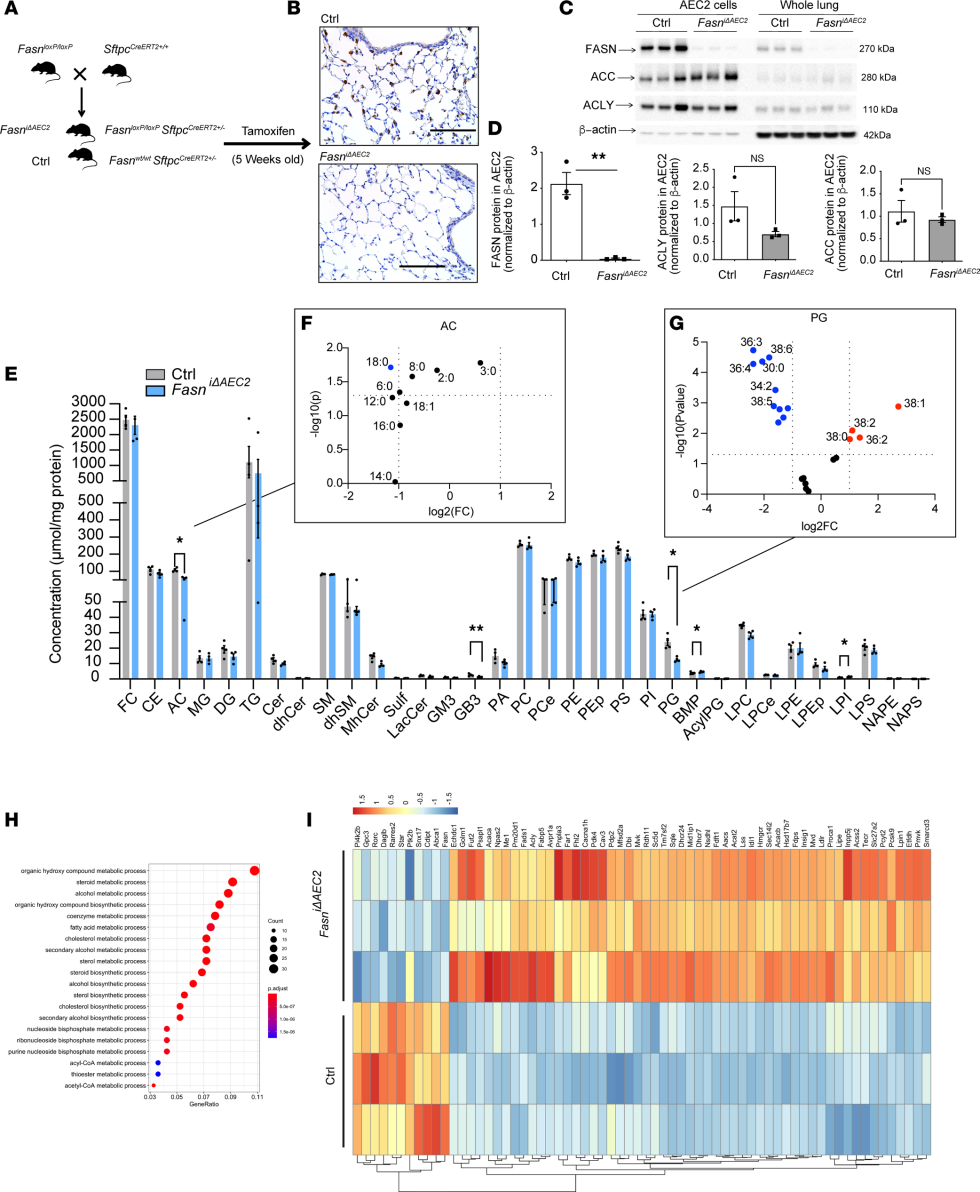
Generation of *Fasn*^iΔAEC2^ mice with altered lung lipid levels. (**A**) *Fasn^fl/fl^* and *Sftpc-Cre^+/+^* mice were crossed to generate *Fasn*^iΔAEC2^ and control *Sftpc^CreERT2+/–^* mice, and both were induced by tamoxifen to drive CreERT2. (**B**) Representative IHC staining of FASN in lung tissue from *Fasn*^iΔAEC2^ and control *Sftpc^CreERT2+/–^* mice (*n* = 4 per group). Scale bars, 100 μm, (*n* = 3 experiments). (**C** and **D**) Representative immunoblot (*n* = 3 experiments) (**C**) with quantification (**D**) of FASN, ACLY, and ACC protein expression in primary isolated AEC2 cells and whole lung homogenates from *Fasn*^iΔAEC2^ and control *Sftpc^CreERT2+/–^* mice (*n* = 3 per group). **P < 0.01 by Student’s unpaired *t* test. Immunoblotting for FASN, ACLY, and β-actin was carried out on the same membrane; immunoblotting for ACC was carried out on a separate membrane. The same samples were used to load both gels. (**E**–**G**) Lipidomic profiling (**E**) of whole lung lipid extracts from *Fasn*^iΔAEC2^ and control *Sftpc^CreERT2+/–^* mice with subgroup analysis of (**F**) acyl carnitine (AC) and (**G**) phosphatidylglycerol (PG) species. Red dots denote *P* < 0.05 fold-change of +2; blue dots denote *P* < 0.05 fold-change of –2. Data are expressed as mean ± SEM. (*P < 0.05, **P < 0.01, by Student’s unpaired *t* test, *n* = 3 mice per group of 1 independent experiment.) (**H**) Gene ontology pathway analysis of lipid metabolic processes of significantly altered genes represented as a (**I**) heatmap from whole lung transcriptomic profiling of *Fasn*^iΔAEC2^ and control *Sftpc^CreERT2+/–^* mice (*n* = 3 mice per group, 1 independent experiment).

To identify the specific role of FASN and enhanced lipid production in AEC2 cells by smoke, we selectively deleted FASN in murine AEC2 cells. Genetically modified mice harboring *Fasn* flanked by 2 *loxP* sites were crossed with *Sftpc^CreERT2+/+^* mice (*Fasn*^iΔAEC2^) in which *Fasn* was selectively deleted in AEC2 cells after administration of tamoxifen (Figure 3A). IHC analysis at 2 weeks after tamoxifen injection demonstrated that FASN was substantially lower in AEC2 cells from *Fasn*^iΔAEC2^ mouse lung tissue (Figure 3B). Consistent with these IHC data, immunoblotting showed that FASN levels were significantly decreased in isolated AEC2 cells of *Fasn*^iΔAEC2^ mice relative to *Sftpc^CreERT2+/–^* controls. The expression levels of ACLY and ACC were not significantly altered in isolated AEC2 cells of *Fasn*^iΔAEC2^ mice relative to *Sftpc^CreERT2+/–^* controls (Figure 3, C and D, and Supplemental Figure 3A). Notably, targeted deletion of FASN in AEC2 cells resulted in a decrease of FASN in whole lung tissue, verifying that, in the healthy lung, FASN was largely localized to AEC2 cells. Similarly, *Fasn* mRNA expression was decreased by 64% in total lung in *Fasn*^iΔAEC2^ mice and by approximately 90% in purified AEC2 cells isolated from *Fasn*^iΔAEC2^ mice, relative to the lungs and cells from control *Sftpc^CreERT2+/–^* mice. However, the expression of the lipid-related genes *Scd1*, *Fdps1*, *Fabp5*, and *Thrsp* was significantly increased in both lung tissue and isolated primary AEC2 cells in the *Fasn*^iΔAEC2^ mice compared with *Sftpc^CreERT2+/–^* controls (Supplemental Figure 3B).

To verify that loss of FASN in AEC2 cells alters the lipidome of the lung, we assessed the lipid profile of the lungs of *Fasn*^iΔAEC2^ mice. A loss of FASN in AEC2 cells resulted in a nonsignificant trend for a loss of total lipid levels in the lung tissue (Supplemental Figure 3C). There was a notably significant decrease in acylcarnitine (AC), globotriaosylceramide (GB3), and PG species in whole lung tissue compared with control *Sftpc^CreERT2+/–^* mice (Figure 3E). In contrast, the content of bis(monoacylglycero)phosphate (BMP) and lysophosphatidylinositol (LPI) was significantly higher in *Fasn*^iΔAEC2^ lungs relative to control mice (Figure 3E).

ACs formed from carnitine and acyl-CoAs are considered the transport form of FAs and can be used for energy production in mitochondria via β-oxidation or for the synthesis of endogenous molecules (36). In this study, several AC species were lower in *Fasn*^iΔAEC2^ mice when compared with the control *Sftpc^CreERT2+/–^* mice (Figure 3F). PGs are present in relatively high amounts (~10%) in surfactant, and they play an important role in lung host defense by regulating innate immune processes (37). In this study, several PG species were lower in *Fasn*^iΔAEC2^ mice when compared with the control *Sftpc^CreERT2+/–^* mice (Figure 3G). BMP, a structural isomer of PG, is a negatively charged glycerophospholipid that is mainly localized in late endosomes/lysosomes (38). A number of BMP species were increased or decreased in the *Fasn*^iΔAEC2^ mice when compared with the control *Sftpc^CreERT2+/–^* mice (Supplemental Figure 3D). Finally, LPI is a bioactive lipid generated by the phospholipase A family of lipases that is believed to play an important role in cell growth, differentiation, and motility in a number of cell types (39). In this study, a number of LPI species were increased in the *Fasn*^iΔAEC2^ mice when compared with the control *Sftpc^CreERT2+/–^* mice (Supplemental Figure 3E).

RNA-Seq on whole lung tissue from *Fasn*^iΔAEC2^ and control *Sftpc^CreERT2+/–^* mice (8 weeks after tamoxifen) demonstrated that 678 genes were significantly altered (adjusted *P* value < 0.05, fold-change 0.2) between *Fasn*^iΔAEC2^ and control mouse lung. Ingenuity Pathway Analysis (QIAGEN) on these 678 genes revealed that the top significantly altered canonical pathways included cholesterol biosynthesis (*P* = 1.12 × 10^–15^) and EIF2 signaling (*P* = 8.91 × 10^–8^), with the top molecular and cellular function pathways being cell death and survival (*P* = 2.37 × 10^–4^ to 3.41 × 10^–16^), lipid metabolism (3.23 × 10^–4^ to 4.77 × 10^–14^), small molecule biochemistry (*P* = 3.23 × 10^–4^ to 4.77 × 10^–14^), cell morphology (*P* = 2.55 × 10^–4^ to 2.11 × 10^–13^), and cellular function and maintenance (*P* = 2.55 × 10^–4^ to 5.83 × 10^–12^). Gene ontology analysis identified enrichment of genes in several key pathways involved in lipid and FA biosynthesis that were altered in the *Fasn*^iΔAEC2^ mice when compared with control *Sftpc^CreERT2+/–^* mice (Figure 3H). Specifically, a number of genes were upregulated, including those involved in FA biosynthesis pathways (*Acaca*, *Fads1*, *Acly*, *Aacs*, *Acss2*, *Tecr*, *Slc27a2*, *Pcyt2*, *Lpin1*, *Me1*), cholesterol biosynthesis (*Fdps*, *Sc5d*, *Mvk*, *Tm7sf2*, *Dhcr24*, *Nsdhl*, *Fdft1*, *Acat2*, *Lss*, *Idi1*, *Hmgcr*, *Sec14l2*, *Hsd17b7*, *Insig1*, *Mvd*, *Lipe*), steroid biosynthesis (*Sqle*), lipid transport (*Fabp5*, *Dbi*, *Ldlr*), β-oxidation (*Echdc1*, *Pm20d1*, *Acacb*), lysosomal degradation of sphingolipids (*Psapl1*), reduction of FAs for ether lipid synthesis (*Far1*), and regulation of pyruvate dehydrogenase (*Pdk4*, *Pdp2*) (Figure 3I). Taken together, these results suggest that FASN is localized predominantly in AEC2 cells of wild-type mice compared with other cells in the alveolar space and that the successful targeted deletion of *Fasn* in AEC2 cells in mice reduces several lipid species important for surfactant lipid composition.

### Targeted deletion of FASN in AEC2 cells results in accelerated age-associated increased airspace size, which may be partially restored by dietary TGs.

**Figure 4:**
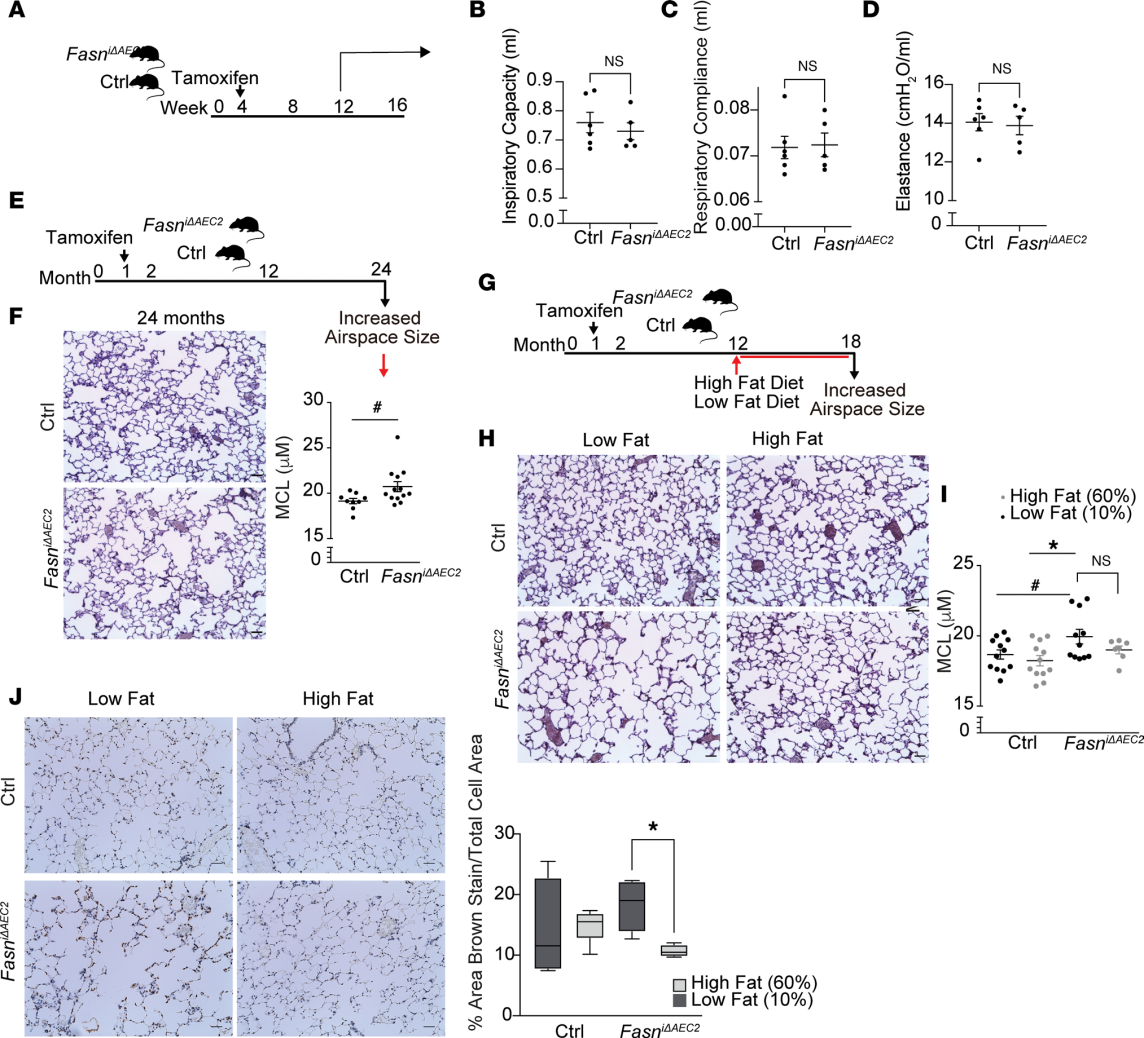
Targeted deletion of FASN in AEC2 cells results in age-associated airspace enlargement. (**A**–**D**) (**A**) Schematic of timeline for inspiratory capacity (**B**), compliance (**C**), and elastance (**D**) of 13- to 14-week-old *Fasn*^iΔAEC2^ and *Sftpc^CreERT2+/–^* control mice (*n* = 5–6 per group). (**E**) Schematic and (**F**) representative IHC modified gills-stained lung sections (*left*) with mean chord length (MCL) values (*right*) of 24-month *Sftpc^CreERT2+/–^* control and *Fasn*^iΔAEC2^ mice (Ctrl *n* = 9; *Fasn*^iΔAEC2^ *n* = 13). (**G**) Schematic of *Fasn*^iΔAEC2^ and control *Sftpc^CreERT2+/–^* mice fed with high-fat (60%) or low-fat (10%) diet at age 12 months until sacrifice at 18 months. (**H**) Representative IHC modified gills-stained lung sections and (**I**) MCL values with (**J**) representative TUNEL stain and corresponding quantification (*right*) from *Fasn*^iΔAEC2^ and control *Sftpc^CreERT2+/–^* mice at 18 months upon supplementation with a high-fat (60%) or low-fat (10%) diet in lung tissue and MCL scoring of mouse lungs for each group. (Ctrl low fat, *n* = 12; *Fasn*^iΔAEC2^ low fat, *n* = 11, Ctrl high fat, *n* = 12, *Fasn*^iΔAEC2^ high fat, *n* = 7.) Data represented as mean ± SEM of 1 independent experiment. *P < 0.05 by 1-way ANOVA, ^#^*P* < 0.05 by Student’s unpaired *t* test.

We next assessed if deletion of FASN in AEC2 cells resulted in any overt baseline pathology in the lung over time. At 13–14 weeks after tamoxifen treatment, *Fasn*^iΔAEC2^ mice had similar lung inspiratory capacity, compliance, and elastance when compared to *Sftpc^CreERT2+/–^* mice (Figure 4, A–D). However, aged (24-month-old) *Fasn*^iΔAEC2^ mice had significantly higher alveolar airspace diameters as quantified by mean chord length (MCL), with no change in overall lung area (40), suggestive of accelerated age-associated increased airspace size (41) (Figure 4, E and F, and Supplemental Figure 3F). Given that the loss of FASN in AEC2 cells in the lung resulted in loss of several essential lipid species, we determined whether adding specific lipids in the diet could alter the progression of age-induced increased airspace size in vivo. We found that 12-month-old *Fasn*^iΔAEC2^ mice administered a diet rich in TGs (60% fat derived from lard) had lower MCL values compared with 12-month-old *Fasn*^iΔAEC2^ mice administered a low-lipid (10% lipid) diet for 6 months. However, while a trend was observed, no significant difference was noted between these 2 groups (Figure 4, G–I), with no difference between the lung areas of each group (Supplemental Figure 3G). Conversely, *Fasn*^iΔAEC2^ mice administered a diet rich in TGs had a significantly lower percentage of TUNEL-positive stain per total cell area when compared with controls, suggesting some beneficial effects (Figure 4J).

### Targeted deletion of FASN in AEC2 cells results in increased lung injury, inflammation, and airspace enlargement upon acute or chronic smoke exposure.

**Figure 5:**
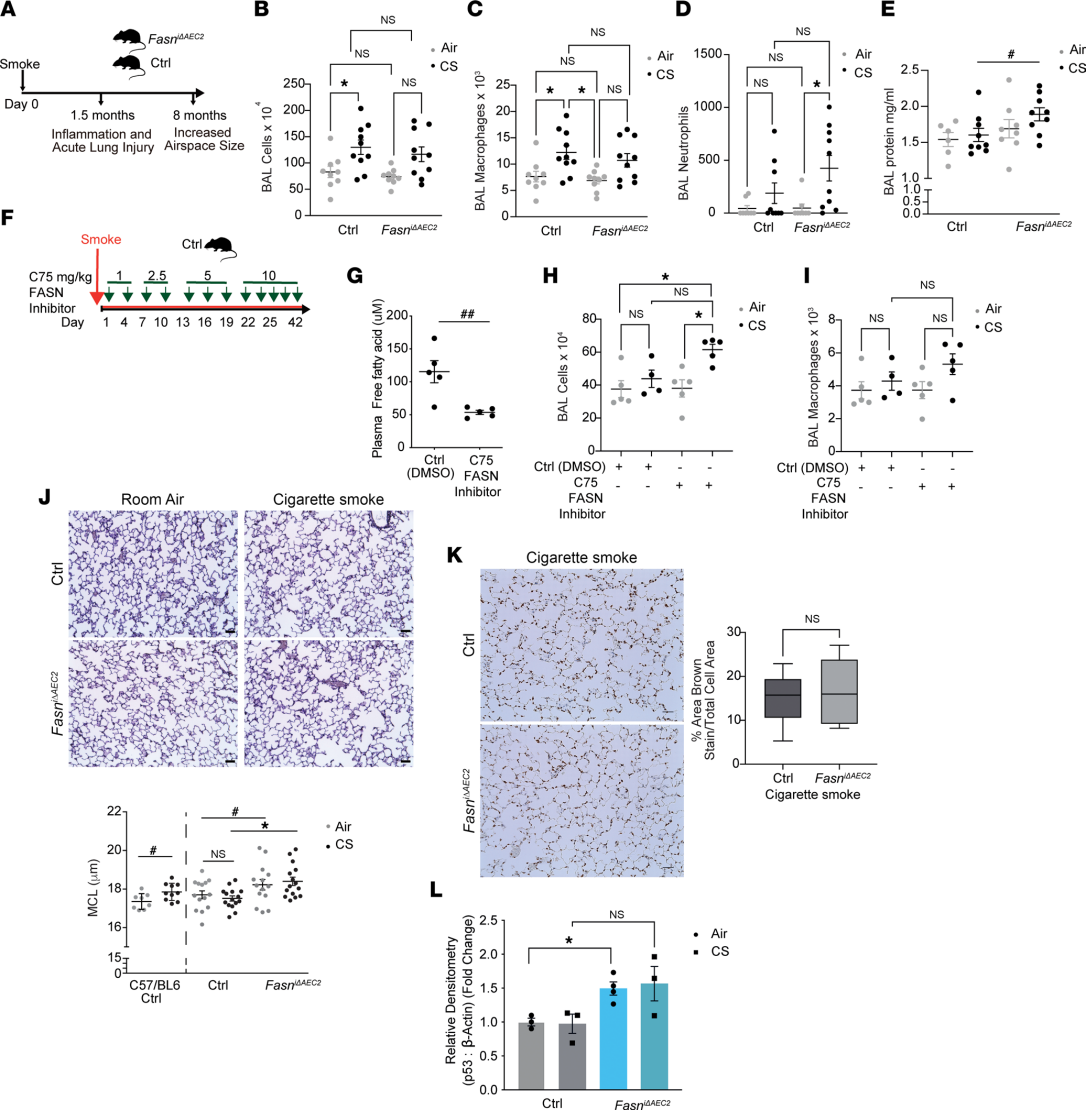
Targeted deletion of FASN in AEC2 cells results in increased lung injury, inflammation, and airspace enlargement upon acute or chronic smoke exposure. (**A**) Schematic of timeline of *Fasn*^iΔAEC2^ and control *Sftpc*^CreERT2+/–^ mice exposed to acute (6 weeks) or chronic (8 months) CS exposure. (**B**) Total BALF leukocyte counts, (**C**) total macrophage counts, (**D**) total neutrophil counts (*n* = 9–10 mice per group), and (**E**) total protein concentrations in BALF (*n* = 6–9 mice per group, *n* = 2 technical replicates) of *Fasn*^iΔAEC2^ and control *Sftpc^CreERT2+/–^* mice after acute (6 weeks) CS exposure. (**F**) Schematic of treatment regimen with the FASN inhibitor C75 and smoke. (**G**) Plasma free fatty acid levels (*n* = 5 mice per group), (**H**) total BALF leukocytes, and (**I**) total BALF macrophages of mice administered C75 (1 mg/kg 1–7 days, 2.5 mg/kg days 7–13, 5 mg/kg days 14–22, then 10 mg/kg days 23–42, DMSO as control) for a total of 42 days of exposure to CS. (*n* = 4–5 mice per group.) (**J**) Representative IHC images of modified gills-stained lung sections (*top*) and MCL scoring (*bottom*) of mouse lungs for each group (*n* = 8–15 mice per group) calculated from *Fasn*^iΔAEC2^ and *Sftpc^CreERT2+/–^* control mice, as well as C57BL/6 controls exposed to 8 months of CS exposure. (**K**) Representative IHC image of TUNEL stain of lung tissue from *Fasn*^iΔAEC2^ and *Sftpc^CreERT2+/–^* control mice exposed to 8 months of CS with corresponding quantification norm (*right*). (**L**) Densitometric analysis of fold-change in p53 expression by immunoblotting in the whole lung tissue of *Fasn*^iΔAEC2^ and *Sftpc^CreERT2+/–^* control mice exposed to 6 months of CS (*n* = 3 mice per group) normalized to β-actin. Scale bars, 50 μm. Data represented as mean ± SEM of 1 independent experiment. *P < 0.05 by 1-way ANOVA followed by Tukey’s correction. ^#^*P* < 0.05 by Student’s unpaired *t* test.

Our data show that loss of FASN in AEC2 cell in the lung alters the lipid profile of the lung, which could accelerate age-induced airspace enlargement. Thus, we next wished to assess the role of FASN in AEC2 cells in the response of the lung to smoke. Acute smoke exposure (6 weeks) of *Fasn*^iΔAEC2^ mice resulted in increased leukocyte infiltration in the BALF of the lung, similar to the *Sftpc^CreERT2+/–^* controls exposed to CS (Figure 5, A and B). These infiltrating leukocytes consisted predominantly of macrophages in both animal models exposed to CS, although *Fasn*^iΔAEC2^ also showed higher levels of infiltrating BALF neutrophils when compared with *Fasn*^iΔAEC2^ mice exposed to room air (Figure 5, C and D). *Fasn*^iΔAEC2^ mice also displayed more injury characterized by higher BALF protein levels when compared with *Sftpc^CreERT2+/–^* controls exposed to CS, suggestive of decreased lung barrier function (Figure 5E).

Next, we pharmacologically targeted FASN in the lung by instilling the synthetic cell-permeable α-methylene-γ-butyrolactone FASN inhibitor C75 (42) (Figure 5F). As expected, intraperitoneal (I.P.) injection of C75 into mice for 42 days resulted in a decline in circulating free FAs in plasma (Figure 5G). Animals administered I.P. C75 and exposed to 42 days of smoke had significantly more BALF cells with a trend toward increased macrophage (Figure 5, H and I) and neutrophil numbers (Supplemental Figure 4A).

MCL measurements of inflated lung sections from wild-type C57BL/6 mice exposed to chronic smoke exposure (8 months) displayed significantly increased lung airspaces compared with air-exposed controls, with no significant differences in lung area (Figure 5J and Supplemental Figure 4B). *Fasn*^iΔAEC2^ mice exposed to CS had significantly higher MCL values when compared with *Sftpc^CreERT2+/–^* mice exposed to CS (*P* = 0.007 by 1-way ANOVA). However, airspace enlargement was not observed in *Sftpc^CreERT2+/–^* control mice exposed to CS for 8 months, when compared to *Sftpc^CreERT2+/–^* room air controls, despite this being observed in C57BL/6 controls. Airspace enlargement trended (*P* = 0.07) higher in the CS-exposed *Fasn*^iΔAEC2^ mice compared with wild-type C57BL/6 mice and was significantly higher in the *Fasn*^iΔAEC2^ mice exposed to air, when compared with *Sftpc^CreERT2+/–^* mice (Figure 5J). The above trends remained upon adjusting for lung area. However, *Sftpc^CreERT2+/–^* mice exposed to smoke trended toward having higher adjusted MCL values when compared with *Sftpc^CreERT2+/–^* mice exposed to air (Supplemental Figure 4, B and C).

Finally, the percentage of TUNEL stain in the CS-exposed *Fasn*^iΔAEC2^ mice was not different from *Sftpc^CreERT2+/–^* mice exposed to CS (Figure 5K). However, *Fasn*^iΔAEC2^ mice had lower levels of pro-SPC (SFTPC) and higher levels of p53 at baseline and in response to smoke when compared with *Sftpc^CreERT2+/–^* controls (Figure 5L and Supplemental Figure 4F). MLE-12 cells, an AEC2 cell line transfected with *siFasn* and exposed in vitro to CS extract, displayed significantly increased apoptosis as assessed by FACS analysis using annexin V and 7-AAD (Supplemental Figure 4, D and E). The above data suggest that loss of FASN in AEC2 cells exacerbates smoke-induced injury in the lung.

### Targeted deletion of FASN in AEC2 cells alters the lipidomic response of the lung to smoke.

**Figure 6:**
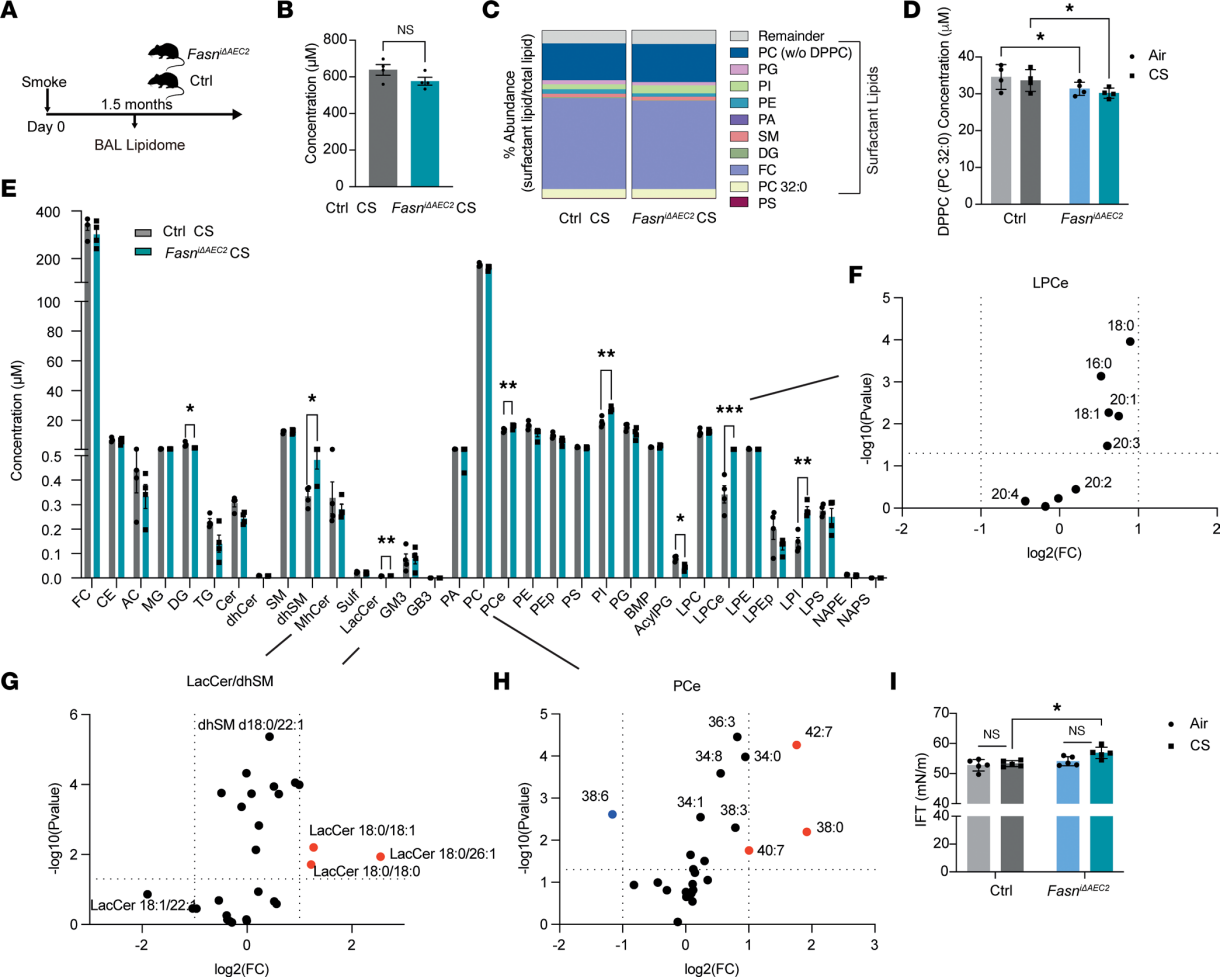
Targeted deletion of FASN in AEC2 cells alters the BALF lipidome and surface tension of the lung in response to smoke. (**A**) Schematic of BALF lipidomic profiling of *Fasn*^iΔAEC2^ and control *Sftpc^CreERT2+/–^* mice exposed to 6 weeks of smoke or room air. (**B**) Total lipid levels, (**C**) abundance (expressed as each surfactant lipid as a percentage of total lipids), (**D**) DPPC levels, (**E**) families of lipids and (**F** and **G**) individual LPCe species (**F**) or individual (**G**) dhSM and LacCer species (**H**) PCe in *Fasn*^iΔAEC2^ and control *Sftpc^CreERT2+/–^* mice exposed to 6 weeks of smoke (*n* = 4 per group). Red dots denote *P* < 0.05 fold-change of +2; blue dots denote *P* < 0.05 fold-change of –2. (**I**) Interfacial activity of the murine BALF in *Fasn*^iΔAEC2^ and control *Sftpc^CreERT2+/–^* mice exposed to 6 weeks of smoke (*n* = 5 per group) determined utilizing a sessile drop tensiometer. Data represented as mean ± SEM of 1 independent experiment. *P < 0.05, **P < 0.01, ***P < 0.001, by 1-way ANOVA followed by Tukey’s correction.

We next assessed whether a loss of FASN in AEC2 cells, which exacerbated smoke-induced injury in the lung, induced alterations in the levels of specific sphingolipids or phospholipid species important for surfactant composition. We performed lipidomic profiling in the BALF of *Fasn*^iΔAEC2^ and *Sftpc^CreERT2+/–^* controls exposed to acute CS for 6 weeks (Figure 6A). We chose to carry out this profiling on BALF to assess the lipid milieu of the alveolus and its association to our findings on the surfactant composition in the lung microenvironment. Overall, the absolute levels of total lipid levels in the *Fasn*^iΔAEC2^ mice exposed to CS trended toward being lower in the BALF when compared with control mice exposed to CS (Figure 6B and Supplemental Figure 5A). Similarly, the abundance (each lipid as a percentage/ratio of total lipids) of lipids important for surfactant composition trended toward being lower in the BALF from smoke-exposed *Fasn*^iΔAEC2^ mice when compared with smoke-exposed controls. In addition, levels of DPPC were significantly lower in the *Fasn*^iΔAEC2^ mice when compared with controls (Figure 6, C and D).

Upon smoke exposure, several families of lipids were decreased in the BALF of *Fasn*^iΔAEC2^ mice when compared with the *Sftpc^CreERT2+/–^* control mice. BALF from *Fasn*^iΔAEC2^ mice exposed to CS trended toward having lower levels of ACs, TGs, and PSs. Diacylglycerol (DG) and acyl PG classes showed significantly lower levels in *Fasn*^iΔAEC2^ when compared with *Sftpc^CreERT2+/–^* control mice (Figure 6E and Supplemental Figure 5, B and C). In the case of TGs, we found that 8 out of 42 TG species were significantly lower in BALF of the smoke-exposed *Fasn*^iΔAEC2^ mice when compared with CS-exposed *Sftpc^CreERT2+/–^* mice. Several PA, PC, PE, PS, PG, and BMP species were also significantly lower in the BALF of smoke-exposed *Fasn*^iΔAEC2^ mice when compared with CS-exposed *Sftpc^CreERT2+/–^* mice (Supplemental Figure 6A). In the absence of smoke, BALF from *Fasn*^iΔAEC2^ mice overall had significantly lower levels of ACs, DGs, Cers, dhSMs, monohexosylceramides (MhCers), lactosylceramides (LacCers), PEps, PIs, lysophosphatidylserines (LPSs), and acyl PGs, when compared with *Sftpc^CreERT2+/–^* mice (Supplemental Figure 5A and Supplemental Figure 6B).

Significant increases in the levels of etherPLs PCe and LPCe, as well as increases in dhSM, LacCer, PI, and LPI groups, were all observed in the BALF of *Fasn*^iΔAEC2^ mice exposed to CS when compared with the BALF of *Sftpc^CreERT2+/–^* mice exposed to CS (Figure 6, E–H, and Supplemental Figure 6A). Analysis of whole lung tissue after bronchoalveolar lavage revealed that the above lipidomic changes were specific to the BALF, with only several LPI species showing significantly higher levels in the whole lung tissue of smoke-exposed *Fasn*^iΔAEC2^ mice when compared with CS-exposed *Sftpc^CreERT2+/–^* mice (Supplemental Figure 7, A–C).

Surfactant is a complex lipid and protein mixture that lines the alveolus to reduce surface tension. Low surface tensions are a requirement for effective gaseous exchange in the lung by preventing alveolar collapse. Efficient surfactant function requires low surface tension without any increases in surface viscosity, a function that is highly dependent on levels of DPPC (43). The above data show that a loss of FASN in AEC2 cells resulted in lower levels of DPPC and alterations in other phospholipid species, which may in turn increase the surface tension of surfactant. To assess if such changes in lipid species in the BALF affected the biophysical properties of surfactant, we assessed the surface tension of BALF from *Sftpc^CreERT2+/–^* and *Fasn*^iΔAEC2^ mice exposed to room air or CS for 6 weeks. Using a sessile drop optical tensiometer (44, 45), we found that *Fasn*^iΔAEC2^ mice had a significantly (*P* < 0.05) higher surface tension in response to smoke compared with *Sftpc^CreERT2+/–^* mice exposed to smoke (Figure 6I).

Collectively, the above data suggest that in response to smoke *Fasn*^iΔAEC2^ mice have increased abundance of etherPLs, dhMS, and LacCer species; lower abundance of acyl PGs and TGs; and lower levels of phospholipids related to surfactant composition (PCs including DPPC, PGs, DGs), all of which may contribute to the exacerbated responses of *Fasn*^iΔAEC2^ mice to smoke, including an increase in surface tension.

### Targeted deletion of FASN in AEC2 cells increases BALF PUFAs and protective markers of ferroptosis in the lung.

**Figure 7:**
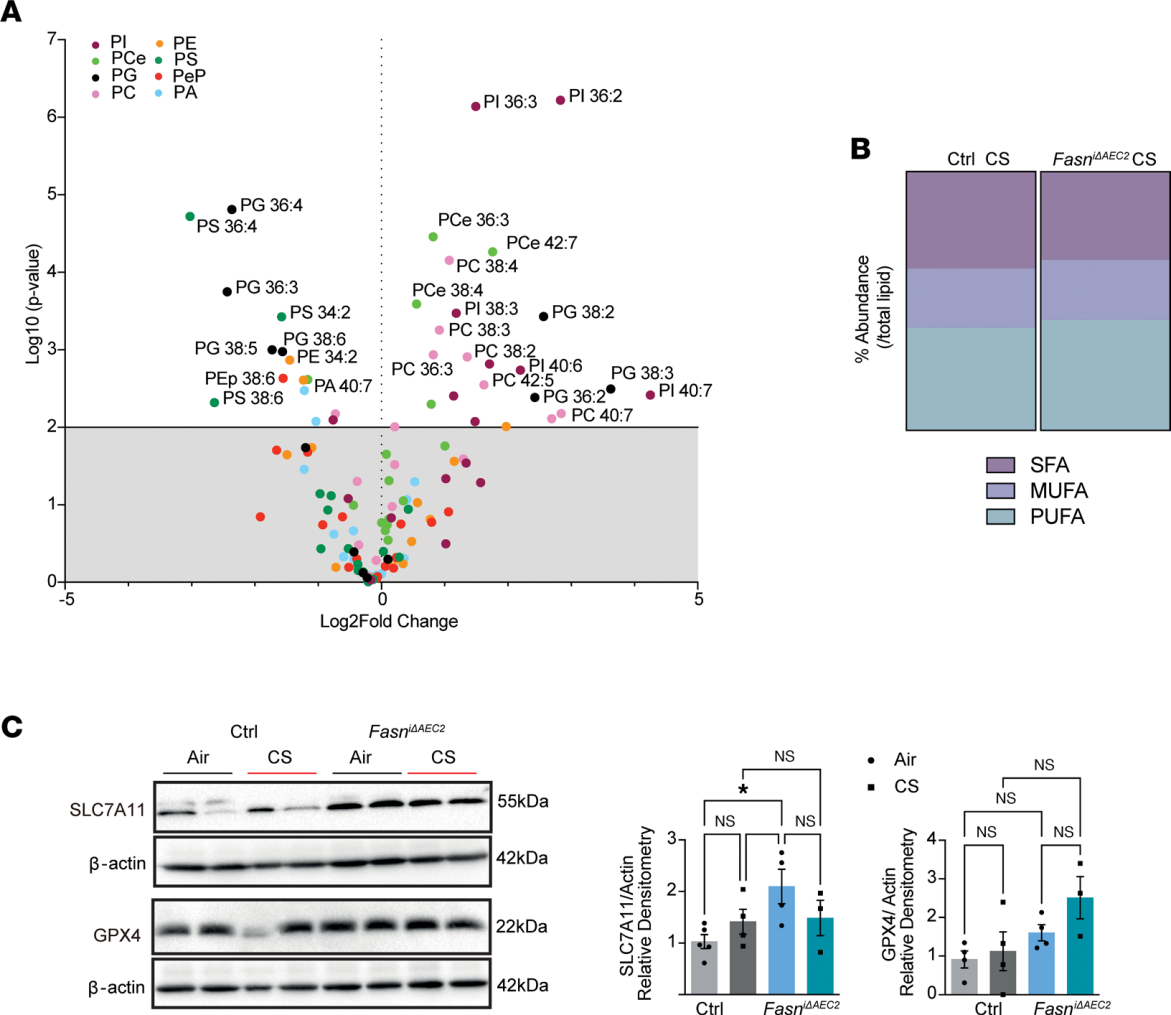
Targeted deletion of FASN in AEC2 cells increases abundance of PUFA-bound lipids and increases markers of ferroptosis in response to smoke. (**A**) Volcano plot of significantly altered PUFA-containing phospholipids in the *Fasn*^iΔAEC2^ mice compared with *Sftpc^CreERT2+/–^* control mice in response to 6 weeks of smoke (*n* = 4 per group). (**B**) Percentage abundance of saturated fatty acid–containing (SFA-containing), MUFA-containing, and PUFA-containing phosphatidylcholine species (calculated by expressing the concentration of each SFA, MUFA, and PUFA as a percentage of total phosphatidylcholine species). (**C**) Representative immunoblots (*left*) and relative quantification (*right*) for the ferroptosis markers, SLC7A11 and GPX4, with corresponding β-actin loading controls in *Fasn*^iΔAEC2^ mice compared with *Sftpc^CreERT2+/–^* control mice in response to 6 months of smoke. Immunoblot representative of *n* = 2 mice per group; densitometry representative of *n* = 3–5 mice per group. Data represented as mean ± SEM of 1 independent experiment. *P < 0.05 by 1-way ANOVA followed by Tukey’s correction.

EtherPLs are abundant in cell membranes and have been implicated in the regulation of differentiation, cell signaling, and responses to oxidative stress (30, 46–48). Peroxisomal enzymes (including fatty acyl coenzyme A reductase 1 or FAR-1) involved in the synthesis of etherPLs sensitize cells to ferroptosis, a form of lipid- and iron-associated cell death (49, 50). In cellular membranes, PUFAs bound to phospholipids or other lipid classes are preferred peroxidation substrates for ferroptosis (51). In this study, we show that the levels of PUFA-containing lipids were higher in AEC2 cells exposed to smoke (Figure 1E), and *Far-1* was significantly increased in the whole lung of *Fasn*^iΔAEC2^ mice when compared with *Sftpc^CreERT2+/–^* mice (log2FC 0.25, *P* = 0.012, Figure 3I). Consistently, levels of several PUFA-containing phospholipid species were significantly higher in the BALF of *Fasn*^iΔAEC2^ mice exposed to CS when compared with *Sftpc^CreERT2+/–^* mice exposed to CS, with an overall increased abundance in all PUFA-containing PC species (42.72% vs. 39.61%, *P* = 0.01 by 2-way ANOVA) (Figure 7, A and B).

Ferroptosis has been observed in the lung epithelium in human and experimental COPD (52). We therefore next wished to determine whether protective ferroptosis strategies are activated in the *Fasn*^iΔAEC2^ mice in response to increased levels of PUFA-containing phospholipids. We found that the expression of 2 key proteins that protect from lipid peroxides, glutathione peroxidase 4 (*Gpx4*), which reduces lipid peroxides to harmless alcohols using glutathione, and *Slc7a11* (also known as system Xc^–^ or xCT), the glutamate/cystine antiporter essential for glutathione synthesis, was increased in the *Fasn*^iΔAEC2^ mice (Figure 7C). Taken together the above data suggest that the increased ferroptosis-protective mechanisms in the lung may occur in response to increased PUFA lipids in the *Fasn*^iΔAEC2^ mice in response to smoke.

## Discussion

In this study, we show that exposure to CS reconfigures the lipid profile of AEC2 cells in vivo, reducing the abundance of surfactant-related lipids and increasing the concentration of etherPLs, TGs, and SM species. We uncover a critical function for the de novo lipogenesis enzyme FASN in AEC2 cell homeostasis in lipid reconfiguration in response to smoke exposure. We propose that in the absence of FASN, AEC2 cells cannot adapt their lipidome in response to smoke, rendering the AEC2 cell and consequently the lung more susceptible to inflammation and injury.

These hypotheses are supported by our findings that FASN is highly expressed in human and murine AEC2 cells and is decreased in murine and human AEC2 cells in COPD by scRNA-Seq analyses. Mice with a targeted deletion of FASN in AEC2 cells are more susceptible to smoke-induced injury and inflammation. In the absence of smoke, a loss of FASN in AEC2 cells also renders the lung more susceptible to age-induced increased airspace enlargement, which we attempted to restore by supplementing lipids in the diet. Although these findings were inconclusive, our results show a trend toward a restoration of the alveolar structure, which warrants future mechanistic studies into the role of FASN and lipid reconfiguration in COPD pathobiology.

In this study, FASN expression decreased in whole lung homogenates of mice exposed to smoke for 6 months in agreement with murine and human AEC2 single-cell data. However, FASN increased in isolated AEC2 cells from mice exposed to 6 months of CS. These conflicting observations could be attributed to a number of factors, including a loss in total AEC2 cell numbers in the lung after smoke (reflected by lower SFTPC levels), with those remaining viable AEC2 cells demonstrating increased adaptive FASN expression, or a loss of FASN in other cell types including fibroblasts, endothelial cells, and infiltrating macrophages, all of which play a role in COPD pathogenesis (4, 53).

We note that increased cholesterol synthesis and reduced PC levels increase susceptibility to emphysema (17, 54–56). However, this is the first study to our knowledge to interrogate and target lipid homeostasis in the AEC2 cell upon smoke. Our findings contribute to our understanding of AEC2 lipid metabolism in the lung, as well as the role of FASN in AEC2 cells under conditions of COPD development. A number of studies have described disrupted lipid metabolism in the BALF and whole lung tissue from smokers and individuals with COPD (Supplemental Table 2) (11, 12, 57), as well as in experimental emphysema in mice (11, 58, 59), supporting the contribution of AEC2 cells to changes in pulmonary surfactant and lipid homeostasis. Here, our murine data showed similarities between lipid alterations in human BALF or sputum, including lower BALF PC species such as DPPC, lower BALF PG species, higher BALF etherPLs, lower BALF BMPs, as well as higher BALF Cers and SMs (Supplemental Table 2). In this study we provide insight as to the mechanism for these observations, namely that FASN-associated lipogenesis most likely occurs as an adaptation to maintain correct surfactant synthesis or to generate pro-resolving lipid mediators in the lung upon smoking. This in turn results in an altered surfactant phospholipidome that may contribute to COPD pathogenesis.

Others have shown that FASN is required for surfactant production of alveolar epithelial cells and thus essential to normal lung function (20–23). FASN may also be utilized to generate FA substrates needed for FA oxidation (60), which in turn is essential in AEC2 cell function (61) and the development of fully differentiated airway epithelial cells (62). Moreover, the progression of emphysema pathology is associated with a loss in l-carnitine, critical for FA oxidation (63). Nevertheless, and to the best of our knowledge, this is the first study to show that FASN, particularly in AEC2 cells, is essential for the regulation of lipid homeostasis in the lung in response to smoke. Others have shown that FASN is essential to the induction of senescence in murine liver (64) and to AEC2 cell proliferation (65). Whether changes in FASN play a role in the senescent phenotypes observed in the COPD lung or the proliferation and/or regeneration of AEC2 cell remains to be determined.

As mentioned, efficient surfactant function requires low surface tension without any increases in surface viscosity, a function that is highly dependent on levels of DPPC (43). In this study, loss of FASN in AEC2 cells results in lower levels of DPPC in the BALF, and subsequent increases in surface tension in BALF upon smoke exposure, which could result in damage to the alveolar epithelium. Besides DPPC, mammalian surfactant contains other PC species, with PGs, PEs, SMs, PIs, and PSs comprising the remainder of the phospholipid pool (26). In this study, and in agreement with data on human tissues, several PG, PA, PC, PE, and PS species were lower in the *Fasn*^iΔAEC2^ mice exposed to smoke, which may drive the increased surface tension observed in the BALF of *Fasn*^iΔAEC2^ mice exposed to smoke.

In this study, we also show that at the time points studied here, CS increases the abundance of several lipids, including Cers, SM species, etherPLs (plasmalogens), and TGs, in the AEC2 cell and in the BALF of *Fasn*^iΔAEC2^ mice. Levels of Cer species are increased in the sputum of smokers with COPD, and loss of sphingomyelin synthase 2 is linked to the development of CS-induced COPD (12, 31). Consistently, we observed that CS resulted in increased levels of several Cer and SM species in AEC2 cells, while ablation of FASN in AEC2 cells boosted the concentration of a number of dhSM and LacCer species after smoke.

Our observed increases in etherPLs in both the AEC2 cells and *Fasn*^iΔAEC2^ mice exposed to smoke may be a response to the loss of the more abundant phospholipid species present in surfactant or may be reflective of the adaptations in lipid homeostasis in response to hypoxia (66). While plasmalogens, the most common form of etherPLs, are a minor component of surfactant, higher levels of plasmalogens reduce the surface tension and viscosity of surfactant (67, 68). Lower levels of etherPLs in the BALF of individuals with COPD are associated with a lower forced expiratory volume in 1 second in these people (11), suggesting that higher etherPLs may be protective in COPD. However, recent studies have shown that the peroxisomal enzymes involved in the synthesis of etherPLs sensitize cells to ferroptosis, a form of lipid- and iron-associated cell death that has been observed in the lung epithelium in human and experimental COPD (49, 50, 52). Interestingly, lipid oxidation also affects pulmonary surfactant homeostasis (69, 70), and ferroptosis sensitivity is determined by PUFA-containing phospholipids, which are subject to peroxidation (51). Here, we show that smoke exposure increases the abundance of PUFA-containing phospholipids, in BALF samples from *Fasn*^iΔAEC2^ mice. In agreement, lung tissues from *Fasn*^iΔAEC2^ mice show increased levels of protective ferroptosis markers.

Alternative interpretations to our findings must consider that alterations in extracellular BALF surfactant levels may be an indirect effect of inefficient AEC2 membrane remodeling (in response to the loss of FASN), which in turn may alter surfactant secretion by exocytosis in response to smoke (71). Similarly, there are numerous mechanisms of smoke-mediated disruption of surfactant metabolism, including inhibition of DPPC synthesis (72), reverse lipid transport (73, 74), or enhanced phospholipase A_2_ activity (75). Finally, changes in surfactant levels in the BALF may also be reflective of increased lipid recycling by alveolar macrophages (AMs) (76). FASN is downregulated in the AMs of patients with COPD, and these AMs are enriched in PCs and CEs, which suggests that in mild-severity stages, AMs may contribute to the decrease in surfactant lipids in the alveolar space by increasing their surfactant catabolism rate (53).

These studies may provide a basis for exploring the possible use of surfactant replacement therapy in COPD. Others have shown that surfactant replacement therapy provides pulmonary function improvement in individuals with bronchitis, a common component of COPD (77). However, the mechanism for this improvement, as well as the roles of specific individual surfactant lipids in COPD, remain unclear. Understanding the role of each BALF lipid and the interplay between the AEC2 cell and the AM in terms of lipid handling in COPD must be thoroughly investigated before therapeutically targeting lipid metabolism in COPD.

Taken together, these data suggest that FASN may be essential for the adaptation of the AEC2 cell lipidome to smoke. Specifically, both an increase in lipid synthesis and FASN levels may represent one indispensable mechanism engaged by the AEC2 cell to maintain surfactant production and membrane integrity in response to smoke. In conclusion, we demonstrate AEC2 cell lipid dysregulation as a key component of increased airspace size and identify FASN as a potential mechanistic target in COPD.

## Methods

### Generation of conditional AEC2-specific FASN-knockout mice.

To generate the AEC2-specific *Fasn*^iΔAEC2^ mice, *Fasn^loxP/loxP^* homozygous mice were crossed with *Sftpc^CreERT2+/+^* mice (see Supplemental Methods) to generate *Fasn^loxP/loxP^* *Sftpc^CreERT2+/–^* mice (*Fasn*^iΔAEC2^). *Fasn^–/–^* *Sftpc^CreERT2+/–^* littermates served as controls. All animals were housed in a pathogen-free environment in humidity and temperature-controlled rooms on a 12-hour light/12-hour dark cycle. Mice were given food and water freely. Tamoxifen (T5648, MilliporeSigma) was prepared as a 20 mg/mL stock solution in sunflower seed oil (S5007, MilliporeSigma). Mice were injected once a day for 3 days (100 mg/kg), followed by a 3-day rest period, then injected again for another 3 days at 5 weeks of age. Age- and sex-matched mice were used for experiments after 8 weeks of age.

### CS exposure of mice.

Eight- to 12-week-old *Fasn*^iΔAEC2^ mice or their control *Fasn^–/–^* *Sftpc^CreERT2+/–^* littermate mice were age- and sex-matched and chosen at random and exposed to CS in modular chambers as previously described (28, 29). Briefly, using a TE-10 inhalation exposure apparatus (Teague Enterprises), mice were exposed to CS (100 3R4F cigarettes, University of Kentucky Center for Tobacco Reference Products) with an average total particulate matter of 150 mg/m^3^ for 2 hours per day, 5 days per week, for 1.5–8 months. For C75 and smoke treatments, C75 was administered via I.P. injection using the following dosing regimen: 1 mg/kg for days 1–7, 2.5 mg/kg for days 7–13, 5 mg/kg for days 14–22, then 10 mg/kg for days 23–42 throughout smoke exposures for a total of 42 days. Murine lungs were inflated using 4% paraformaldehyde (Electron Microscopy Sciences) and kept at a pressure of 25 cmH_2_O for 20 minutes for alveolar MCL measurements as outlined in the Supplemental Methods and as previously described (40).

### Isolation of AEC2 cells.

Murine AEC2 cells were isolated as previously described and as outlined in the Supplemental Methods (78). Briefly, the lungs were perfused with PBS and inflated with dispase (BD Biosciences, 354235) followed by 0.5 mL of 1% low-melting-point agarose (Life Technologies, 16520-050). The lung tissue was digested in dispase, mechanically dissected, and filtered. AEC2 cells were enriched using MACS technology (Miltenyi Biotec) as described in the Supplemental Methods.

### Electrospray ionization-mass spectrometry of lipid molecular species.

All samples were collected and treated following recently accepted guidelines for lipidomics analysis (79). Lipids were extracted from murine lung tissue, BALF, or isolated primary AEC2 cells from equal amounts of material (100 μg/sample) by a chloroform-methanol extraction method and lipid extracts analyzed as described in the Supplemental Methods using a 6490 Triple Quadrupole LC/MS system (Agilent Technologies) spiked with appropriate internal standards.

### Determination of surface and interfacial activity of the murine pulmonary surfactant.

The surface and interfacial activity of the mouse pulmonary surfactants was determined using the commercial DataPhysics OCA 25 instrument, as described previously (80). Briefly, 27 μL of murine BALF was dispensed into a droplet from the nozzle. The density (kg/m^3^) of the liquid was measured and inputted and the surface tension of the droplet measured in zero field γ0. The surface tension (IFT mN/m) was then calculated by determining the shape of the droplet, with a change in droplet shape equating to a change in surface tension (80). Each measurement was repeated for a minimum of 4 droplets.

### RNA-Seq.

RNA was extracted from mouse lung tissue using TRIzol reagent (Invitrogen) and purified with the RNeasy Plus Mini Kit (QIAGEN). RNA-Seq libraries were prepared with the TruSeq Stranded mRNA Library Preparation kit (Illumina), according to manufacturer’s instructions. The cBot fluidic device (Illumina) was used to hybridize samples onto a flow cell and to generate clonal clusters of the DNA fragments. The sequencing was performed on the HiSeq 4000 sequencer (Illumina) and analyzed as described in the Supplemental Methods. scRNA-Seq data obtained from 15 control rejected donor lungs and 17 lungs from patients with advanced COPD/emphysema undergoing transplant (National Center for Biotechnology Information Gene Expression Omnibus [NCBI GEO] accession number GSE136831) as previously described (34) were reanalyzed as outlined in the Supplemental Methods.

### FASN activity.

FASN enzyme activity was determined as previously described (81). Briefly, 20 μL of fresh whole lung homogenate (1 μg protein/μL) was added to 70 μL of assay buffer (0.14 M potassium phosphate buffer pH 7.0, 1.4 mM EDTA pH 8.0, 1.4 mM DTT, 0.24 mM NADPH, 0.1 mM acetyl-CoA). The rate of NADPH oxidation was monitored at 340 nm at baseline and again after adding 10 μL of 0.85 mg/mL malonyl-CoA (MilliporeSigma). The substrate-dependent rate was determined by subtracting the baseline NADPH oxidation rate from the rate after addition of malonyl-CoA.

### Statistics.

All data were expressed as the mean ± SEM. Box plots show the interquartile range (box), median (line), and minimum and maximum (whiskers). Pairs of groups of samples distributed parametrically were compared by unpaired, 2-tailed Student’s *t* test. One-way ANOVA followed by Tukey’s correction was used for multigroup comparisons. Significance was accepted at *P* < 0.05. Data analysis was performed by Prism version 9 (GraphPad Software).

### Study approval.

All the animal protocols used in the study were approved by the Institutional Animal Care and Use Committee of Weill Cornell Medical College (New York, New York, USA) (protocol 2014-0020). Human samples obtained from Lung Tissue Research Consortium used in Figure 2 were tissues obtained from open lung biopsies described previously (28).

### Data availability.

Supporting data for all values underlying data presented in graphs are available in the Supporting Data Values file. RNA-Seq data have been deposited to NCBI GEO (accession number GSE235644).

## Author contributions

LCF, SMC, JFX, and AMKC conceived and designed the study. LCF, KMC, KK, SK, JAQ, YC, and NCW performed the experiments. SMC, KPC, and MP provided technical support for the murine COPD model, FASN mice, and high-fat diet experiment. LCF, MP, EAG, SK, MS, and SMC performed the data analyses, and JFX, SMC, KPC, MEM, EAG, and AMKC provided critical discussions for data interpretation. LCF and SMC wrote the manuscript, and all coauthors reviewed and approved the final manuscript.

## Supplementary Material

Supplemental data

Supplemental data -- unedited gels

Supporting data values

## Version 1 08/22/2023

Electronic publication
