# Supplementary material for: Alveolar type II epithelial cell FASN maintains lipid homeostasis in experimental COPD
Source: JCI Insight. 2023 Aug 22;8(16):e163403. doi: 10.1172/jci.insight.163403 (PMC10543729; doi:10.1172/jci.insight.163403)
Supplement: Supplemental data [file jciinsight-8-163403-s032.pdf]

Supplemental Material

Alveolar type II epithelial cell FASN maintains lipid homeostasis in experimental COPD

Li-Chao Fan[1,2], Keith Mc Conn[1], Maria Plataki[1,3], Sarah Kenny[4], Niamh C. Williams[4], Kihwan Kim[1], Jennifer A. Quirke[5], Yan Chen[2], Maor Sauler[6], Matthias E. Möbius[5], Kuei-Pin Chung[1,7,8], Estela Area Gomez[9,10], Augustine M.K. Choi[1,3], Jin-Fu Xu[2*], and Suzanne M. Cloonan[1,4*]

[1]Division of Pulmonary and Critical Care Medicine, Joan and Sanford I. Weill Department of Medicine, Weill Cornell Medicine, New York, USA. [2]Department of Respiratory and Critical Care Medicine, Shanghai Pulmonary Hospital, School of Medicine, Tongji University, Shanghai, China. [3]New York-Presbyterian Hospital Weill Cornell Medicine, New York, USA. [4] School of Medicine, Trinity Biomedical Sciences Institute, Trinity College Dublin, Ireland. [5] School of Physics, Trinity College Dublin, Ireland, [6] Pulmonary, Critical Care and Sleep Medicine, Yale School of Medicine, New Haven, CT, USA. [7]Department of Laboratory Medicine, College of Medicine, National Taiwan University, Taipei, Taiwan. [8] Department of Laboratory Medicine, National Taiwan University Hospital, Taipei, Taiwan[9]. Department of Neurology, Division of Neuromuscular Medicine, Columbia University Irving Medical Center Neurological Institute, New York, USA. [10]Centro de Investigaciones biologicas "Margarita Salas", CSIC, Madrid, Spain.

Methods

Generation of conditional AEC2-specific FASN knockout mice

Fasn$^{loxp/loxp}$ mice (*Fasn* locus with LoxP-flanked exons 4-8) were from Dr. Clay F. Semenkovich (Washington University, St Louis, MO, USA). *Sftpc*$^{CreERT2+/+}$ mice were from Dr. Brigid Hogan (Duke Medicine, Durham, NC). To generate the AEC2-specific *Fasn*$^{i\Delta AEC2}$ mice, *Fasn*$^{loxp/loxp}$ homozygous mice were crossed with *Sftpc*$^{CreERT2+/+}$ mice to generate *Fasn*$^{loxp/loxp}$ *Sftpc*$^{CreERT2+/-}$ mice (*Fasn*$^{i\Delta AEC2}$). *Fasn*$^{-/-}$ *Sftpc*$^{CreERT2+/-}$ littermates served as controls. The number of backcrosses of mice was less than ten. FASN-specific primers used for genotyping by PCR were previously described (1). Genotyping for *Sftpc*$^{CreERT2+/+}$ DNA was performed by PCR using the forward primer 5'-GCT TCA CAG GGT CGG TAG-3' and the reverse primer 5'-CAA CTC ACA ACG TGG CAC TG-3'. All animals were housed in pathogen-free environment in humidity and temperature-controlled rooms on a 12-12-h light-dark cycle. Mice were given food and water freely. Tamoxifen (T5648, Sigma-Aldrich) was prepared as a 20 mg/ml stock solution in sunflower seed oil (S5007, Sigma-Aldrich). Mice were injected once a day for 3 days (100 mg/kg), followed by a 3-day rest period, and then injected again for another 3 days at 5 weeks of age. Age and sex-matched mice were used for experiments after 8 weeks of age.

Bronchoalveolar lavage cell differentials

Mice were euthanized by CO2 narcosis, the tracheas cannulated and the lungs lavaged with 1 ml increments of ice-cold PBS. BALF was centrifuged at 1500 x rpm for 5 minutes. 1 ml red

blood cell lysis buffer (Sigma Aldrich) was added to the cell pellet and left on ice for 15 minutes followed by centrifugation at 1500 x rpm for 5 minutes. The cell pellet was resuspended in 1 ml PBS and leukocytes were counted using a hemocytometer. Specifically, 20 µl was removed for cell counting performed in triplicate using a hemocytometer and 200 µl removed for cytocentrifuge preparations (Shandon Cytospin3, 350 rpm for 3 minutes) and stained using the Hema3 staining system (Fisher Scientific). The percentage of macrophages, lymphocytes and polymorphonuclear leukocytes (PMNs) were counted in a total of 300 cells, and absolute numbers of each leukocyte subset were calculated. BALF protein concentration was determined by Pierce BCA Protein Assay Kit (Thermo Scientific Pierce, #23227).

Isolation of alveolar type II epithelial cells

Murine AEC2 cells were isolated as previously described and as outlined in the Supplement (2). Briefly, the lungs were perfused with PBS to remove blood and inflated with dispase (BD Biosciences, #354235). To avoid leakage of dispase, 0.5 ml of 1% low melting point agarose (Life Technologies, #16520-050) was instilled. The lung tissue was transferred to a 50 ml Corning tube containing 2 ml dispase for 45 min at room temperature. The lung tissue was then put in 10 ml of DMEM with 25 mM HEPES buffer (Invitrogen, #15630-080), 100 U/ml of Penicillin-Streptomycin (ThermoFisher Scientific, #15140122) in a 10-mm culture plate and mechanically dissected. The cell suspension was filtered through 100-, 40- and 20-µm Falcon™ cell strainers (Thermo Fisher Scientific). The cell pellets were collected by centrifugation at 200 x *g* for 10 min. The supernatant was discarded and resuspended in MACS separation buffer (2 mM EDTA, 0.5% MACS® BSA Stock solution in PBS) with mouse CD45

microbeads (Miltenyi Biotec, #130-052-301) at 4°C for 15 min. The cells were washed with MACS separation buffer and transferred to LS columns (Miltenyi Biotec, #130-042-401). The CD45(-) cells were separated by MidiMACS Separator (Miltenyi Biotec, #130-042-302) and incubated with FcR blocking reagent (Miltenyi Biotec, #130-092-575) for 10 min at 4°C. The cells were then incubated with anti-mouse CD326 (EpCAM) (eBiosciences, #13-5791-82) Biotin at 4°C. After 30 min, cells were washed and incubated with Streptavidin microbeads (Miltenyi Biotec, #130-048-102) for 15 min. The CD45(-)EpCAM(+) cells were isolated with a MidiMACS Separator. The isolated AEC2 cells were identified by immunofluorescence staining using anti-SPC (Santa Cruz, #Sc-7706).

Western blotting

The lungs were homogenized in RIPA extraction buffer (ThermoFisher Scientific, #89900) and centrifuged at 13,000 rpm for 15 min at 4°C. The supernatant was mixed with LDS sample buffer (Invitrogen, #1691979) boiled for 10 minutes. Proteins were separated by NuPAGE 3%-8% or 4%-12% Bis-Tris gels and transferred onto 0.45 μm PVDF transfer membrane (Millipore, #IPVH00010) by electroblotting. Membranes were probed with the following antibodies; FASN (1:1000, Cell Signaling, #3180), ACC (1:1000, Cell Signaling Technology, #3676), ACLY(1:1000, Cell Signaling, #4332), GAPDH(1:2000, Cell Signaling #5174),anti pro-surfactant protein C (1:1000, Abcam, #AB211326) , anti p53 (1:1000, Cell Signalling, #2524S), ferroportin (1:100, MTP1/IREG1/Fpn Generon, #MTP11-A), GPX4 (1:1000 Abcam, #52455) and SLC7A11 (1:1000, Abcam, #12691), β-actin (1:5000-1:10000, Sigma-Aldrich,

#A2228). Peroxidase-AffiniPure Goat Anti-Rabbit IgG (H+L) (1:2000, Jackson Immunoresearch, #111-035-003) or peroxidase-AffiniPure Goat Anti-Mouse IgG (H+L) (1:2000, Jackson Immunoresearch, 115-035-003) bands were detected using the Chemidoc XPS system (BioRad) after incubation with ECL prime Reagent (ThermoFisher Scientific). Band density was determined using Image J software (National Institutes of Health). In some cases, actin was run on the same membrane as other proteins, but in other cases it was run on a sperate gel using the same volume and concentration of samples at the same time as the other gels. We have made it clear in the figure legends when this occurred.

Lung histochemistry and Immunofluorescence

Mice lungs were fixed with 25 cm of 4% paraformaldehyde (PFA, Electron Microscopy Sciences, #15710) in PBS for 15 min. Isolated lungs were immersed in 4% PFA overnight, immersed in 75% alcohol and sent to the Pathology core lab at Weill Cornell Medicines (WCM) for tissue processing, paraffin embedding and IHC staining. Briefly, 5-µm paraffin tissue sections were deparaffinized, rehydrated, inactivation of endogenous peroxidase, and incubation with the different primary antibody (FASN, 1:500, cell signaling, #3180; Pro-SPC, 1:500, Abcam, #ab90716) overnight at 4 °C. Then the sections incubated with secondary antibody, polymer, diaminobezidine, and hematoxylin. For TUNEL stain, sections were pre-incubated in solution of 30 mM Trizma Base, 140 mM Sodium Cacodylate pH7.2 and 1mM Cobalt Chloride for 15mins before aspirating and applying the TUNEL reaction mixture [0.1M Soduim Cacodylate pH7, 0.1mM DTT, 0.05 mg/ml Bovine Serum Albumin, 2.0 u/ µl Terminal

Transferase (Sigma, #03333566001), 0.2 nmoles Biotin-16-dUTP (Sigma, # 11093070910), 2.5mM Cobalt Chloride] for 1 hour in a humidified chamber at 37°C. The reaction was terminated by transferring slides to a bath of 300mM Sodium Chloride and 30mM Sodium Citrate for 15 mins. at room temperature. After washing 3 times with PBS slides were blocked with 2% serum albumin (human) in PBS for 10 min. in a humid chamber, washed three times and then incubated with Avidin-Biotin Complex Elite (Vector Labs PK-61000) diluted 1:25 in PBS for 30 mins. Slides were then stained with 3,3'-Diaminobenzidine (Fisher, #H54000). TUNEL stain was quantified using threshold analysis in Image J after removal of red blood cells. Briefly background intensity of stain was calculated for all nuclei/cells, followed by intensity of TUNEL specific stain. The intensity of TUNEL stain was then expressed as percentage of total stain.

Human lung tissues were obtained from patients at autopsy and tissue samples were fixed in formalin and embedded in paraffin. For Immunofluorescence on paraffin-embedded tissue sections, the samples were incubated with primary antibodies (FASN, 1:100, cell signaling, #3180; Pro-SPC, 1:100, Santa Cruz, #sc-7706) in blocking buffer overnight at 4 °C. The samples were washed 3 times with PBS and probed with fluorescently labeled secondary antibodies. Alexa Fluor 488– and Alexa Fluor 633–conjugated secondary antibodies are both from Thermo Fisher. The nucleus was labeled with Hoechst 33258(1:2000, life science, #H3569), photographed with a confocal microscope (Zeiss LSM 880 laser scanning microscope).

Mean chord length measurement

Murine lungs were inflated using 4% paraformaldehyde (PFA) (Electron Microscopy Sciences) and kept at a pressure of 25 cmH_2O for 20 minutes. The lungs were then dissected and subsequently fixed in 4% PFA at 4°C for 24 hours. After fixation, the samples were transferred to tissue cassettes (Tissue-Tek), immersed in 70% ethanol at 4°C, and sent to the core lab at Weill Cornell Medicines (WCM) Pathology and Laboratory Medicine for tissue processing and paraffin embedding. Parasagittal tissue sections with thickness of 5μm was then used for modified Gill's staining as previously described (3). After blinding and randomization, we obtained at least 10 fields under 200X magnification for each sample, using EVOSTM FL Imaging System (Invitrogen). Alveolar mean chord length (MCL) for each sample was used to assess the enlargement of the airspace, and was measured based on the published protocol, with some modifications (3). Briefly, the images were first processed using Adobe Photoshop CS software (version 24.1.1), and the non-alveolar structures, including bronchi and vessels, were painted black. The MCL for each image was then measured using FIJI running Image J software (version 1.51w). Given that the MCL measurement can be affected by the orientation of the grid lines (4), both horizontal MCL (MCLH) and vertical MCL (MCLV) were measured through applying the horizontal and vertical grid lines (grid line spacing as 5 μm), respectively. The MCL for each sample was then determined by the average of MCLH and MCLV. Alveolar surface area is determined by both MCL and lung volume (5), with the latter subject to variability attributed to inflation. To assess any bias due to variable inflation, we calculated the

sample volume for each sample using the Cavalieri method (6), which uses the section thickness and section area to determine volume. Given that the section thickness was the same (5μm) for each sample, section area was thus used for correcting MCL data between samples and was measured using the whole-slide scan image, through FIJI running Image J software. Therefore, the MCL data between samples were presented both as unadjusted and adjusted using sample volumes/area.

Electrospray Ionization-Mass Spectrometry of Lipid Molecular Species

All samples were collected and treated following recently accepted guidelines for lipidomics analysis (7). Lipids were extracted from mouse lung tissue, BALF or isolated primary AEC2 cells from equal amounts of material (100 micrograms/ sample) by a chloroform–methanol extraction method as described in (8) in the core lab of lipidomics in Columbia University (9-11). Lipid extracts were analyzed using a 6490 Triple Quadrupole LC/MS system (Agilent Technologies) and were spiked with appropriate internal standards. Three comprehensive panels, scanning for either positive lipids, negative lipids or neutral lipids (under positive mode), were analysed for 51 samples. Equal amounts of internal standards with known concentrations were spiked into each extract. Each standard was later used to calculate the concentrations of corresponding lipid classes by first calculating ratio between measured intensities of a lipid species and that of corresponding internal standard multiplied by the known concentration of the internal standard. Glycerophospholipids and sphingolipids were separated with normal-phase HPLC as described before (11), with a few modifications. An

Agilent Zorbax Rx-Sil column (inner diameter 2.1 x 100 mm) was used under the following conditions: mobile phase A (chloroform:methanol:1 M ammonium hydroxide, 89.9:10:0.1, v/v) and mobile phase B (chloroform:methanol:water:ammonium hydroxide, 55:39.9:5:0.1, v/v); 95% A for 2 min, linear gradient to 30% A over 18 min and held for 3 min, and linear gradient to 95% A over 2 min and held for 6 min. Sterols and glycerolipids were separated with reverse-phase HPLC using an isocratic mobile phase as before(11) except with an Agilent Zorbax Eclipse XDB-C18 column (4.6 x 100 mm). Quantification of lipid species was accomplished using multiple reaction monitoring (MRM) transitions that were developed in earlier studies(11) in conjunction with referencing of appropriate internal standards: PA 14:0/14:0, PC 14:0/14:0, PE 14:0/14:0, PI 12:0/13:0, PS 14:0/14:0, SM d18:1/12:0, D7-cholesterol, CE 17:0, MG 17:0, 4ME 16:0 diether DG, D5-TG 16:0/18:0/16:0 (Avanti Polar Lipids, Alabaster, AL).

The data were first normalized using the NOMIS approach to reduce any systematic variabilities, such as batch effects, as described by Sysi-aho, et al and implemented in “metabolomics” R package. Then the normalized data were used to draw a PCA graph to confirm the removal of systematic variations as well as to detect any possible outliers that would be subject to biological interpretation. No clustering was observed indicating removal of systematic variabilities by normalization. Samples outside confidence level of 95% were removed from the data set for analysis. The data were used to calculate fold change and p-value for each comparison made between groups, and these statistics were used to draw the graphs presented. The premise has been that it is equally important to reject the null hypothesis (H0)

or the alternative hypothesis (H1) with confidence. Based on power analysis, we set the α-error (false-positive) ≤ 5% and β-error (false-negative) ≤ 20%. Differences among means were have ≥ 20% to reflect meaningful differences. The estimated biological replicates were sufficient to reach a statistical power ≥ 80%. All experiments showed power is < 80% and achieve a risk of false-positive < 5% or of false-negative < 20%. All data sets were tested for normality and equality of variance.

Murine RNA-seq Analysis

The raw sequencing reads in binary base call (BCL) format were processed and analyzed as described in the Supplement through bcl2fastq 2.19 (Illumina) for FASTQ format conversion and demultiplexing. RNA reads were aligned and mapped to the mouse reference genome by TopHat2 (version 2.0.11) (https://ccb.jhu.edu/software/tophat/index.shtml)(12) , and transcriptome reconstruction was performed by Cufflinks (version 2.1.1) (https://cole-trapnelllab.github.io/cufflinks/). The abundance of transcripts was measured with Cufflinks in Fragments Per Kilobase of exon model per Million mapped reads (FPKM) (13, 14). Gene expression profiles were constructed for differential expression, cluster, and principle component analyses with the DESeq2 package (https://bioconductor.org/packages/release/bioc/html/DESeq2.html) (15). For differential expression analysis, pairwise comparisons between two or more groups using parametric tests where read-counts follow a negative binomial distribution with a gene specific dispersion parameter. Adjusted *p*-values for multiple testing were calculated based on the Benjamini-

Hochberg method. Heat maps were plotted using Heatmap Illustrator software (Heml 1.0) (hemi.biocuckoo.org) (16) , based on the z scores calculated using the gene expressions by FPKM. All data has been deposited to GEO (GSE235644).

scRNASeq data analysis.

scRNAseq data obtained from 15 control rejected donor lungs and 17 lungs from subjects with advance COPD/emphysema undergoing transplant (GSE136831) along with their associated clinical characteristics were obtained as previously described (17). Briefly, there were eight females in both groups and the median age of all subjects was 62 years old (range 41–80). All COPD subjects had radiographic evidence of advanced emphysema and were former smokers, while four of the donors without COPD were either current or former smokers. Individuals with COPD had a mean ratio of forced expiratory volume in one second (FEV_1) to forced vital capacity (FVC) of 0.36 ± 0.05 and a mean FEV_1 percent predicted of 21.0 ± 5.0 (17). Methods for procuring samples, tissue digestion, barcoding, library preparation, and sequencing, have been previously described (17). We also reanalyzed scRNAseq data obtained from *Sftpc-CreER^{T2}-m^{T}m^{G}* mice exposed to cigarette smoke from 3R4F research cigarettes (University of Kentucky) or room air for 6 h of exposure per day, 5 days/week for 10 months in a Teague TE-10 smoking machine. Details regarding tamoxifen injection, cigarette smoke exposure, tissue digestion, barcoding, library preparation, and sequencing have also been previously described (17). Analyses were performed using Seurat (v4.0.6.).

Apoptosis assay

MLE-12, mouse lung epithelial type II cells were cultured in HITES (Hydrocortisone, Insulin, Transferrin, Estrogen) media [RPMI1640, 2% FBS, insulin (5 mg/mL), transferrin (10 mg/mL), sodium selenite (30 nM), hydrocortisone (10 nM), beta-estradiol (10 nM), HEPES (10 nM)]. Cells were grown to 80% confluence for experiments. For transient knockdown of FASN, MLE-12 cells were plated on 6-well plates at a density of 5 x 10^5 cells per well and were transfected with siRNA of mouse FASN or negative control (Dharmacon) using Lipofectamine RNAiMAX Reagent (Invitrogen, #13778-075) according to the manufacturer's instructions. CSE was prepared and added to culture media as previously described (18-21). Apoptosis of cells was measured by FITC Annexin V Apoptosis detection kit I (BD Pharmingen™, #556547, NY, USA) and flow cytometry (BD Accuri™ C6, NY, USA) according to the manufacturer's instructions.

RNA isolation and real-time qPCR

RNA was isolated from whole lung or AEC2 cells from mice using TRIzol (Invitrogen, #15596-018). RNA was treated with DNase at room temperature for 15 min before cDNA synthesis. RNA (2 μg) was reverse-transcribed and then analyzed by quantitative RT-PCR for using SYBR®Green PCR Master Mix (Applied Biosystems, #4309155). Real-time qPCR was carried out in the ABI PRISM 7500 Real-Time PCR System (Applied Biosystems). The fold-change of the target genes was calculated by using the $2^{-\Delta\Delta CT}$ method. A list of genes and associated primers are described in Supplemental Table 1 below.

Supplemental Table 1-Primers for Real-Time PCR (mouse)

Name	Forward	Reverse
Fasn	5’-gcc atc cag att gcc ctc at-3’	5’-ccc aag gag tgc cca atg at-3’
Scd1	5′-gtg agg cga gca act gac ta -3′	5′- ggg cac tgt ctt cac ctt ct -3′
Fdps1	5′-aag atc ctg atg gag atg gg -3′	5′-agc aga cac tga acc acc ag -3′
Fabp5	5′-ggg aag gag agc acg ata ac -3′	5′-tct cat aga ccc gag tgc ag -3′
Thrsp	5′-gcc gaa gaa gac agg atc tc -3′	5′-tca ggt ggg taa gga tgt ga -3′
β-actin	5’-tgg aat cct gtg gca tcc atg aaa-3’	5’-taa aac gca gct cag taa cag tcc g-3’

Supplemental Table 2- Lipid changes from human studies and how they relate to murine data presented in this study

Lipid	Changes in COPD BALF/Sputum	Clinical Relevance	Changes in murine AEC2 cells and/or BALF
Total Lipids	Lower in COPD BALF (22)	The concentration of total lipids in BALF correlated positively with FEV1(22)	Lower total lipids in BALF of *Fasn*$^{i\Delta AEC2}$ mice exposed to smoke
PUFA lipids	Significantly lower sputum levels of essential dietary PUFAs and PUFA-derived lipid mediators in stable COPD patients compared to controls (23)	During exacerbation, slight increases in dietary PUFA levels compared to stable COPD (23)	PUFA containing lipids higher in AEC2 cells isolated from mice exposed to 6 months smoke. PUFA-containing phospholipids higher in in BALF of *Fasn*$^{i\Delta AEC2}$ mice exposed to smoke
PC Lipids	Lower in COPD BALF (22)	PC lipids in BALF correlated positively with FEV1(22)	Lower PC lipids in BALF of *Fasn*$^{i\Delta AEC2}$ mice exposed to smoke correlating with worse injury
PC32:0, PC32:1, PC 34:1, PC 36:4,	Lower in COPD BALF (22)	Correlated with lower FEV1 (22)	Lower PC30:0, PC36:0, PC36:4, PC 38:6 in BALF of *Fasn*$^{i\Delta AEC2}$ mice exposed to smoke correlating with worse injury
PC(O-18:0_O-3:1) PC(18:2_18:2)		Associated with lower FEV1 and FEF25-75, + Exac study Yr 1, + Exac postbronch-Yr 1* (24)	PC(18:2/18:2) significantly lower in BALF of *Fasn*$^{i\Delta AEC2}$ mice exposed to smoke
PE lipids	Higher Pes, lysoPEs, acyl-PEs and plasmalogen PEs in sputum of smokers with COPD (25)	PE(P-38:3) associated with lower FEV1, lower FEF25-75, higher BDR (24)	PE 32:1, PE 34:2, PE 36:0, PE 36:4, PE 38:6, PE 42:5 significantly lower in the BALF of smoke exposed *Fasn*$^{i\Delta AEC2}$ mice when compared to CS exposed *Sftpc*$^{CreERT2+/-}$
PG Lipids	PG 36:2, significantly decreased in COPD BALF (22)	The concentration of PG in BALF correlated positively with FEV1(22)	Higher PG species (36:2, 38:1) in AEC2 cells from smoke exposed mice. PG30:0, PG 32:1, PG34:2, PG36:3, PG36:4, PG38:5, PG38:6 lower in in the BALF of smoke exposed *Fasn*$^{i\Delta AEC2}$ mice when compared to CS exposed *Sftpc*$^{CreERT2+/-}$
PS lipids		PS(O-36:0) associated with higher FEV1/FVC, higher FEV1, higher FEF25-75 (24) PS(42:7) associated with lower FEV1, lower FEV25-75, higher BDR	PS 36:0 significantly higher in KO BALF at baseline and in response to CS
Cholesterol	Total cholesterol Lower in COPD BALF (22)	The concentration of cholesterol in BALF correlated positively with FEV1(22)	The abundance of free cholesterol decreased in AEC2 cells isolated from smoke exposed mice
Ether Phospholipids	Ether PCs 30:0, 32:0, 38:5 increased in COPD BALF (22)	The concentration of ether-PC in BALF correlated positively with FEV1(22)	Ether PLs increased in AEC2 cells in response to smoke. Significant increases in the ether phospholipids PCe and LpCe, in the BALF of *Fasn*$^{i\Delta AEC2}$ mice exposed to CS when compared with the BALF of *Sftpc*$^{CreERT2+/-}$ mice exposed to CS
BMPs	BMP 36:1 and 36:2 lower in COPD BALF (22)	The concentration of BMPs in BALF correlated positively with FEV1(22)	BMP (BMP 30:0, BMP 32:1, BMP 34:0, BMP 34:1, BMP 34:2, BMP 36:3, BMP 36:4, BMP 38:5,

			BMP 38:6) species significantly lower in the BALF of smoke exposed *Fasn*$^{i\Delta AEC2}$ mice when compared to CS exposed *Sftpc*$^{CreERT2+/-}$
Diglycerides		DG(42:3) associated with higher BDR, higher CAT, + CB (24)	DG's had significantly lower levels in *Fasn*$^{i\Delta AEC2}$ mice when compared with *Sftpc*$^{CreERT2+/-}$ mice exposed to CS
Ceramides	28 ceramides higher in smokers with COPD sputum (25)	Higher expression in smokers with COPD and declines upon smoking cessation. Ceramides, significantly and inversely correlated with FEV1% predicted, FEV1/FVC, and residual volume/diffusion capacity for carbon monoxide corrected for hemoglobin % predicted in smokers with and without COPD (25)	Dihydroceramide (dhCer) 18:0/22:1 and ceramide d18:1/22:1 significantly higher in AEC2 cells isolated from smoke exposed mice. Cer d18:1/24:1, Cer d18:1/26:0 Cer d18:1/26:1, higher and Cer d18:1/22:0 Cer d18:1/24:0 lower in *Fasn*$^{i\Delta AEC2}$ mice when compared with *Sftpc*$^{CreERT2+/-}$ mice exposed to CS
Sphingomyelins	36 sphingomyelins higher in smokers with COPD sputum (25)<br><br>All species of SM were decreased in COPD patients, especially the most predominant SM, d18:1/16:0 in COPD BALF (22)	Higher expression in sputum of smokers with COPD (25)<br><br>The concentration of ether-PC in BALF correlated positively with FEV1(22)	sphingomyelin species SM d18:1/16:1, d18:1/20:0, and d18:1/20:1, dihydrosphingomyelin (dhSM) species d18:1/16:0, d18:1/18:1 and d21:1/24:1 significantly higher in AEC2 cells isolated from smoke exposed mice.<br>SM d18:1/24:1 SM d18:1/26:0 SM d18:1/26:1 lower in *Fasn*$^{i\Delta AEC2}$ mice when compared with *Sftpc*$^{CreERT2+/-}$ mice exposed to CS
Lysophospholipid		LysoPC(17:0) associated with lower FEV1 and lower BDR. LysoPC(22:0) associated with + Exac postbronch-Yr 1* LysoPE(22:6) associated with lower FEV1 (24)	LPC 18:1, LPC 20:2, LPI 18:0, LPI 18:1, LPI 20:4, LPS 20:1 higher and LPE 16:0, LPEp 16:0 lower in *Fasn*$^{i\Delta AEC2}$ mice when compared with *Sftpc*$^{CreERT2+/-}$ mice exposed to CS

*Exacerbations reported during SPIROMICS year 1 or post-bronchoscopy after one year; FEV1=forced expiratory volume in 1 sec; FVC=forced vital capacity; FEF25-75=maximum mid- expiratory flow; BDR=bronchodilator response; CAT=COPD Assessment Test; BDR= bronchodilator response; CB=chronic bronchitis.

References

1. Chakravarthy MV, Pan Z, Zhu Y, Tordjman K, Schneider JG, Coleman T, et al. "New" hepatic fat activates PPARalpha to maintain glucose, lipid, and cholesterol homeostasis. *Cell Metab.* 2005;1(5):309-22.
2. Messier EM, Mason RJ, and Kosmider B. Efficient and rapid isolation and purification of mouse alveolar type II epithelial cells. *Exp Lung Res.* 2012;38(7):363-73.
3. Laucho-Contreras ME, Taylor KL, Mahadeva R, Boukedes SS, and Owen CA. Automated measurement of pulmonary emphysema and small airway remodeling in cigarette smoke-exposed mice. *J Vis Exp.* 2015(95):52236.
4. Mitzner W, Fallica J, and Bishai J. Anisotropic nature of mouse lung parenchyma. *Ann Biomed Eng.* 2008;36(12):2111-20.
5. Dunnill MS. Quantitative methods in the study of pulmonary pathology. *Thorax.* 1962;17(4):320-28.
6. Michel RP, and Cruz-Orive LM. Application of the Cavalieri principle and vertical sections method to lung: estimation of volume and pleural surface area. *J Microsc.* 1988;150(Pt 2):117-36.
7. Burla B, Arita M, Arita M, Bendt AK, Cazenave-Gassiot A, Dennis EA, et al. MS-based lipidomics of human blood plasma: a community-initiated position paper to develop accepted guidelines. *J Lipid Res.* 2018;59(10):2001-17.
8. Guedes LC, Chan RB, Gomes MA, Conceicao VA, Machado RB, Soares T, et al. Serum lipid alterations in GBA-associated Parkinson's disease. *Parkinsonism Relat Disord.* 2017;44:58-65.
9. Gulati S, Ekland EH, Ruggles KV, Chan RB, Jayabalasingham B, Zhou B, et al. Profiling the Essential Nature of Lipid Metabolism in Asexual Blood and Gametocyte Stages of Plasmodium falciparum. *Cell Host Microbe.* 2015;18(3):371-81.
10. Byrdwell WC, Sato H, Schwarz AK, Borchman D, Yappert MC, and Tang D. 31P NMR quantification and monophasic solvent purification of human and bovine lens phospholipids. *Lipids.* 2002;37(11):1087-92.
11. Chan RB, Oliveira TG, Cortes EP, Honig LS, Duff KE, Small SA, et al. Comparative lipidomic analysis of mouse and human brain with Alzheimer disease. *J Biol Chem.* 2012;287(4):2678-88.
12. Kim D, Pertea G, Trapnell C, Pimentel H, Kelley R, and Salzberg SL. TopHat2: accurate alignment of transcriptomes in the presence of insertions, deletions and gene fusions. *Genome Biol.* 2013;14(4):R36.
13. Trapnell C, Hendrickson DG, Sauvageau M, Goff L, Rinn JL, and Pachter L. Differential analysis of gene regulation at transcript resolution with RNA-seq. *Nat Biotechnol.* 2013;31(1):46-53.
14. Trapnell C, Williams BA, Pertea G, Mortazavi A, Kwan G, van Baren MJ, et al. Transcript assembly and quantification by RNA-Seq reveals unannotated transcripts and isoform switching during cell differentiation. *Nat Biotechnol.* 2010;28(5):511-5.

15. Love MI, Huber W, and Anders S. Moderated estimation of fold change and dispersion for RNA-seq data with DESeq2. *Genome Biol.* 2014;15(12):550.
16. Deng W, Wang Y, Liu Z, Cheng H, and Xue Y. HemI: a toolkit for illustrating heatmaps. *PLoS One.* 2014;9(11):e111988.
17. Sauler M, McDonough JE, Adams TS, Kothapalli N, Barnthaler T, Werder RB, et al. Characterization of the COPD alveolar niche using single-cell RNA sequencing. *Nat Commun.* 2022;13(1):494.
18. Cloonan SM, Glass K, Laucho-Contreras ME, Bhashyam AR, Cervo M, Pabon MA, et al. Mitochondrial iron chelation ameliorates cigarette smoke-induced bronchitis and emphysema in mice. *Nat Med.* 2016;22(2):163-74.
19. Mizumura K, Cloonan SM, Nakahira K, Bhashyam AR, Cervo M, Kitada T, et al. Mitophagy-dependent necroptosis contributes to the pathogenesis of COPD. *J Clin Invest.* 2014;124(9):3987-4003.
20. Chen ZH, Lam HC, Jin Y, Kim HP, Cao J, Lee SJ, et al. Autophagy protein microtubule-associated protein 1 light chain-3B (LC3B) activates extrinsic apoptosis during cigarette smoke-induced emphysema. *Proc Natl Acad Sci U S A.* 2010;107(44):18880-5.
21. Chen ZH, Kim HP, Sciurba FC, Lee SJ, Feghali-Bostwick C, Stolz DB, et al. Egr-1 regulates autophagy in cigarette smoke-induced chronic obstructive pulmonary disease. *PLoS One.* 2008;3(10):e3316.
22. Agudelo CW, Kumley BK, Area-Gomez E, Xu Y, Dabo AJ, Geraghty P, et al. Decreased surfactant lipids correlate with lung function in chronic obstructive pulmonary disease (COPD). *PLoS One.* 2020;15(2):e0228279.
23. van der Does AM, Heijink M, Mayboroda OA, Persson LJ, Aanerud M, Bakke P, et al. Dynamic differences in dietary polyunsaturated fatty acid metabolism in sputum of COPD patients and controls. *Biochim Biophys Acta Mol Cell Biol Lipids.* 2019;1864(3):224-33.
24. Madapoosi SS, Cruickshank-Quinn C, Opron K, Erb-Downward JR, Begley LA, Li G, et al. Lung Microbiota and Metabolites Collectively Associate with Clinical Outcomes in Milder Stage Chronic Obstructive Pulmonary Disease. *Am J Respir Crit Care Med.* 2022;206(4):427-39.
25. Telenga ED, Hoffmann RF, Ruben tK, Hoonhorst SJ, Willemse BW, van Oosterhout AJ, et al. Untargeted lipidomic analysis in chronic obstructive pulmonary disease. Uncovering sphingolipids. *Am J Respir Crit Care Med.* 2014;190(2):155-64.

Supplemental Figure Legends

Supplemental Figure 1. (**A**) Representative confocal images of immunofluorescence analysis for CD45(-)EpCAM(+) cells (*left* panel) and quantification of the population (*right* panel) isolated from mice whole lung cell suspension performed by staining for DAPI- (blue) and pro-SPC (red). Data are expressed as Mean±SEM (n = 3 mice), (n=3 experiments). Scale bar, 200μm. (B) Volcano plot of significant lipid species in isolated AEC2 cells exposed to CS or Air (n=4 mice per group). Cholesterol Ester (CE), Lactosylceramide（LacCer）, Monosialodihexosylganglioside(GM3), Phosphatidic acids (PA), Phosphatidylinositols (PI), Monoacylglycerol(MG), Phosphatidylglycerol(PG), Phosphatylcholines(PC), Sphingomyelins(SM), Phosphatidylethanolamine (PE), Plasmogen Lysophosphatidylethanolamine (LPEp) species. Red dots denote $p<0.05$ fold change of +2.

Supplemental Figure 2. (**A**) UMAP plots of *FASN* expression (*right panel*) from previously published single-cell RNA sequencing (scRNASeq) profiles (*left panel*) of explanted lung tissue from subjects with advanced COPD or control lungs, which demonstrated that FASN is localized to AEC2 cells, specifically to a sub-population of surfactant producing AEC2 cells (AT2B). (**B**) UMAP plots of *Fasn* expression (*right panel*) from previously published single-cell RNA sequencing (scRNASeq) profiles (*left panel*) of mice exposed to 10 months smoke. (**C**) Representative IHC staining of FASN and pro-SPC in lung sections from C57BL/6 mice exposed to CS or Air condition for 6 months (n=3 per group), (n=3 experiments). Scale bars, 100μm. Magnification of inset is 8 times of original magnification. (**D**) Immunofluorescence of FASN and pro-SPC in lung tissue from C57BL/6 mice by confocal microscopy. Scale bar, 20μm.(**E**) Immunoblotting of FASN in different organs from C57 BL/6 mice. n=2 mice per group, GAPDH and β-actin were measured as an internal control. FASN, ACLY, β-actin and GAPDH were all run on different gels. For FASN 15μg/8ul sample was added to each lane, for β-actin and GAPDH 11.25 μg/6ul sample was added to each lane. (F) Immunoblotting for FASN, ACC, ACLY in lung tissue from C57 BL/6 mice expose to Air or CS for 6 months (*top panel*), n=6 mice per group with corresponding densitometry (*bottom panels*), (n=3 experiments). FASN and ACLY were immunoblotted on the same gel. ACC and β-actin were immunoblotted on the same gel. The same samples were used to load both gels. (**G**) *Fasn* gene expression in whole lung tissue from C57 BL/6 mice expose to Air or CS for 6 months measured by q-PCR (n=6 mice per group). Data are expressed as Mean±SEM of one independent experiment, $^{**}p<0.001$ by unpaired student t test.

Supplemental Figure 3. (A) Immunoblot with quantification of FASN, ACLY and ACC protein expression in primary isolated AEC2 cells from $Fasn^{i\Delta AEC2}$ and control $Sftpc^{CreERT2+/-}$ mice (n=6 per group), (n=3 experiments). FASN and ACLY were immunoblotted on the same gel. ACC and β-actin were immunoblotted on the same gel. The same samples were used to load both gels. (**B**) Quantitative RT-PCR was performed to assess the fatty acid metabolism related mRNA (*Fasn*, *Scd1*, *Fdps1*, *Fabp5* and *Thrsp*) in whole lung (*left panel*) sections (n=3 per group) and isolated AEC2 cells (*right panel*) from $Fasn^{i\Delta AEC2}$ and Ctrl mice (n=6 per

group), (n = 2 technical replicates). *p <0.05; **p<0.01; * * *p<0.001 by student's unpaired t-test. （**C**) Lipidomic analysis of total lipid concentration (μM) in lung tissue of *Fasn*$^{i\Delta AEC2}$ mice relative to control mice (n=4 per group). (D-E) Volcano plots of significantly altered species of (**D**) Bis(monoacylglycero)phosphate (BMP) and **(E)** Lysophosphatidylinositol (LPI) species in the *Fasn*$^{i\Delta AEC2}$ mice when compared to the control *Sftpc*$^{CreERT+/-}$ (Ctrl) mice. Red dots denote p<0.05 fold change of +2, blue dots denote p<0.05 fold change of -2. (F) Lung areas between Ctrl and *Fasn*$^{\mathrm{i\Delta AEC2}}$ mice (Ctrl, n=9; *Fasn*$^{\mathrm{i\Delta AEC2}}$, n=13). (**G**) Lung areas (left panel) and mean chord length (MCL) values adjusted to lung area (*right panel*) in murine lung sections of *Fasn*$^{\mathrm{i\Delta AEC2}}$ and control *Sftpc*$^{\mathrm{CreERT2+/-}}$ mice at 18 months upon supplementation with a high fat (60%) or low fat (10%) diet (Ctrl, low fat n=13; *Fasn*$^{\mathrm{i\Delta AEC2}}$, low fat n=8; Ctrl, high fat n=10; *Fasn*$^{\mathrm{i\Delta AEC2}}$ high fat n=9). Data represented as Mean ± SEM of one independent experiment. *p < 0.05, **p < 0.01, ***p < 0.001 by student's unpaired t-test.

Supplemental Figure 4. (A)Total BAL neutrophils total BAL macrophages of mice administered C75 (1mg/kg 1-7 days, 2.5 mg/kg days 7-13, 5mg/kg days 14-22, then 10mg/kg days 23-42, DMSO as control) for a total of 42 days exposure of cigarette smoke. (RA DMSO, n=5; DMSO CS, n= 4; RA C75, n=5; C75 CS n=5). (**B**) Lung area (Ctrl Air, n=8; Ctrl CS, n=10; *Fasn*$^{\mathrm{i\Delta AEC2}}$ Air, n=7; *Fasn*$^{\mathrm{i\Delta AEC2}}$ CS, n=13) and (**C**) mean chord length (MCL) values adjusted to lung area in murine lung sections of mouse lungs for each group (Ctrl Air, n=8; Ctrl CS, n=10; *Fasn*$^{\mathrm{i\Delta AEC2}}$ Air, n=8; *Fasn*$^{\mathrm{i\Delta AEC2}}$ CS, n=13) calculated from *Fasn*$^{i\Delta AEC2}$ and *Sftpc*$^{Cre\mathrm{ERT2+/-}}$ control mice, as well as C57BL/6 controls exposed to 8 months of CS exposure. (**D**) Immunoblot analysis for FASN and GAPDH in cell lysates from MLE-12 cells transduced with siCtrl and si*Fasn* (n=3 experiments). FASN and GAPDH were immunoblotted on the same membrane/gel. (**E**) Representative flow panel of annexin V and 7-AAD apoptosis test of siCtrl and si*Fasn* MLE-12 cells with or without 20% cigarette smoke extract (CSE) for 12 hours (n=3 experiments). (**F**) Immunoblot analysis of p53, pro-SFTPC and β-actin in whole lung homogenates of mice exposed to Air or smoke for 6 months with corresponding densitometry in the fold change of pro-SFTPC (*bottom*) (Ctrl Air, n=3; Ctrl CS, n=3; *Fasn*$^{\mathrm{i\Delta AEC2}}$ Air, n=4; *Fasn*$^{\mathrm{i\Delta AEC2}}$ CS, n=3); immunoblot representative of n=2 mice per group. Both p53 and pro-SFTPC had their own actin loading controls on the same gel. Data represented as Mean ± SEM of one independent experiment, ns: not significant.

Supplemental Figure 5. (**A**) Lipidomic profiling of BALF lipid extracts from *Fasn*$^{\mathrm{i\Delta AEC2}}$ and control *Sftpc*$^{CreERT2+/-}$ (Ctrl) mice exposed to 6 weeks of cigarette smoke（CS） or room air (Air). (**B-C**) Volcano plots of significant DG and Acyl PG species between *Fasn*$^{\mathrm{i\Delta AEC2}}$ and control mice exposed to cigarette smoke (CS) for 6 weeks (n=4 per group).

Supplemental Figure 6. (**A**) Heat map of BALF lipid extracts of individual species (A) and lipid classes (**B**) from *Fasn*$^{\mathrm{i\Delta AEC2}}$ and control Ctrl mice exposed to 6 weeks of CS or Air (n=4 per group). Yellow and blue indicate increased and decreased levels, respectively. Rows: lipid species; Columns: mice BALF sample.

Supplemental Figure 7. (A-C) Lipidomic analysis of (**A**) total lipid concentration (µmol/mg protein), (**B**) lipid classes as well as (**C**) volcano plot of significant LPI species in the whole lung tissue of *Fasn*$^{i\Delta AEC2}$ mice relative to control mice upon 6 weeks smoke exposure (n=4 per group). FC, free cholesterol; CE, cholesterol ester; DG, diacylglycerols; TG, triacylglycerols; Cer, ceramides; SM, sphingomyelins; PA, phosphatidic acids; PC, phosphatylcholines; PCe, phosphatidylcholine ethers; PE, phosphatidylethanolamines; PEp, plasmalogen phosphatidylethanolamines; PS, phosphatidylserines; PI, phosphatidylinositols; PG,phosphatidylglycerols; LPC,lysophosphatidylcholines; LPE, lysophosphatidylethanolamines. Red dots denote $p<0.05$ fold change of +2, black dots denote $p<0.05$ fold change of -2.

A

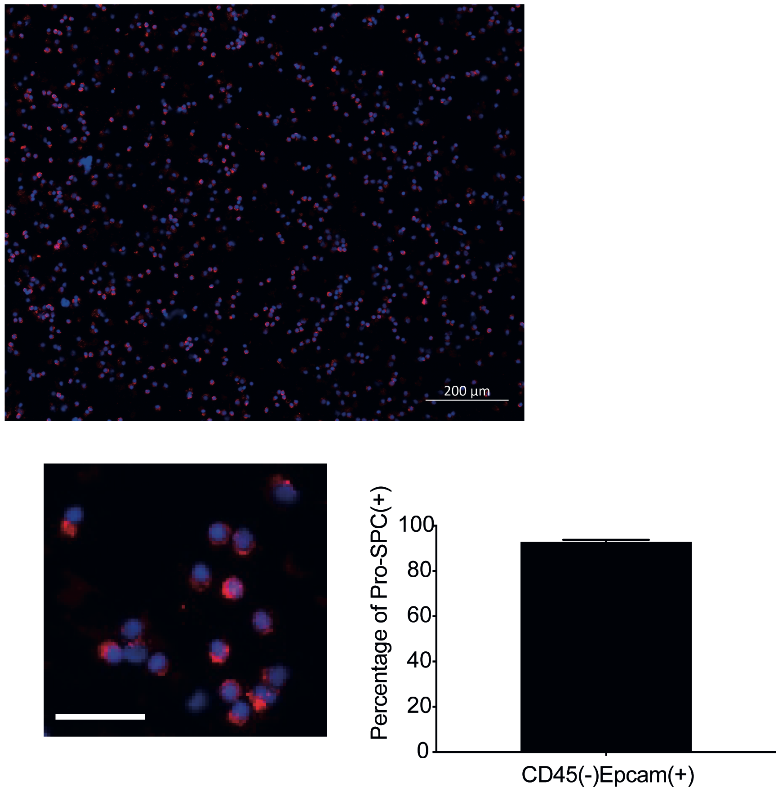

B

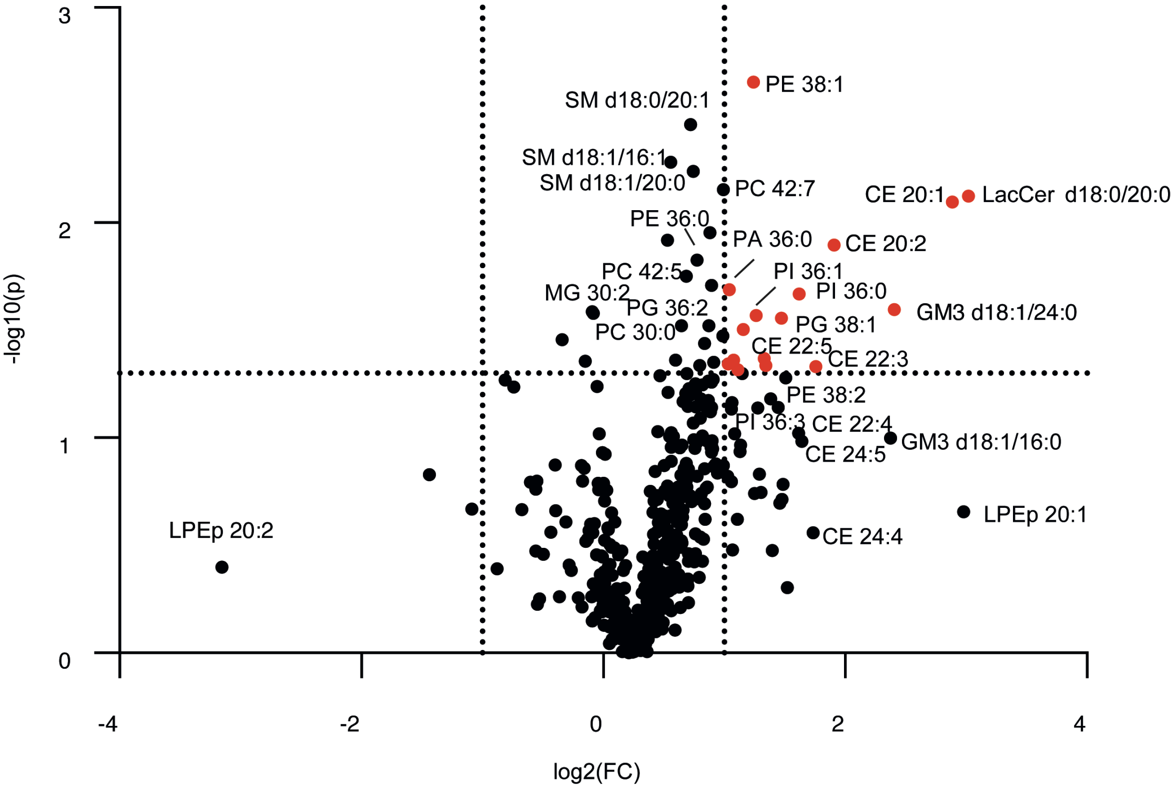

Supplemental Figure 1

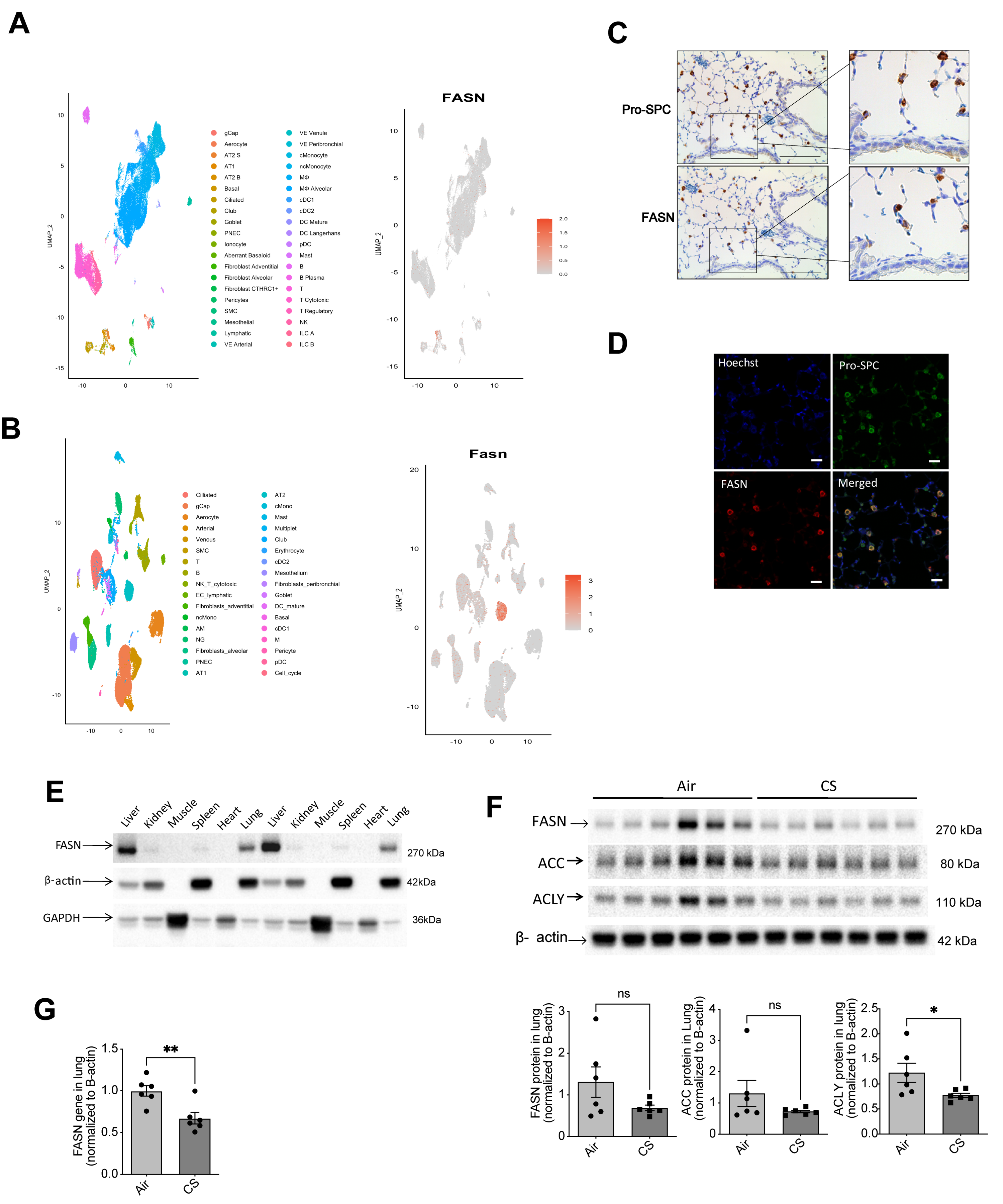

Supplemental Figure 2

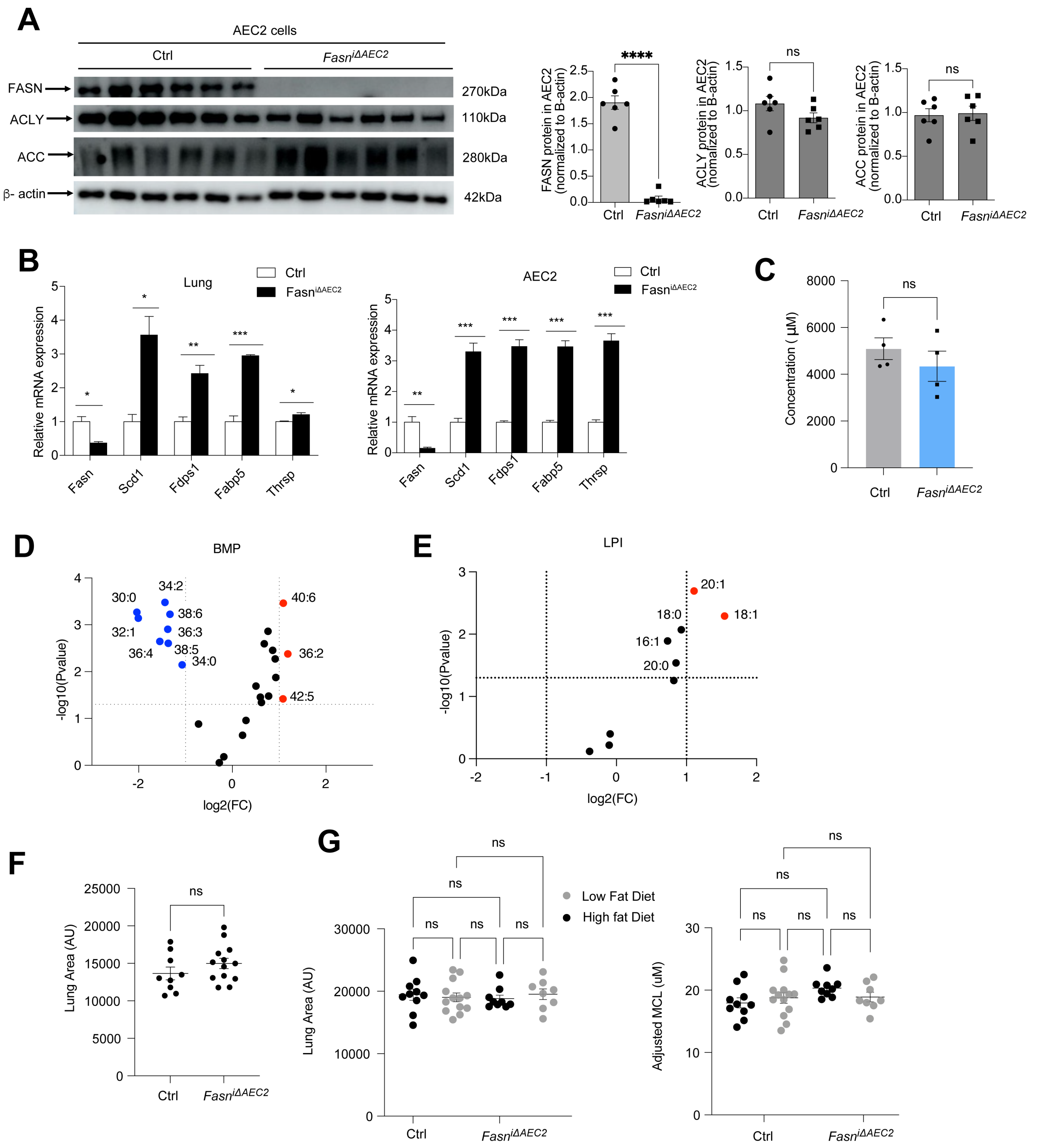

Supplemental Figure 3

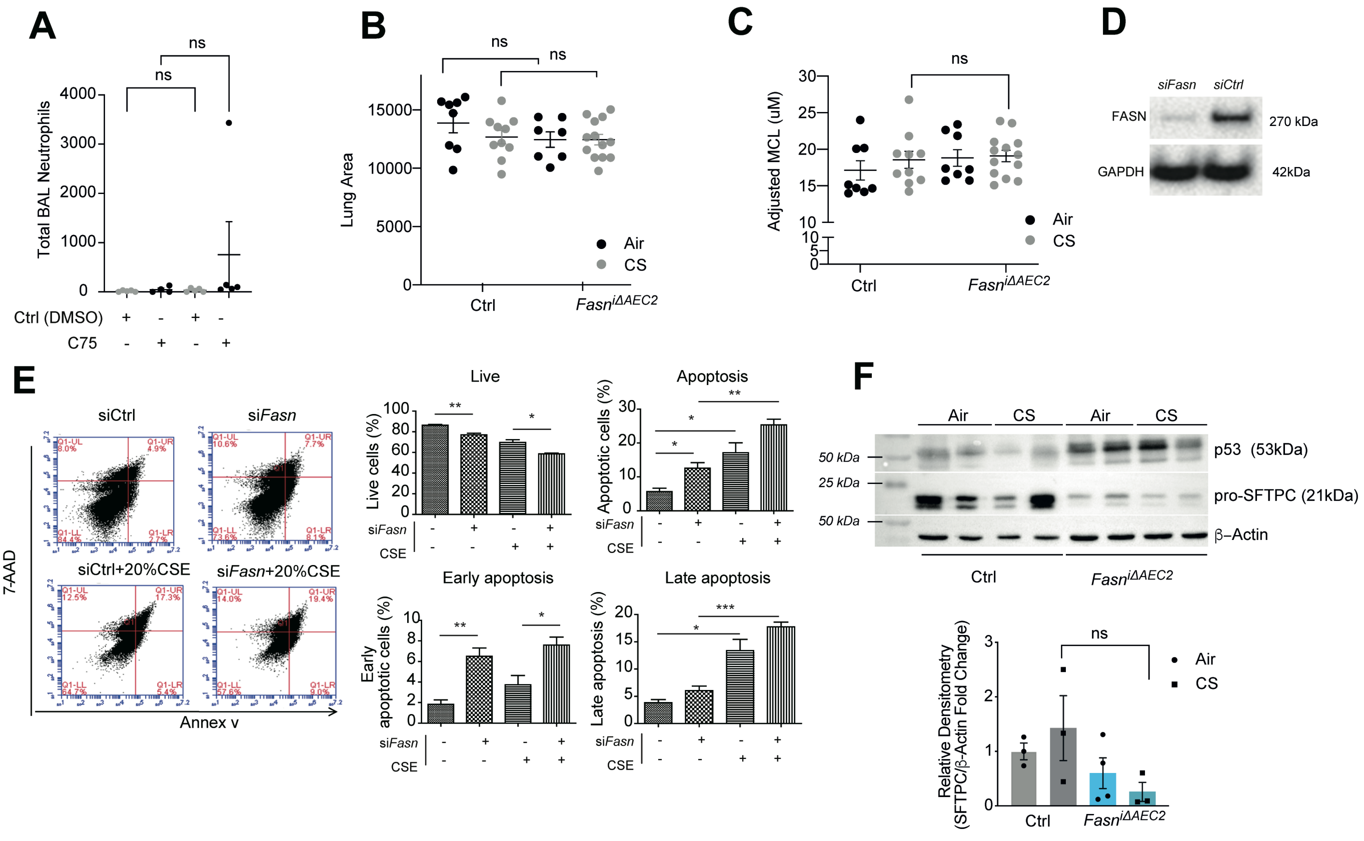

Supplementary Figure 4

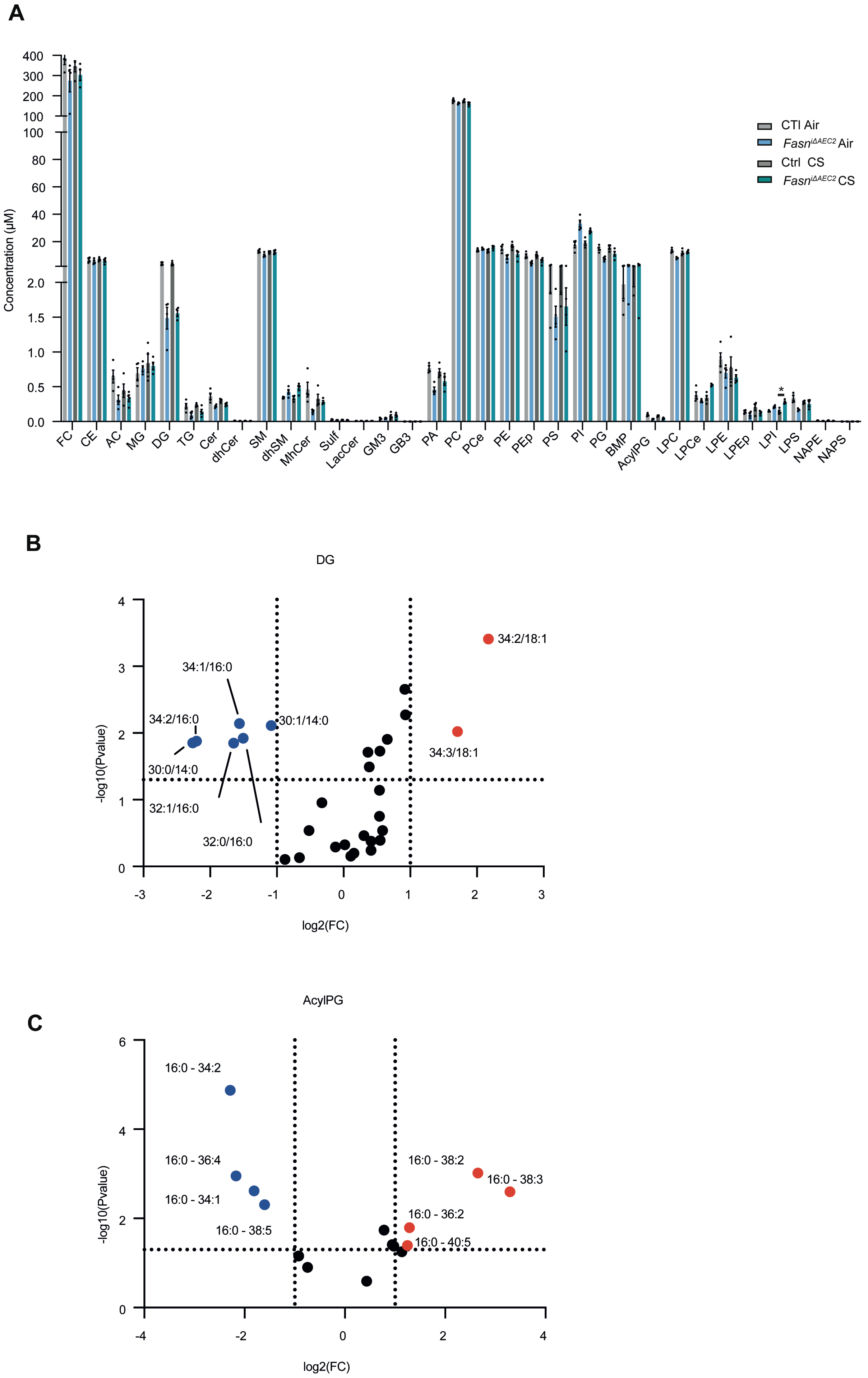

Supplementary Figure 5

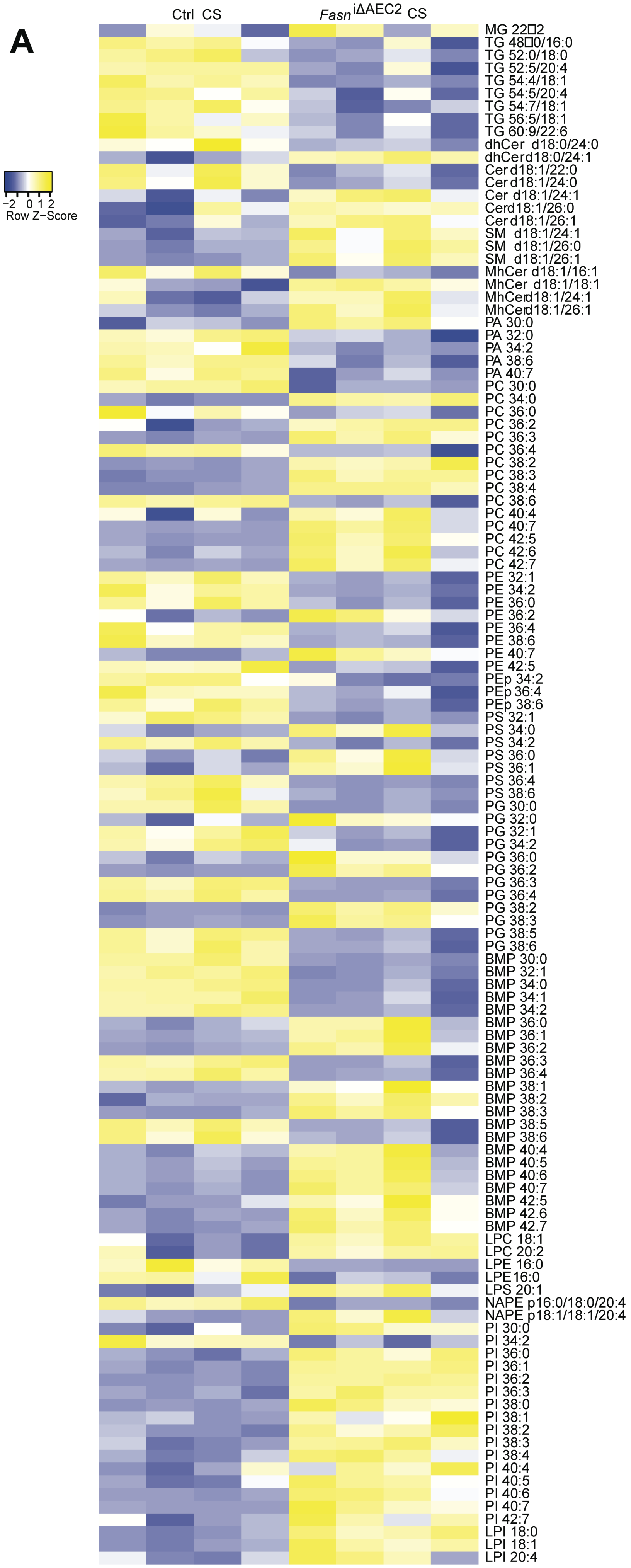

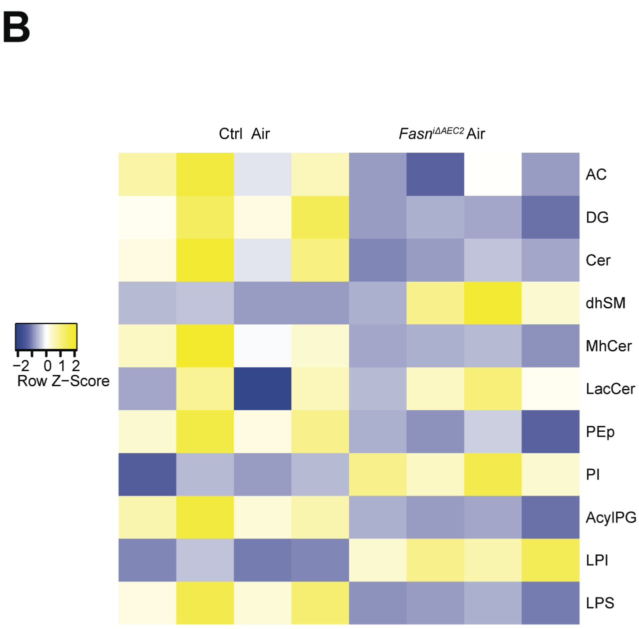

Supplementary Figure 6

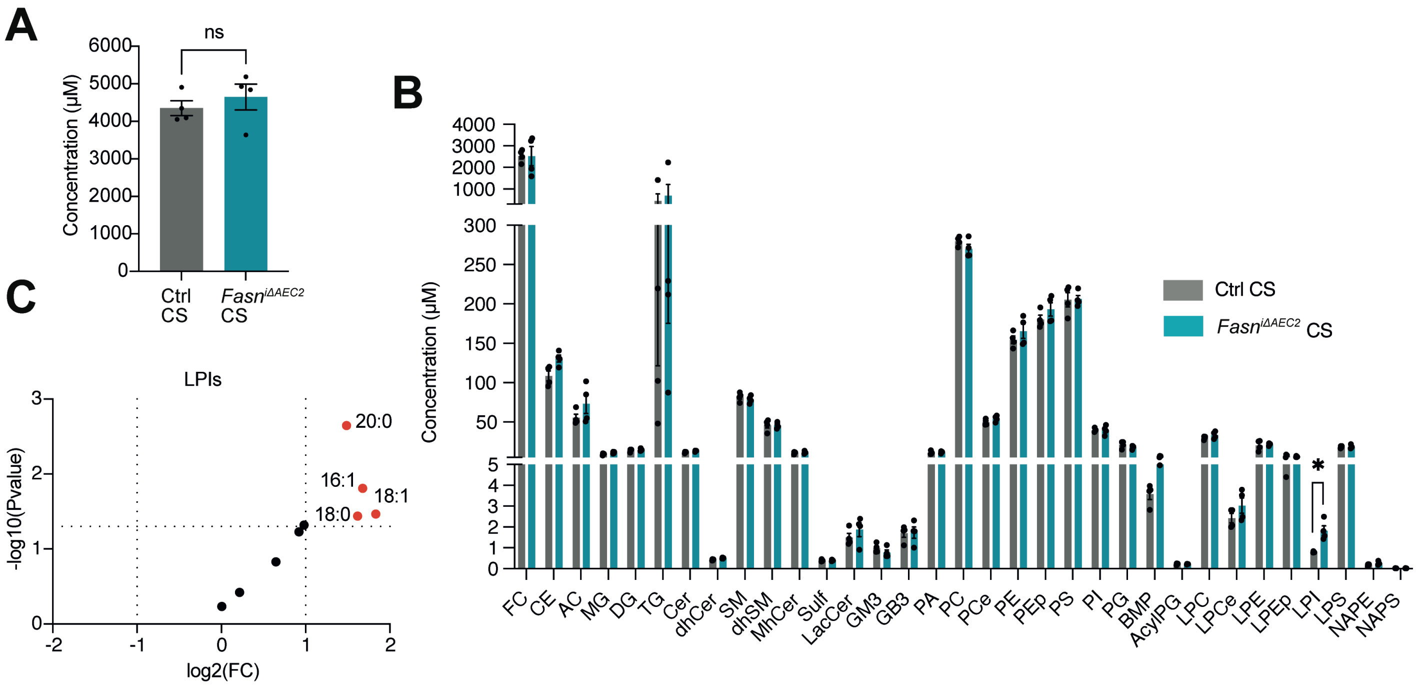

Supplementary Figure 7
