# Supplementary material for: Alveolar type II epithelial cell FASN maintains lipid homeostasis in experimental COPD
Source: JCI Insight. 2023 Aug 22;8(16):e163403. doi: 10.1172/jci.insight.163403 (PMC10543729; doi:10.1172/jci.insight.163403)
Supplement: Supplemental data -- unedited gels [file jciinsight-8-163403-s033.pdf]

# Figure 2E

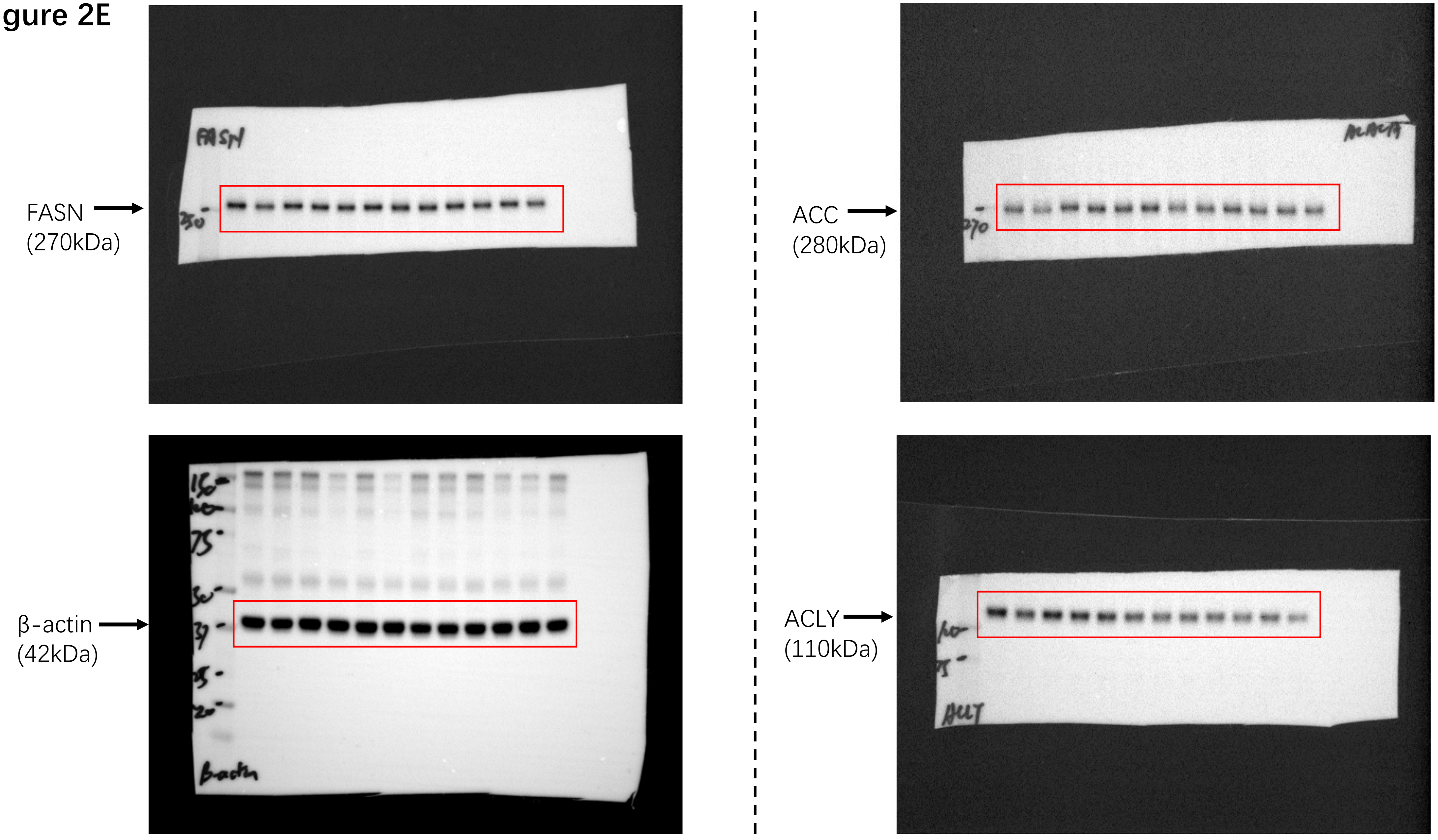


FASN and β-actin were from the same membrane.

ACC and ACLY were from the same membrane.

**Figure 2G**

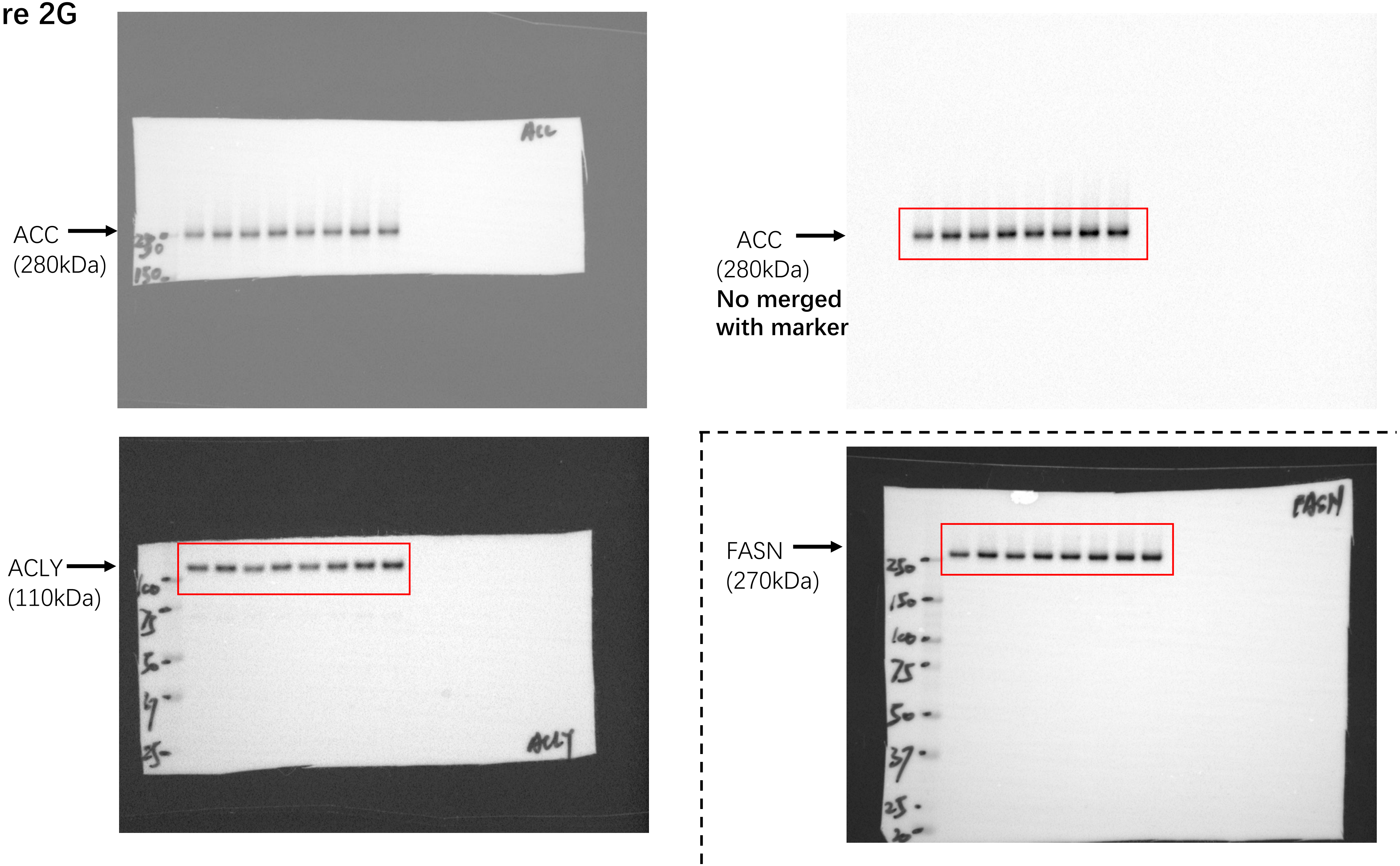


ACC and ACLY were from the same membrane.

FASN was from another membrane.

**Figure 2G**

β-actin
(42kDa)

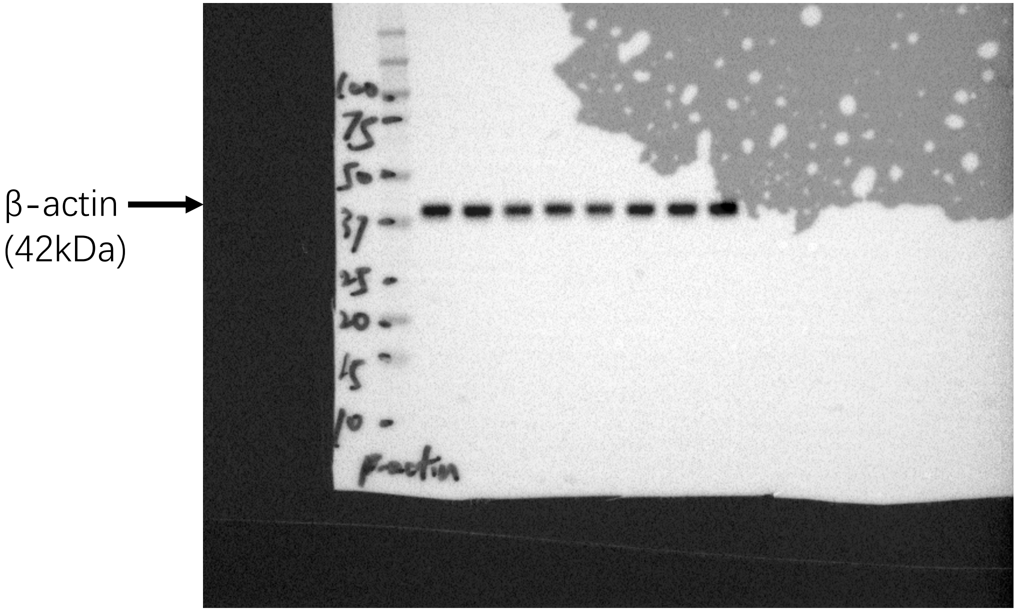


**β-actin**
**(42kDa)**
**No merged**
**with marker**

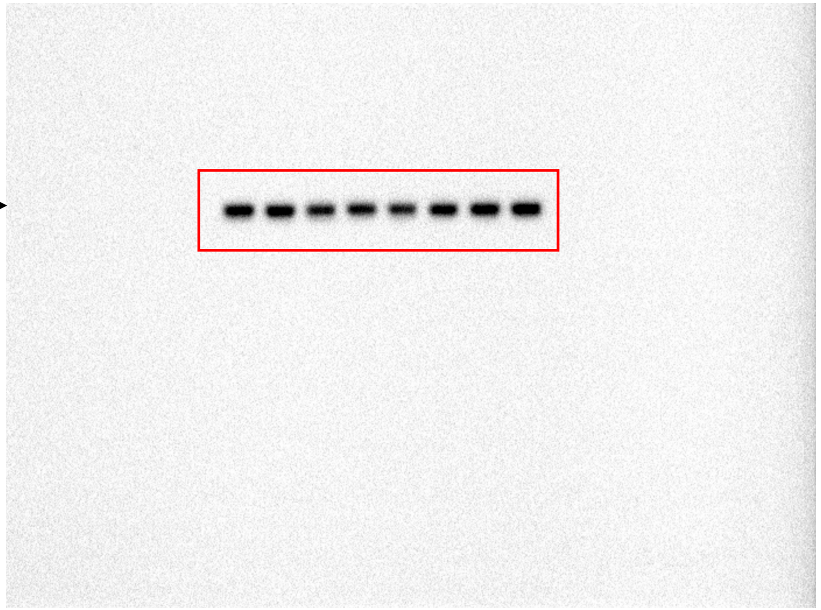

β-actin was from another membrane.

**Figure 3C**

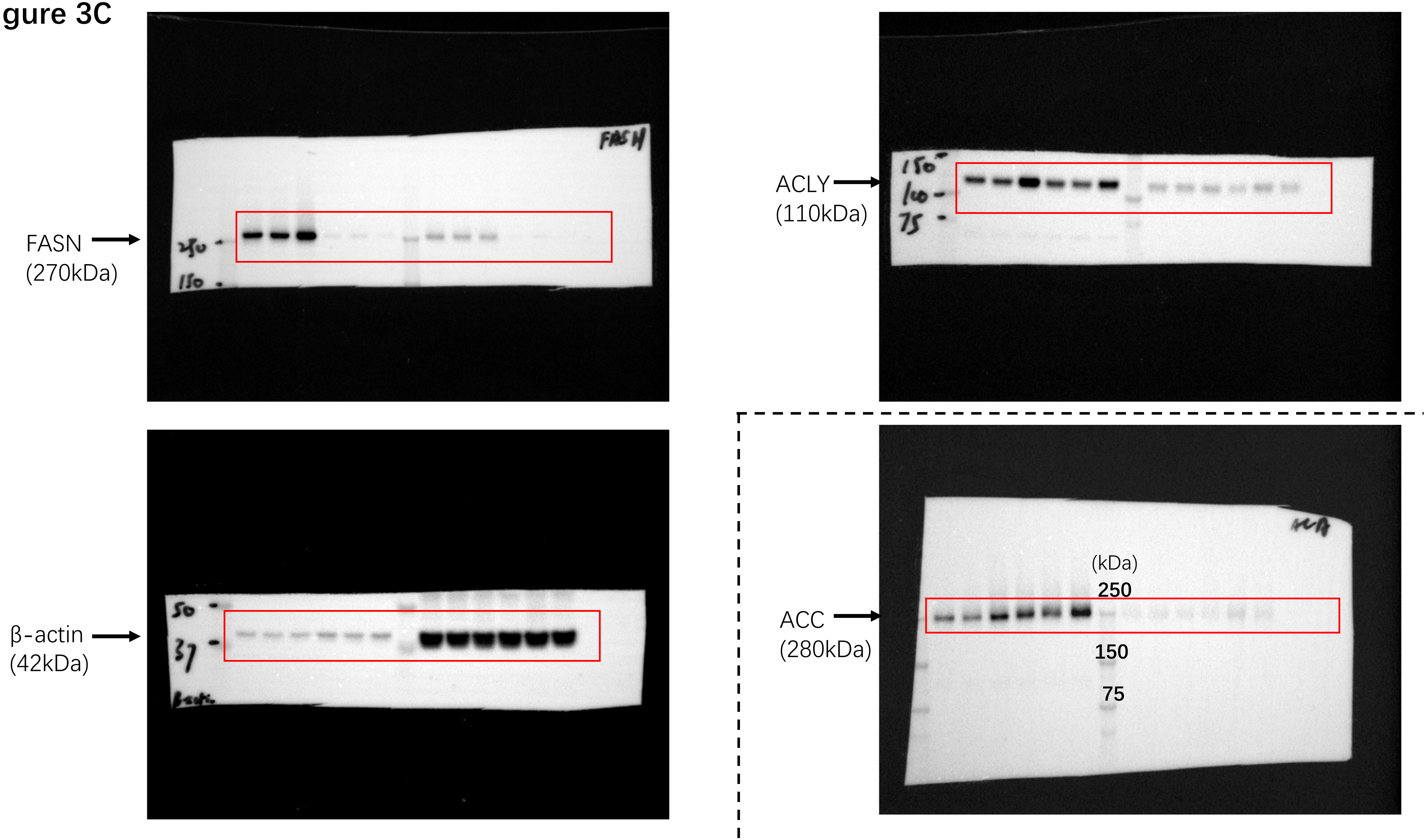


FASN, ACLY and β-actin were from the same membrane.

ACC was from another membrane.

**Figure 7C**

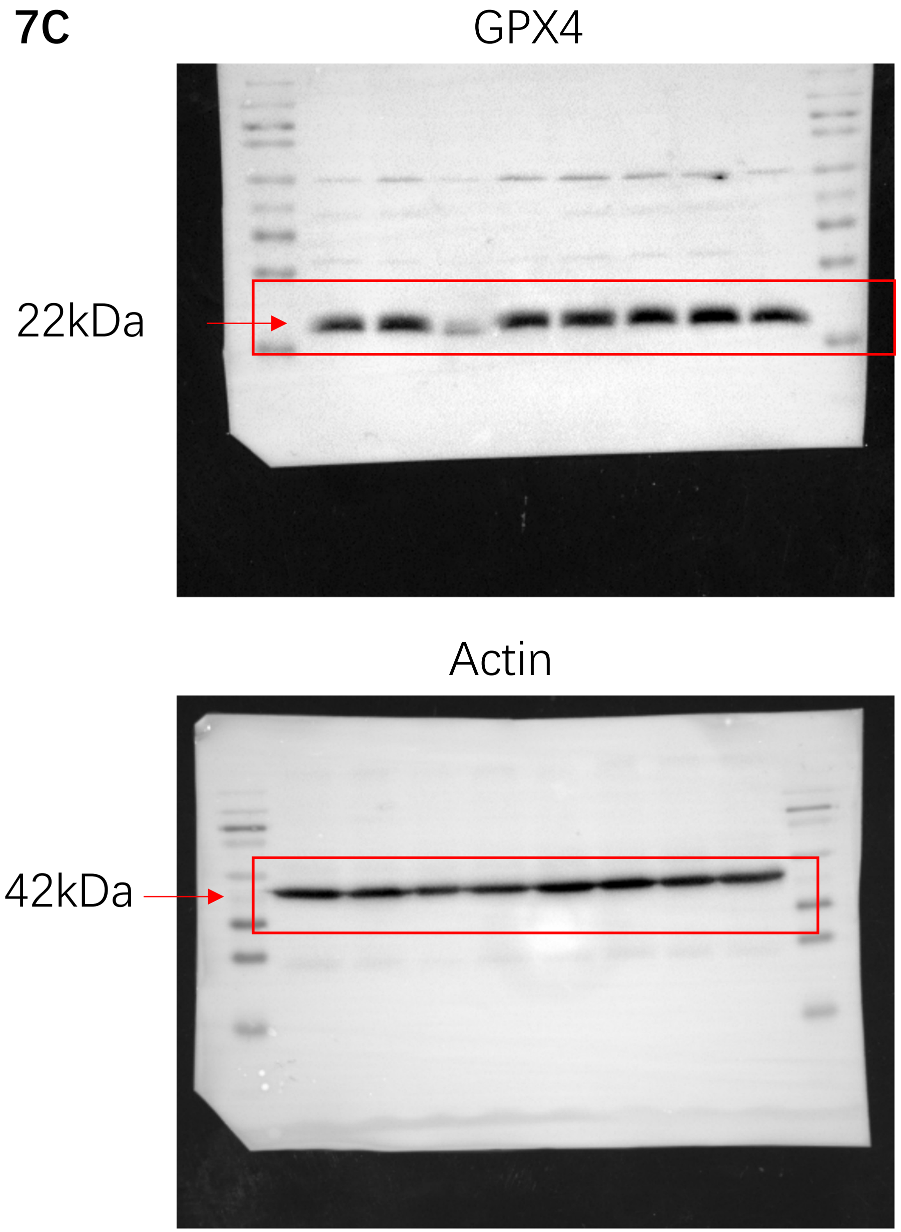


GPX4 and actin run on same gel/membrane after stripping

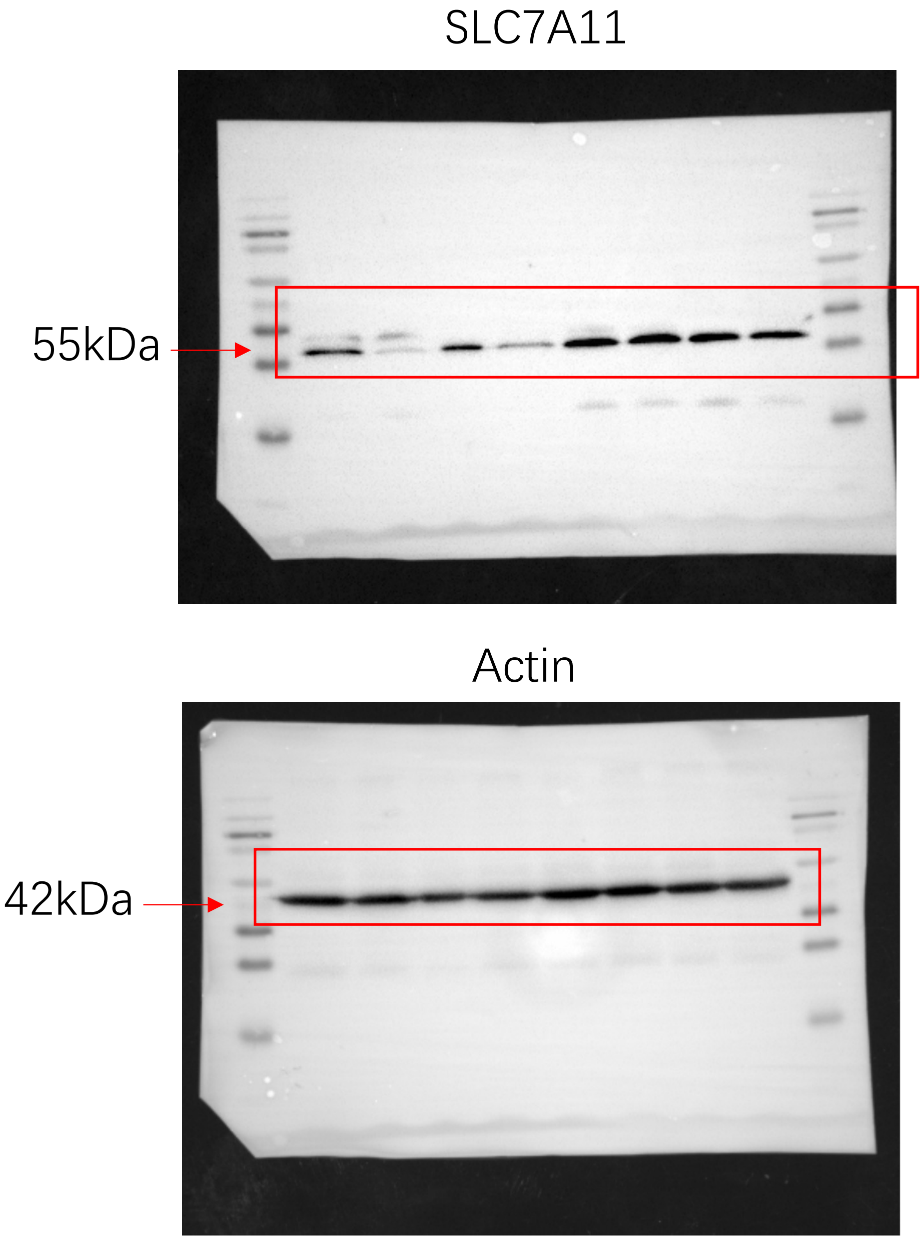


SLC7A11 and actin run on same gel/membrane after stripping

# Supplemental Figures

**SFig2 E**

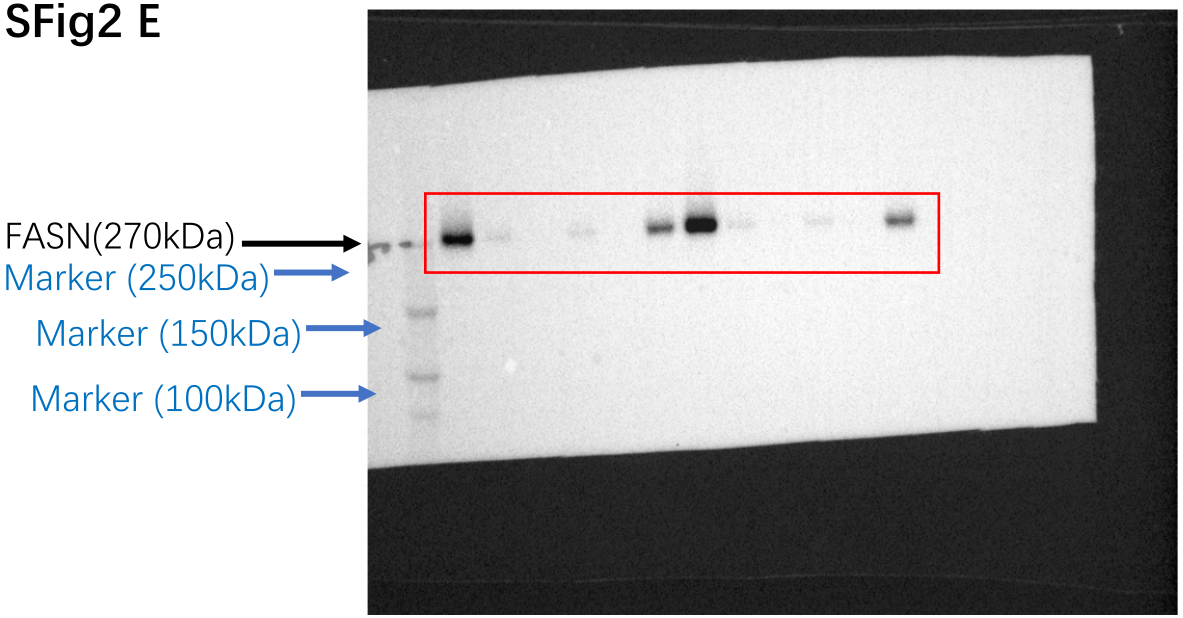


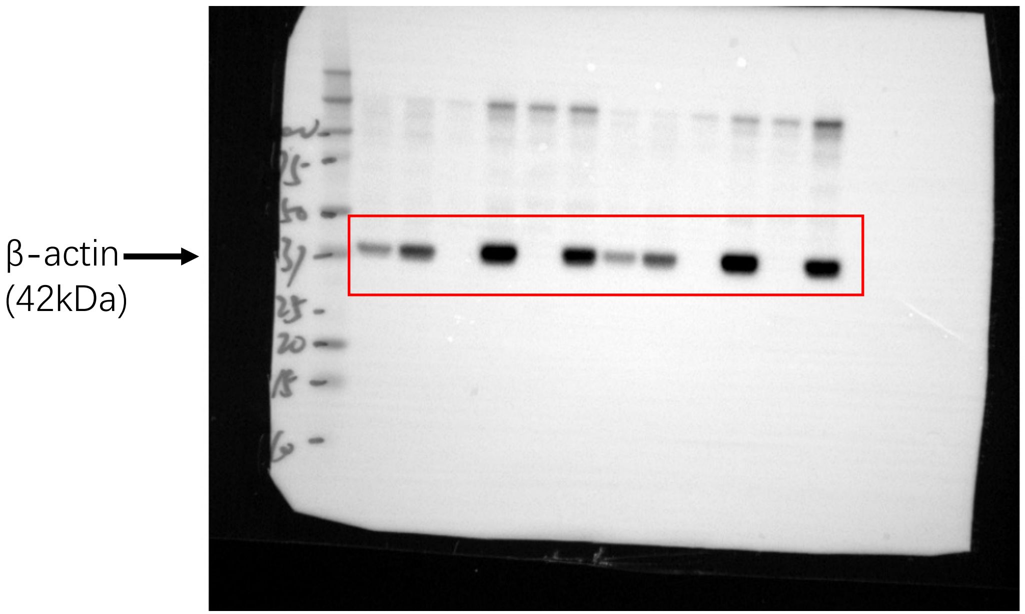


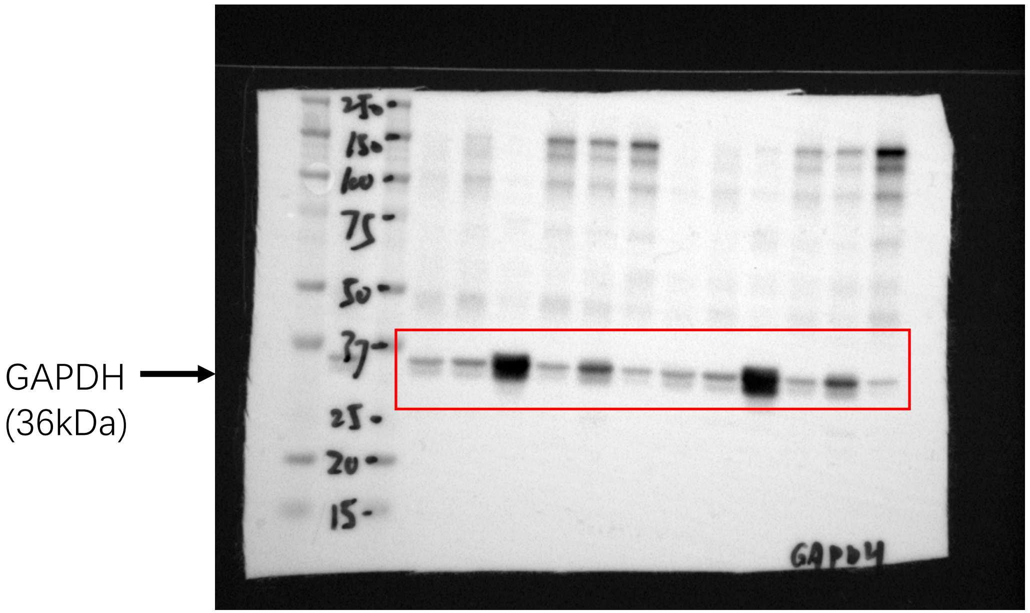


FASN, ACLY and β-actin were from different membrane. For FASN 15μg/8ul sample was added to each lane, for β-actin and GAPDH 11.25 μg/6ul sample was added to each lane.

**SFig2 F**

FASN
(270kDa)

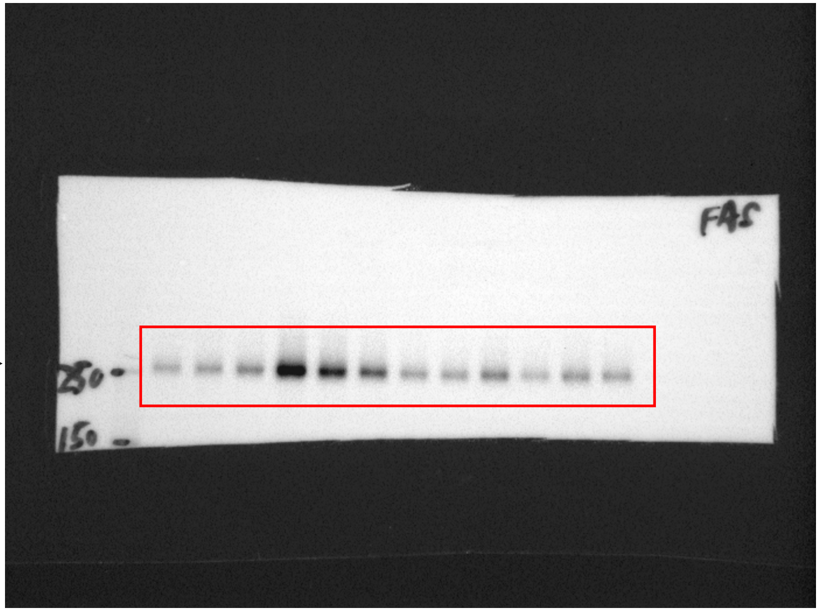


ACLY
(110kDa)

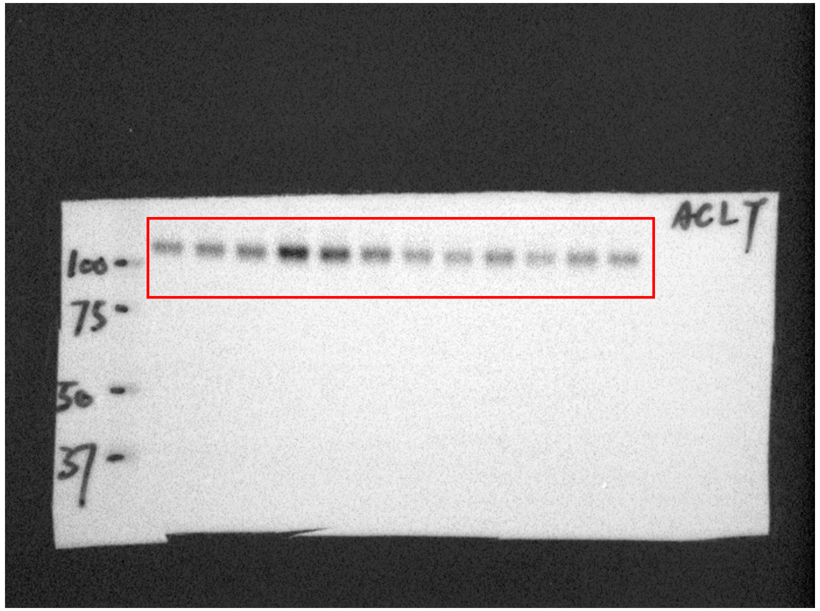


FASN and ACLY were from the same membrane.

## SFig2 F

ACC (280kDa)

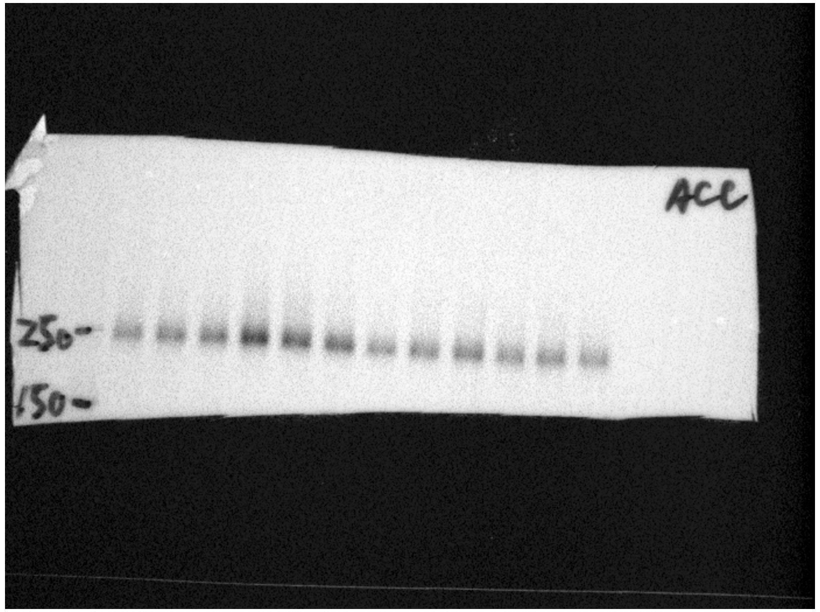


ACC(280kDa) No merged with marker

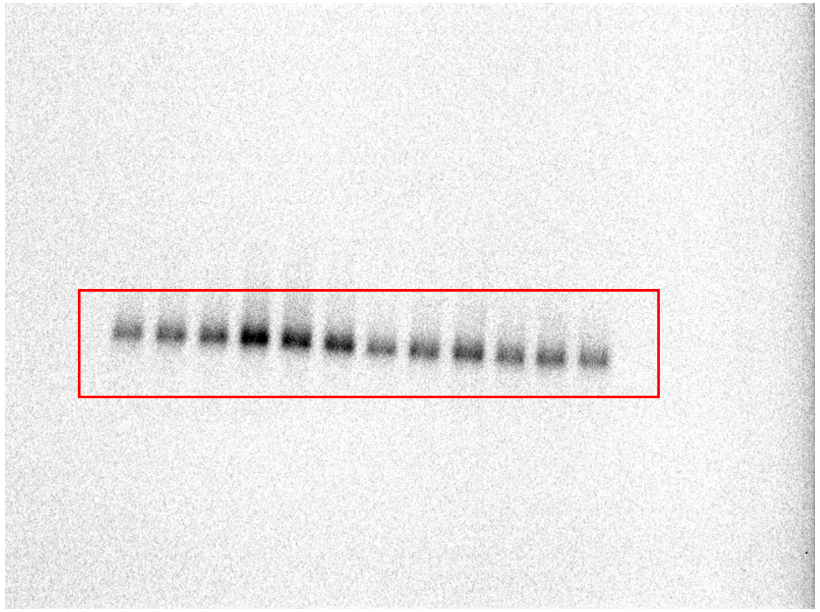

β-actin (42kDa)

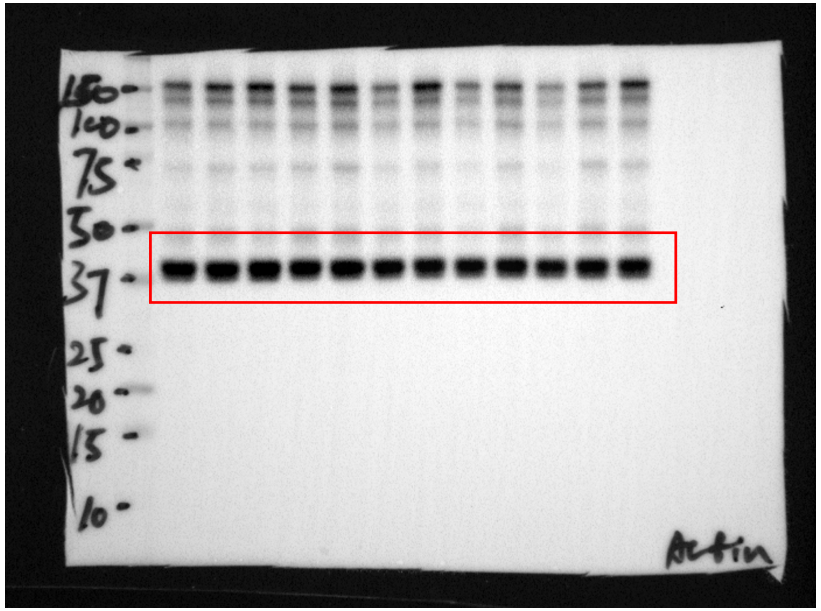


ACC and β-actin were from the same membrane.

## SFigure 3A primary AEC2

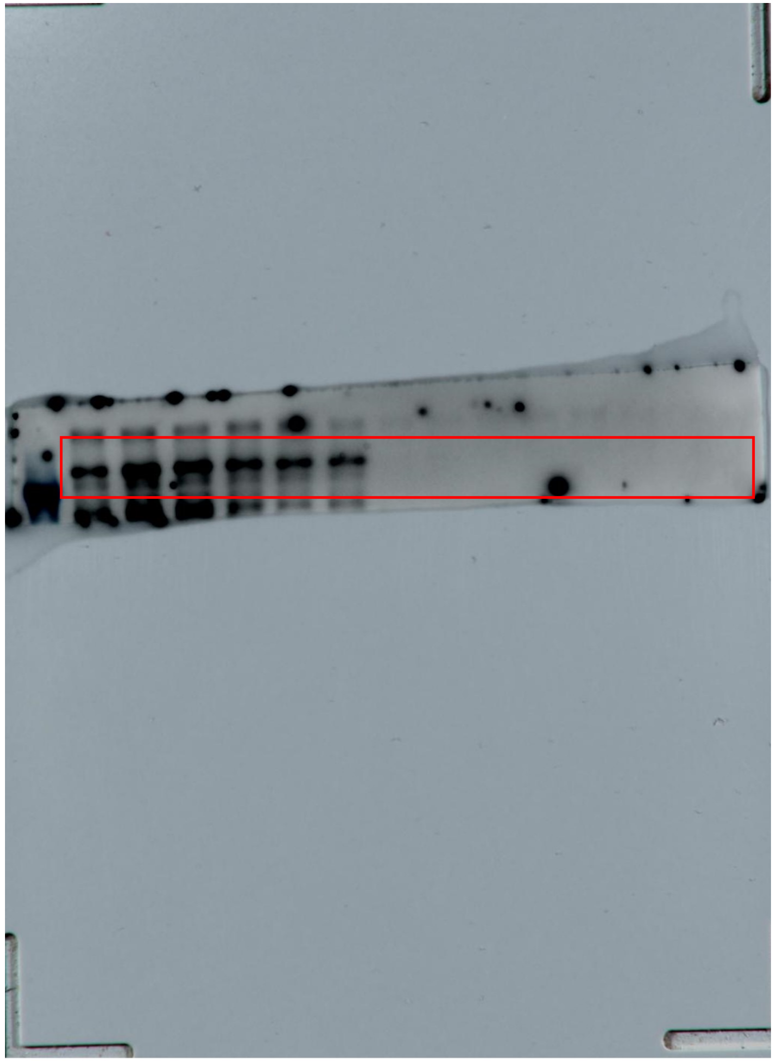


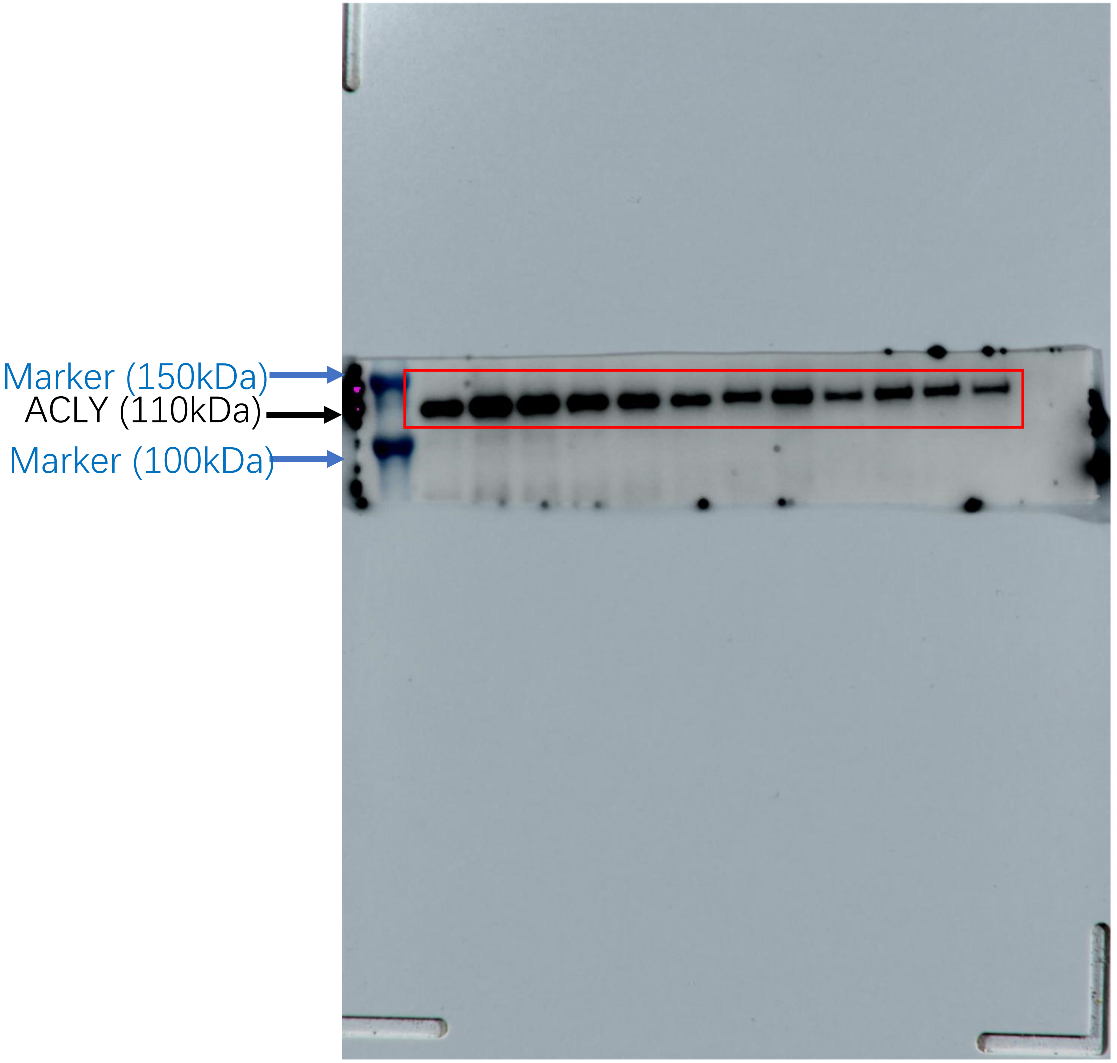


FASN and ACLY were from the same membrane.

# SFigure 3A primary AEC2

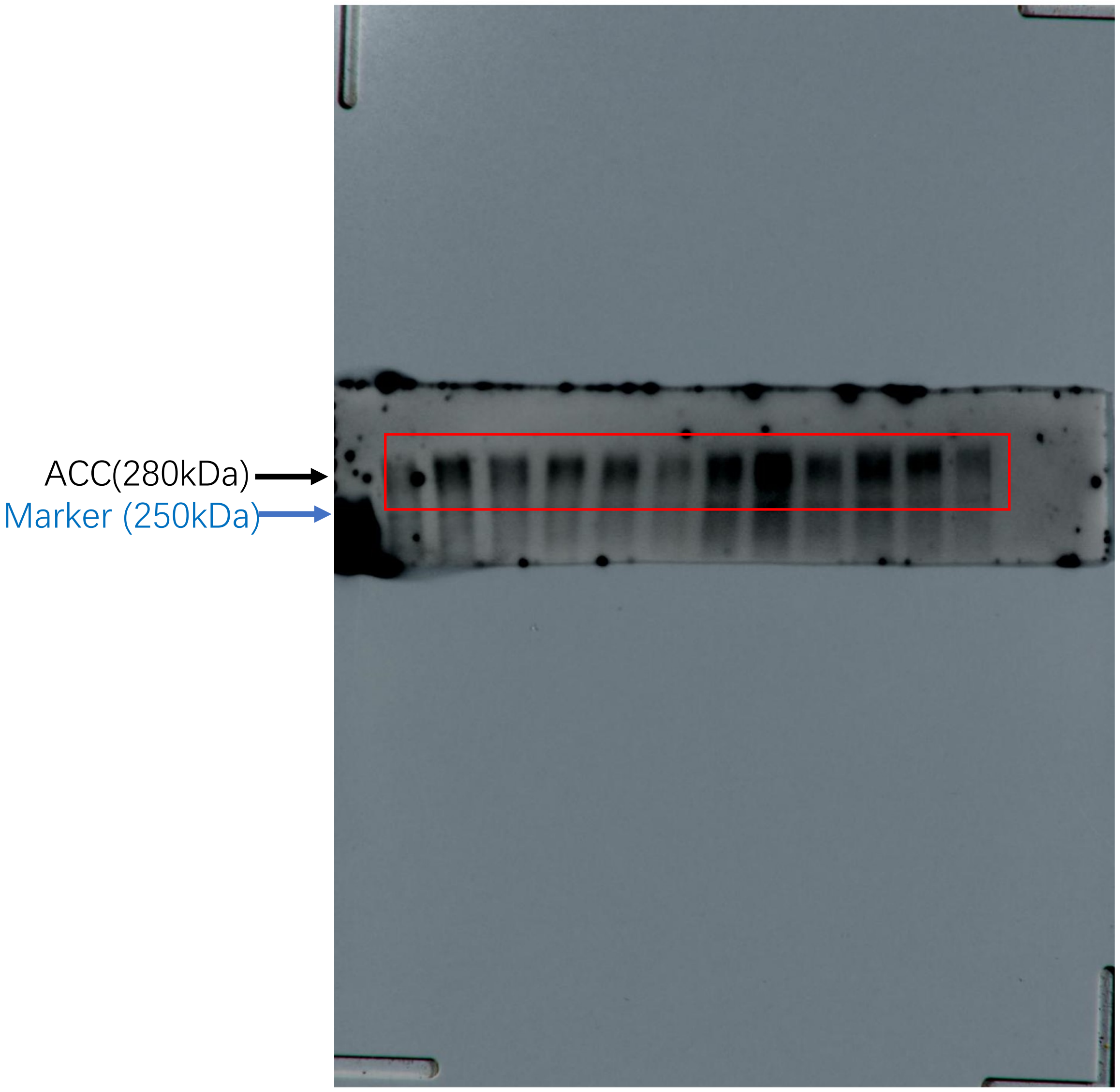


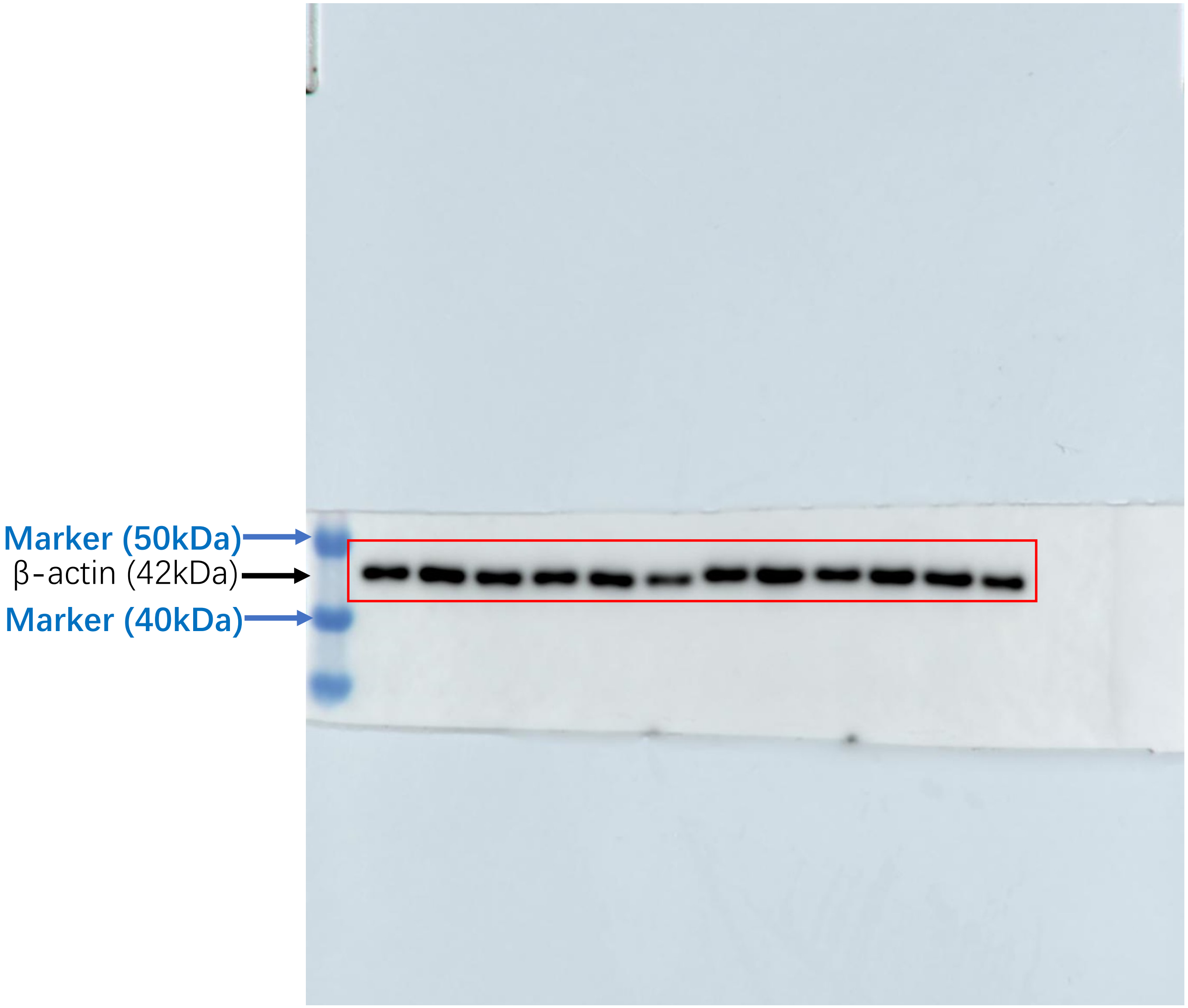


ACC and β-actin were from the same membrane.

**SFig4 D**

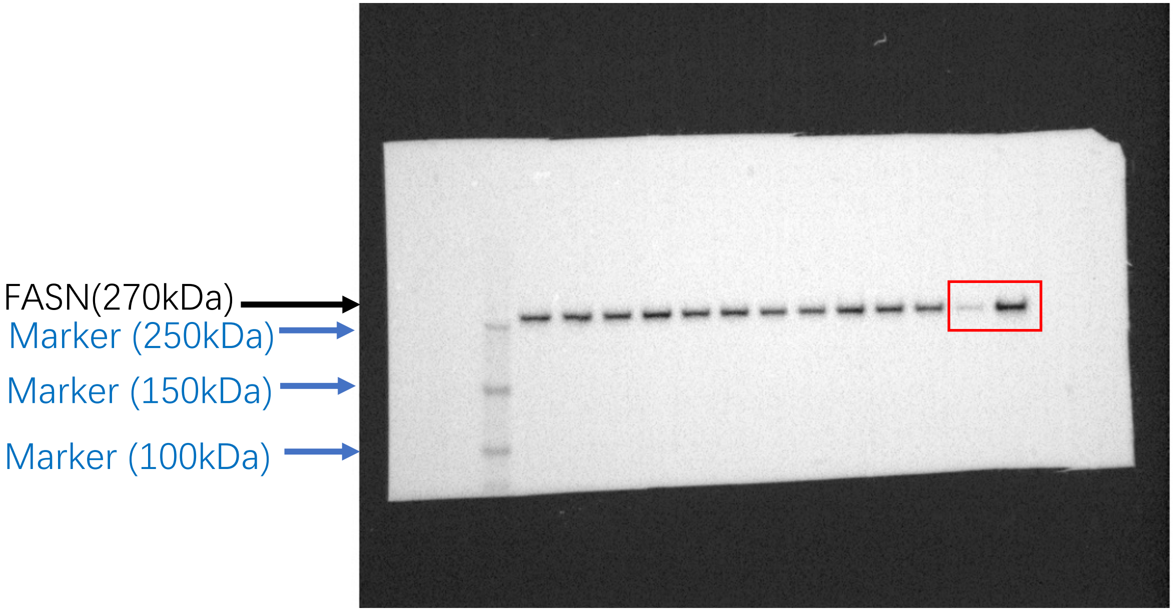


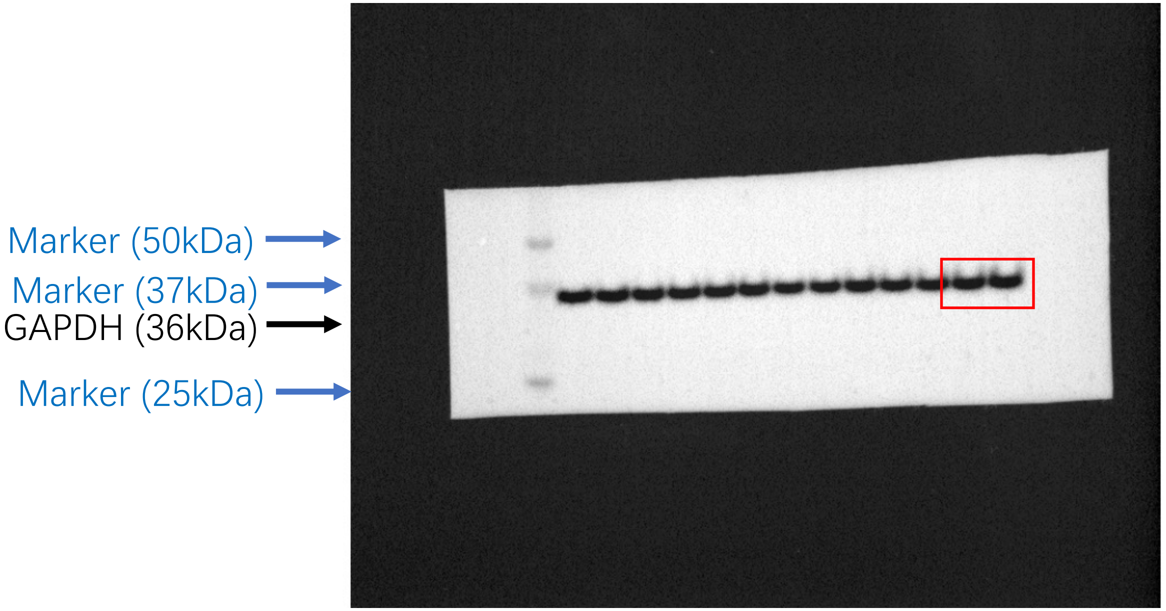


FASN and GAPDH were from the same membrane.

## SFig4 F

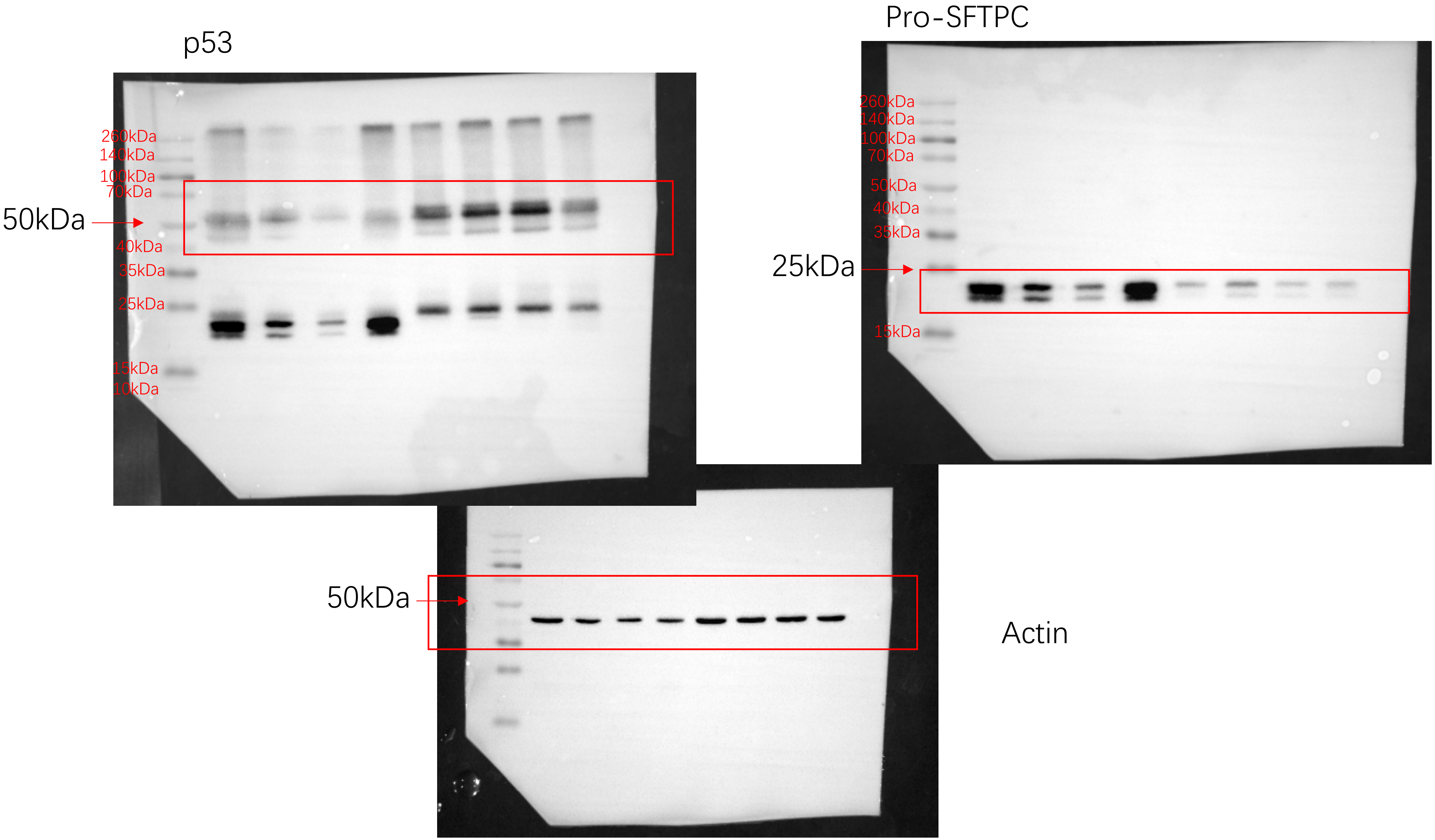

p53
260kDa
140kDa
100kDa
70kDa
50kDa
40kDa
35kDa
25kDa
15kDa
10kDa
Pro-SFTPC
260kDa
140kDa
100kDa
70kDa
50kDa
40kDa
35kDa
25kDa
15kDa
50kDa
Actin
